# Lung Adenocarcinoma Promotes NETosis via the NPM1–TNFAIP6–CD44–SPP1 Axis

**DOI:** 10.3390/cancers18061023

**Published:** 2026-03-22

**Authors:** Renwang Liu, Zixuan Hu, Mingbiao Li, Shen Yang, Jianfang Wang, Zhanrui Zhang, Long Yang, Jun Chen

**Affiliations:** 1 Department of Lung Cancer Surgery, Center of Thoracic Surgery, Tianjin Medical University General Hospital, Tianjin 300052, China; 2 Tianjin Key Laboratory of Lung Cancer Metastasis and Tumor Microenvironment, Tianjin Lung Cancer Institute, Tianjin Medical University General Hospital, Tianjin 300052, China; 3 School of Integrative Medicine, Tianjin University of Traditional Chinese Medicine, Tianjin 301617, China; 17720005568@163.com; 4 Research Center for Infectious Diseases, Tianjin University of Traditional Chinese Medicine, Tianjin 301617, China; 5 School of Public Health, Tianjin University of Traditional Chinese Medicine, Tianjin 301617, China

**Keywords:** NETosis, neutrophil extracellular traps, lung adenocarcinoma, TNFAIP6, protein–protein interaction, competitive binding, transcriptional regulation

## Abstract

**Background:** While neutrophil extracellular traps (NETs) have been shown to contribute to cancer progression, including that of lung adenocarcinoma, the mechanisms underlying NET formation within the tumor immune microenvironment (TIME) remain incompletely understood. Notably, neutrophil infiltration has been strongly linked to tumor necrosis factor alpha-inducible protein 6 (TNFAIP6) expression. **Methods:** In vitro and in vivo experiments were performed. DNA pulldown coupled with mass spectrometry, bioinformatics analyses, immunohistochemistry, and dual-luciferase reporter assays were conducted. DNA pulldown Western blotting, chromatin immunoprecipitation–quantitative PCR, and dual-luciferase reporter assays using truncated promoter constructs were also employed. **Results:** TNFAIP6 expressed by lung adenocarcinoma cells was shown to induce NET formation (a form of programmed cell death called NETosis). Mechanistically, TNFAIP6 interacted with CD44 in lung adenocarcinoma cells, leading to increased extracellular availability of secreted phosphoprotein 1 (SPP1) within the TIME and the subsequent promotion of NETosis. Additionally, nucleophosmin 1 (NPM1) significantly enhances the transcriptional activation of *TNFAIP6* and associates with the −2000 to −1700 bp region of its promoter. **Conclusions:** These findings delineate a regulatory model in which lung adenocarcinoma cells directly stimulate NETosis through the NPM1–TNFAIP6–CD44–SPP1 axis, suggesting that therapeutic targeting of this pathway may attenuate tumor progression.

## Simple Summary

Neutrophil extracellular traps (NETs) promote cancer progression, but the regulatory mechanism underlying their formation remains unclear. This study shows that tumor necrosis factor alpha-inducible protein 6 (TNFAIP6) derived from lung adenocarcinoma induces NET formation (NETosis) in vitro and in vivo. TNFAIP6 interacts with CD44, leading to increased extracellular secreted phosphoprotein 1 (SPP1) levels and subsequent NETosis. Additionally, nucleophosmin 1 (NPM1) upregulates the transcriptional activation of *TNFAIP6* by interacting with the −2000 to −1700 bp region of its promoter. Together, these results indicate that the NPM1–TNFAIP6–CD44–SPP1 axis is a critical regulator of NETosis in lung adenocarcinoma, highlighting this pathway as a potential therapeutic target for suppressing tumor progression.

## 1. Introduction

Lung adenocarcinoma has become the most prevalent histological subtype of lung cancer in recent decades [1,2]. Despite substantial advances in therapeutic strategies, many patients with lung adenocarcinoma still face unfavorable clinical outcomes due to disease progression and metastasis [3,4]. The tumor immune microenvironment (TIME) plays a critical role in this process and is extensively involved in key oncogenic events, including immune evasion, local recurrence, and distant metastasis [5,6,7].

Neutrophils represent a major cellular component of the TIME [8,9,10]. These cells can release neutrophil extracellular traps (NETs), which are composed of decondensed chromatin DNA, histones, and granule-derived proteins, through a specialized form of cell death known as neutrophil extracellular trap formation (NETosis) [11,12]. Accumulating evidence indicates that NETs within tumors, including lung adenocarcinoma, promote disease progression through multiple mechanisms [13,14,15]. NETs can ensnare circulating tumor cells to facilitate metastasis [16], release mediators that enhance angiogenesis [17], and suppress natural killer cell activity, thereby contributing to immune escape [18]. Consequently, inhibition of NET formation in tumors may mitigate their pro-tumorigenic effects and impede cancer progression.

Despite these observations, the mechanisms governing NETosis in the tumor context remain poorly defined. Previous work demonstrated that the expression of tumor necrosis factor alpha-inducible protein 6 (TNFAIP6) in lung adenocarcinoma is associated with neutrophil infiltration, polarization, and suppression of early apoptosis [19]. In addition, TNFAIP6 increases the proportion of neutrophils exhibiting “late-stage apoptosis,” as indicated by Annexin V and propidium iodide double positivity (Figure S1A) [19]. Because this phenotype may reflect necrosis, pyroptosis, or NETosis, neutrophils were treated with lung adenocarcinoma cell supernatants, and neutrophil-derived mediators were quantified by enzyme-linked immunosorbent assay (ELISA). TNFAIP6 did not significantly affect interleukin-18 (IL-18) or interleukin-1β (IL-1β) levels, but markedly increased myeloperoxidase (MPO) concentrations in neutrophil supernatants (Figure S1B–D). These findings suggest a potential role for TNFAIP6 in promoting NETosis rather than inflammatory cell death pathways.

Based on these preliminary observations, we hypothesized that TNFAIP6 expressed by lung adenocarcinoma cells plays a pivotal role in inducing NETosis within the TIME. Accordingly, we employed in vitro and in vivo approaches to systematically evaluate the effects of TNFAIP6 on NET formation, elucidate the underlying molecular mechanisms, and identify key functional regulatory sites.

## 2. Materials and Methods

### 2.1. Cell Culture, Transient Transfection, and Stable Transduction

Cell culture and genetic manipulations were performed as previously described [19,20,21]. The A549, H1975, BEAS-2B, Lewis lung carcinoma (LLC), and HEK293T cell lines were obtained from the American Type Culture Collection (ATCC). PC9 and H1299 cells were purchased from the Institute of Biochemistry and Cell Biology, Chinese Academy of Sciences (Shanghai, China). All cell lines were maintained in RPMI 1640 medium (Gibco, Grand Island, NY, USA), except for HEK293T and LLC cells, which were cultured in DMEM (Gibco). All media were supplemented with 10% fetal bovine serum, 100 IU/mL penicillin, and 100 μg/mL streptomycin (Invitrogen, Carlsbad, CA, USA).

Small interfering RNAs targeting *TNFAIP6*, secreted phosphoprotein 1 (*SPP1*), nucleophosmin 1 (*NPM1*), and *ENO1*, as well as a negative control siRNA, were purchased from RiboBio (Guangzhou, China). Overexpression plasmids for *TNFAIP6*, *SPP1*, and *NPM1*, together with lentiviral vectors encoding *Tnfaip6* and corresponding control vectors, were constructed by GeneChem (Shanghai, China). To generate stable cell lines, LLC cells were transduced with LV-Tnfaip6 or LV-negative control according to the manufacturer’s instructions. Transient transfections were performed using Lipofectamine 2000 (Invitrogen) following the supplier’s protocol.

### 2.2. Neutrophil Isolation and Indirect Co-Culture

Neutrophil isolation and indirect co-culture assays were conducted as previously reported [19]. Briefly, neutrophils were isolated from the peripheral blood of healthy volunteers using Polymorphprep (Axis-Shield, Dundee, Scotland). Cell purity was assessed using a Fast Giemsa Stain Kit (Yeasen, Shanghai, China), prior to culture in RPMI 1640 medium (Gibco). Conditioned media from A549 and PC9 cells were collected 24 h after transfection and applied to freshly isolated neutrophils for 16 h.

### 2.3. Western Blotting and qPCR

Western blotting and quantitative PCR (qPCR) were performed as described previously [19]. The following primary antibodies were used: anti-TNFAIP6 (1:1000; Proteintech, Wuhan, China), anti-SPP1 (1:1000; Proteintech), anti-NPM1 (1:20,000; Proteintech), anti-CD44 (1:20,000; Proteintech), anti-histone H3 (1:2000; Proteintech), and anti-GAPDH (1:5000; Proteintech). All uncropped blots and molecular weight markers are shown in Supplementary Materials S1. For qPCR analysis, total RNA was extracted using TRIzol reagent (Invitrogen), and complementary DNA was synthesized with the PrimeScript RT Reagent Kit (TaKaRa, Beijing, China). Primer sequences were reported previously and are provided in Table S1 [19,22].

### 2.4. Immunofluorescence Assay

Immunofluorescence assays were performed as previously described [23]. Briefly, cells cultured on coverslips were fixed with 4% paraformaldehyde for 30 min, permeabilized with 0.5% Triton X-100 for 15 min, and blocked with 1% bovine serum albumin in phosphate-buffered saline for 1 h. Samples were incubated with primary antibodies overnight at 4 °C, followed by incubation with Alexa Fluor-conjugated secondary antibodies for 1 h at room temperature. Nuclei were counterstained with DAPI (Sigma-Aldrich, St. Louis, MO, USA) for 5 min, and images were acquired using a fluorescence microscope. Primary antibodies included anti-citrullinated histone H3 (Histone H3 [citrulline Arg17, citrulline Arg2, citrulline Arg8]; 1:200; NB100-57135, Novus, Chesterfield, MO, USA) and anti–myeloperoxidase (MPO; 5 μg/mL; AF3667, R&D Systems, Minneapolis, MN, USA).

### 2.5. Immunohistochemistry

Immunohistochemical staining was performed as described previously [19]. Briefly, tissue sections were deparaffinized, subjected to antigen retrieval in 5 mM Tris-HCl buffer using microwave heating for 10 min, and treated with 3% hydrogen peroxide to quench endogenous peroxidase activity. After serum blocking, the sections were incubated with primary antibodies overnight at 4 °C, followed by incubation with horseradish peroxidase-conjugated secondary antibodies for 30 min at room temperature. Signal development was achieved using diaminobenzidine, and sections were counterstained with hematoxylin.

The primary antibodies used included anti-citrullinated histone H3 (Histone H3 [citrulline Arg17, citrulline Arg2, citrulline Arg8]; 1:200; NB100-57135, Novus), anti-SPP1 (1:250; Proteintech), anti-TNFAIP6 (1:250; Proteintech), and anti-NPM1 (1:250; Proteintech). For citrullinated histone H3 (Cit H3) staining, positive sites were quantified by counting. For all other markers, staining was evaluated using a semiquantitative scoring system independently applied by two pathologists, based on staining intensity (0–3) and the proportion of positive cells (1–4), with the sum yielding a final score ranging from 1 to 7.

### 2.6. Syngeneic Tumor Model

To examine direct interactions between neutrophils and tumor cells in the absence of adaptive immune influences, syngeneic tumor models were established using C57BL/6 nude mice. LLC cells stably overexpressing Tnfaip6 were generated as described above. A total of 5 × 10^5^ LLC cells suspended in 50% Matrigel (Corning, Corning, NY, USA) were subcutaneously injected into the left lower abdomen of each mouse (*n* = 6 per group). Deoxyribonuclease I (DNase I, 0.1 U per mouse) was intraperitoneally injected on day 6 after inoculation and once daily for 3 consecutive days to verify the role of NETs in the influence of TNFAIP6 on tumor growth. Tumor length and width were measured every 3 days using a vernier caliper. The mice were euthanized 21 days after implantation, and tumors were excised for analysis. TNFAIP6 expression in tumor tissues was verified by immunohistochemistry (Figure S2D).

### 2.7. Enzyme-Linked Immunosorbent Assay

Following the collection of culture supernatants, the concentrations of SPP1, MPO, and IL-1β (MultiSciences, Hangzhou, China), IL-18 (Proteintech), as well as Cit H3 (Cayman, Ann Arbor, MI, USA), were measured using ELISA kits according to the manufacturers’ instructions. Standard curves were generated using a four-parameter logistic (4PL) regression model implemented in Python 3.13.2. Supernatants used for MPO measurement were diluted 100-fold, those for Cit H3 measurement were diluted 10-fold, and samples for all other analytes were analyzed without dilution.

### 2.8. Co-Immunoprecipitation

Co-immunoprecipitation (Co-IP) assays were performed as previously described [24]. To standardize total protein input across samples, cells were trypsinized and counted using a TC20 automated cell counter (Bio-Rad, Hercules, CA, USA), and the volume of RIPA lysis buffer was adjusted accordingly. Cell lysates were incubated with antibodies against SPP1 (22952-1-AP, Proteintech), TNFAIP6 (ab267469, Abcam, Cambridge, UK), CD44 (15675-1-AP, Proteintech), or normal IgG (Beyotime, Shanghai, China) at 4 °C for 24 h. Protein A/G agarose beads (Beyotime) were then added, and the samples were gently rotated for an additional 3 h at 4 °C. After centrifugation, the supernatant was discarded, and the beads were washed five times with lysis buffer. Immunoprecipitated proteins were eluted in SDS loading buffer, boiled at 100 °C for 10 min, and analyzed by Western blotting following centrifugation.

### 2.9. Recombinant Protein Preparation

All plasmids were constructed by GeneChem. His-tagged *TNFAIP6* and *SPP1* expression plasmids were generated for eukaryotic expression and transfected into HEK293T cells. Because TNFAIP6 and SPP1 are secreted proteins, culture supernatants were collected for protein purification. His-tagged fusion proteins were purified using a His-tag Protein Purification Kit (IDA–Ni agarose magnetic beads; Beyotime) according to the manufacturer’s instructions.

### 2.10. DNA Pulldown Assay

Biotin-labeled and unlabeled DNA probes were amplified by PCR using the primers listed in Table S1. PCR products were resolved by 1% agarose gel electrophoresis, excised under UV illumination, and purified using a gel DNA extraction kit (TaKaRa, Kusatsu, Japan).

For DNA pulldown assays, purified DNA probes were incubated with prewashed streptavidin magnetic beads (Solarbio, Beijing, China) for 30 min at room temperature. After two washes, bead–probe complexes were incubated with nuclear extracts at 4 °C for 4 h under gentle rotation. Following elution and centrifugation, supernatants were subjected to Western blot analysis.

### 2.11. Silver Staining and Liquid Chromatography–Mass Spectrometry

Proteins captured by *TNFAIP6* promoter probe pulldown were visualized by silver staining using a fast silver stain kit (Beyotime) according to the manufacturer’s instructions. Nuclear extracts served as input controls, and pulldown products obtained with unlabeled probes were used as negative controls. Protein identification was performed by liquid chromatography–mass spectrometry (LC–MS) at Lumingbio (Shanghai, China). Briefly, protein samples were digested and desalted, separated using an EASY-nLC 1200 nano-HPLC system (Thermo Scientific, Waltham, MA, USA), and analyzed on a Fusion mass spectrometer (Thermo Scientific) in data-dependent acquisition mode. Data were processed using Proteome Discoverer software (version 2.5) for database searching. The raw data have been deposited in Figshare (https://doi.org/10.6084/m9.figshare.31033222).

### 2.12. Bioinformatics Analysis and Prediction

Differential gene expression and survival analyses were conducted using The Cancer Genome Atlas (TCGA) datasets with R software (version 3.6.4), as previously described [21]. Optimal gene expression cutoffs were determined using the MaxStat package, and differences in overall survival and the progression-free interval were analyzed using the survfit function. Lists of known transcription factors were obtained from the TRRUST database [25]. Protein–protein interaction predictions were performed using the STRING database [26]. Protein structures and predicted interaction interfaces for SPP1, TNFAIP6, and CD44 were generated using the AlphaFold Server [27].

### 2.13. Dual-Luciferase Reporter Assay

Wild-type and truncated fragments of the *TNFAIP6* promoter were cloned into the GV238 firefly luciferase reporter vector by GeneChem. Plasmids were transfected into cells using Lipofectamine 3000 (Invitrogen). A Renilla luciferase-expressing plasmid (CV045) was co-transfected as an internal control for normalization. Forty-eight hours after transfection, luciferase activities were measured using a GloMax^®^ 20/20 luminometer (Promega, Madison, WI, USA) in accordance with the manufacturer’s instructions.

### 2.14. Chromatin Immunoprecipitation-qPCR

Chromatin immunoprecipitation (ChIP) assays were performed using the BeyoChIP™ ChIP Assay Kit (Beyotime) following the manufacturer’s protocol. Briefly, cells were cross-linked with formaldehyde for 10 min, lysed on ice, and sonicated to shear chromatin DNA. Immunoprecipitation was conducted using an anti-NPM1 antibody (10306-1-AP, Proteintech) or normal IgG as a control, followed by overnight incubation at 4 °C with rotation. Protein A/G magnetic beads conjugated with salmon sperm DNA (80 μL) were then added, and samples were rotated for an additional 1 h at 4 °C. Bead-bound complexes were sequentially washed five times with the provided buffers. After elution, cross-links were reversed by incubation with 5 M NaCl at 65 °C for 4 h. DNA was subsequently purified and analyzed by quantitative PCR. The primer sequences used for ChIP-qPCR are listed in Table S1.

### 2.15. Protein Degradation and mRNA Stability Assay

For protein degradation assays, cells were treated with cycloheximide (CHX; MedChemExpress, Monmouth Junction, NJ, USA) at a final concentration of 100 μg/mL to inhibit protein synthesis. The cells were harvested 0, 3, 6, and 9 h after CHX treatment, and cell lysates were analyzed using Western blotting to assess TNFAIP6 protein levels. Band intensities were quantified using ImageJ(version 1.53e) software. Protein half-life was calculated using the formula: K = [ln(C0) − ln(C9)]/(t9 − t0); t1/2 = 0.693/K.

For mRNA stability assays, actinomycin D (Psaitong, Zaozhuang, China), an inhibitor of RNA transcription, was added to the culture medium at a final concentration of 200 nM. Cells were collected 0, 3, 6, 9, and 12 h after treatment for RNA extraction. *Tnfaip6* mRNA levels at each time point were quantified by qPCR. The mRNA half-life was calculated using the formula K = [ln(C0) − ln(C12)]/(t12 − t0); t1/2 = 0.693/K.

### 2.16. Statistical Analysis and Data Visualization

Statistical analyses were performed using R software (version 3.6.4) and GraphPad Prism (version 9.5), depending on the dataset. Student’s t tests were applied to data with equal variance, whereas Mann–Whitney U tests were used for data with unequal variance. Pearson correlation coefficients were used for correlation analyses, and survival differences were assessed using the log-rank test. Statistical significance was defined as *p* < 0.05. Protein–protein interactions were predicted using the STRING database [26]. The schematic diagram in this study was created using BioGDP.com [28].

## 3. Results

### 3.1. TNFAIP6 in Lung Adenocarcinoma Promotes NETosis in Neutrophils

**Figure 1:**
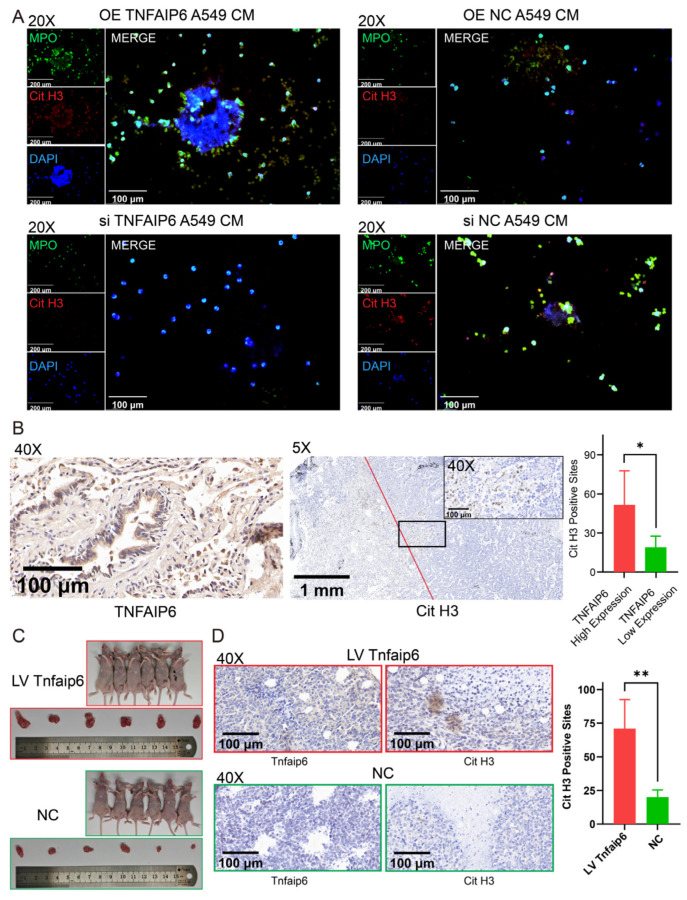
TNFAIP6 in lung adenocarcinoma promotes NET formation. (**A**) Neutrophils were co-cultured with conditioned medium (CM) derived from A549 cell supernatants. Immunofluorescence staining demonstrated that TNFAIP6 overexpression in A549 cells increased myeloperoxidase (MPO, green) and citrullinated histone H3 (Cit H3, red) signals, accompanied by an increase in extracellular DNA, as indicated by DAPI staining (blue). Similar results were observed in PC9 cells (Figure S2A). (**B**) Immunohistochemical staining revealed a higher number of Cit H3-positive sites in lung adenocarcinoma tissues with high TNFAIP6 expression. Representative images are shown from patient No. 08. (**C**) In vivo, lentiviral (LV) Tnfaip6 overexpression increased the size of syngeneic tumors established using Lewis lung carcinoma (LLC) cells in C57BL/6 nude mice. Nude mice were selected to minimize lymphocyte-mediated effects while preserving neutrophil function. *Tnfaip6* transduction efficiency was confirmed using immunohistochemistry (Figure S2D). (**D**) Immunohistochemical analysis demonstrated that LV Tnfaip6 significantly increased the number of Cit H3-positive sites within tumor tissues. * *p*-value < 0.05, ** *p*-value < 0.01.

To investigate the role of TNFAIP6 in NETosis, immunofluorescence staining was performed to detect NET-specific markers, including Cit H3 and MPO, in neutrophils cultured with lung adenocarcinoma-conditioned medium. Overexpression of TNFAIP6 in A549 and PC9 cells markedly enhanced NET formation in co-cultured neutrophils, whereas TNFAIP6 knockdown significantly suppressed NETosis (Figure 1A and Figure S2A). ELISAs also revealed that the overexpression of TNFAIP6 in A549 and PC9 cells significantly increased the Cit H3 concentration in co-cultured neutrophils, whereas TNFAIP6 knockdown decreased it and BEAS-2B had no such effect (Figure S2B).

In addition, ten freshly obtained lung adenocarcinoma specimens, collected previously [19], were subjected to immunohistochemical staining for Cit H3. The use of these specimens was approved by the Ethics Committee of Tianjin Medical University General Hospital. The results revealed a positive correlation between TNFAIP6 expression and NET formation in human lung adenocarcinoma tissues (Figure 1B). To minimize potential interspecies interactions and exclude the influence of adaptive immunity, a syngeneic tumor (SynT) model was established using the LLC cell line and C57BL/6 nude mice. In this in vivo model, Tnfaip6 expression significantly promoted tumor growth (Figure 1C) and increased NET formation within tumor tissues (Figure 1D). Meanwhile, DNase I treatment significantly abrogated its tumor-promoting effect (Figure S2C).

### 3.2. Secreted TNFAIP6 Promotes NET Formation via SPP1 Rather than Directly

**Figure 2:**
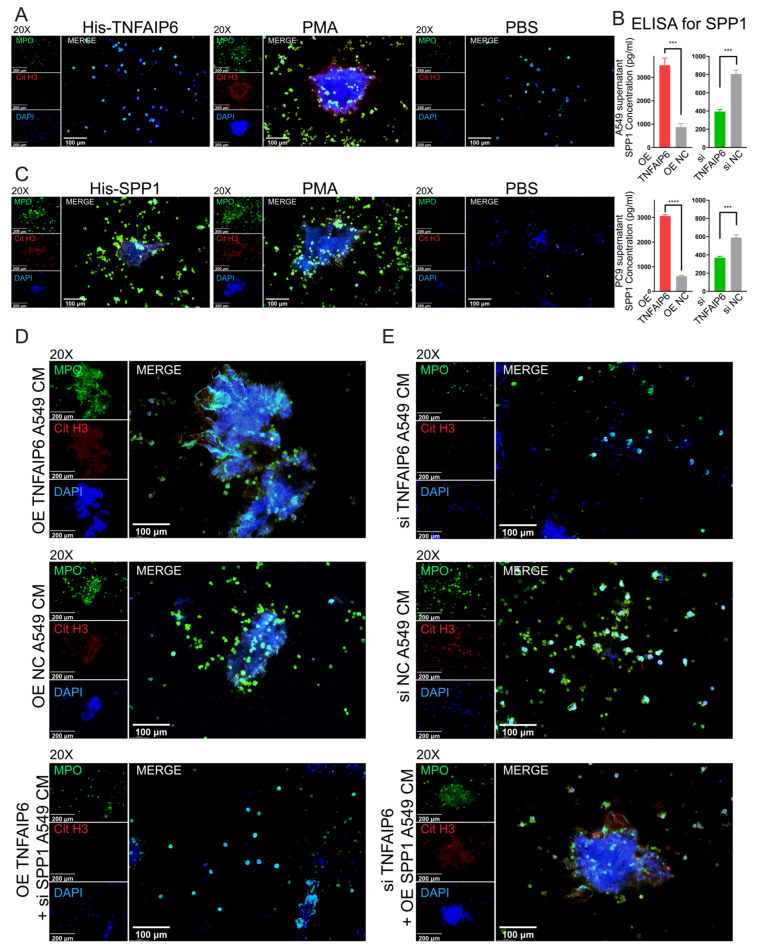
Secreted TNFAIP6 promotes NETosis via SPP1. (**A**) Treatment of neutrophils with recombinant His-tagged TNFAIP6 did not alter myeloperoxidase (MPO, green) or citrullinated histone H3 (Cit H3, red) staining or affect the amount of extracellular DNA, as indicated by DAPI staining (blue). Phosphate-buffered saline (PBS) and phorbol 12-myristate 13-acetate (PMA) were used as negative and positive controls, respectively. (**B**) ELISA analysis of SPP1 levels in culture supernatants demonstrated that TNFAIP6 overexpression increased, whereas TNFAIP6 silencing decreased, SPP1 concentrations in both A549 (top) and PC9 (bottom) cells. The corresponding standard curve is shown in Figure S2E. (**C**) Recombinant His-tagged SPP1 markedly enhanced MPO (green) and Cit H3 (red) staining and increased extracellular DNA release (blue) in neutrophils. (**D**) SPP1 knockdown in TNFAIP6-overexpressing A549 cells abrogated the TNFAIP6-mediated increase in NET marker staining. (**E**) SPP1 overexpression in TNFAIP6-silenced A549 cells rescued NET formation. Similar results were observed in PC9 cells (Figure S3). *** *p* < 0.001; **** *p* < 0.0001.

TNFAIP6 is a secreted protein that has been reported to suppress immune responses in myocardial infarction and tissue injury repair [29,30] and, according to limited evidence, may also contribute to tumorigenesis and cancer progression [31,32]. Given its secretory nature and immunomodulatory functions, TNFAIP6 protein was ectopically expressed, purified under native conditions, and applied directly to neutrophils. This treatment did not induce NET formation, indicating that TNFAIP6 does not directly stimulate NETosis in neutrophils (Figure 2A).

Previous label-free quantitative mass spectrometry (LFQ-MS) analyses suggested that TNFAIP6 increases the concentration of secreted SPP1 in lung adenocarcinoma cell supernatants [19]. This observation was further validated by ELISA in both A549 and PC9 cells (Figure 2B; standard curves are shown in Figure S2E). Immunofluorescence staining demonstrated that recombinant His-tagged SPP1 directly induced NETosis in neutrophils (Figure 2C). Consistently, SPP1 knockdown in TNFAIP6-overexpressing A549 and PC9 cells abrogated the TNFAIP6-mediated enhancement of NET formation (Figure 2D and Figure S3A). Conversely, SPP1 overexpression rescued the inhibitory effects of TNFAIP6 silencing on NETosis in neutrophils co-cultured with A549 and PC9 cells (Figure 2E and Figure S3B).

### 3.3. TNFAIP6 Does Not Alter SPP1 Expression

**Figure 3:**
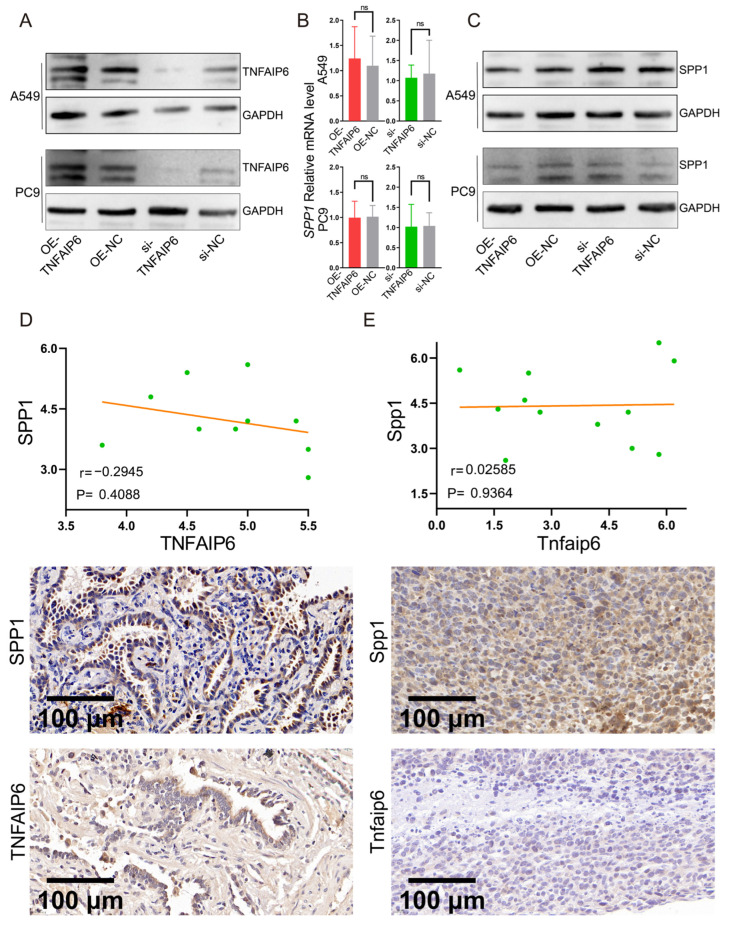
TNFAIP6 does not regulate SPP1 expression in lung adenocarcinoma. (**A**) Western blot analysis confirming the efficiency of *TNFAIP6* overexpression and silencing in A549 and PC9 cells. The uncropped blots are shown in Supplementary Materials S1. (**B**,**C**) Modulation of TNFAIP6 expression in A549 and PC9 cells does not alter SPP1 mRNA (**B**) or protein (**C**) levels. The uncropped blots are shown in Supplementary Materials S1. (**D**) Immunohistochemical staining intensities of TNFAIP6 and SPP1 were not correlated in human lung adenocarcinoma tissues (r = −0.29, *p* = 0.41). (**E**) In vivo, lentiviral Tnfaip6 overexpression in Lewis lung carcinoma (LLC) cells did not alter Spp1 staining intensity in syngeneic tumors (r = 0.03, *p* = 0.94). ns, *p* ≥ 0.05.

A549 and PC9 cells were transiently transfected with *TNFAIP6* overexpression or silencing constructs, and transfection efficiency was confirmed by Western blotting (Figure 3A). Quantitative PCR and Western blot analyses demonstrated that neither SPP1 mRNA nor protein expression was significantly affected by modulation of TNFAIP6 levels (Figure 3B,C). Consistently, SPP1 protein expression did not correlate with TNFAIP6 levels in human lung adenocarcinoma tissue samples (Figure 3D). Moreover, in the SynT model, Tnfaip6 overexpression did not result in increased SPP1 protein expression (Figure 3E).

### 3.4. TNFAIP6 Interacts with CD44 and Affects Extracellular SPP1 Availability

**Figure 4:**
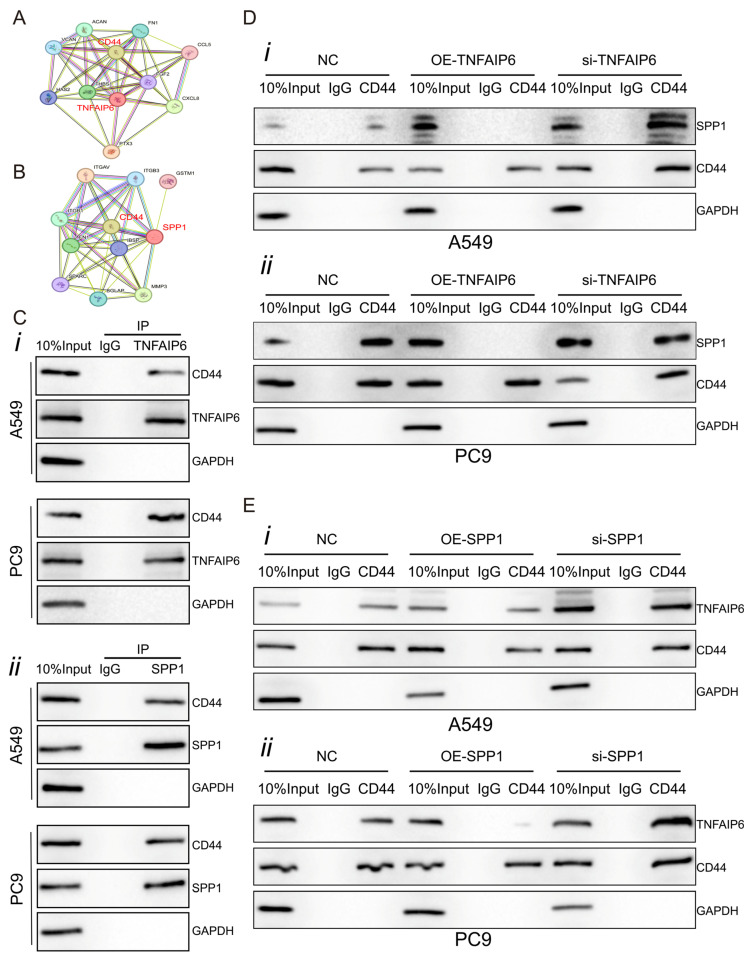
TNFAIP6 associates with CD44 and modulates extracellular SPP1 availability. (**A**,**B**) Protein–protein interaction networks for TNFAIP6 (**A**) and SPP1 (**B**) were predicted using the STRING database [26]. Both TNFAIP6 and SPP1 were predicted to interact with CD44 (highlighted in red). (**C**) Co-immunoprecipitation followed by Western blot analysis confirmed the interactions between TNFAIP6 and CD44 (**i**) and between SPP1 and CD44 (**ii**) in A549 and PC9 cells. The uncropped blots are shown in Supplementary Materials S1. (**D**) TNFAIP6 overexpression in A549 (**i**) and PC9 (**ii**) cells reduced the SPP1–CD44 association, whereas TNFAIP6 silencing enhanced this interaction. The uncropped blots are shown in Supplementary Materials S1. (**E**) Similarly, SPP1 overexpression in A549 (**i**) and PC9 (**ii**) cells diminished the TNFAIP6–CD44 association, whereas SPP1 silencing increased the interaction. The uncropped blots are shown in Supplementary Materials S1.

Based on predictions from the STRING database [26], both TNFAIP6 and SPP1 were predicted to interact with CD44 (Figure 4A,B). These predicted interactions were validated by co-immunoprecipitation followed by Western blot analysis (Figure 4C). Notably, overexpression of TNFAIP6 in A549 and PC9 cells markedly reduced SPP1–CD44 interactions, whereas TNFAIP6 silencing enhanced this interaction (Figure 4D). Consistent results were obtained when SPP1 expression was manipulated in lung adenocarcinoma cells, suggesting that TNFAIP6 and SPP1 mutually influence their association with CD44 (Figure 4E).

### 3.5. NPM1 Upregulates the Transcriptional Activation of TNFAIP6

Previous work demonstrated that TNFAIP6 expression is elevated at both the mRNA and protein levels in lung cancer tissues [19]. Consistent with these findings, TNFAIP6 expression was significantly increased at the mRNA and protein levels in A549 and PC9 cells compared with BEAS-2B cells (Figure S4A,B). No significant differences were observed in TNFAIP6 mRNA degradation rates (Figure S4C) or protein stability (Figure S4D) among A549, PC9, and BEAS-2B cells. Notably, dual-luciferase reporter assays demonstrated enhanced transcriptional activation of the *TNFAIP6* promoter in lung adenocarcinoma cells, indicating transcriptional upregulation of *TNFAIP6* (Figure S4E).

**Figure 5:**
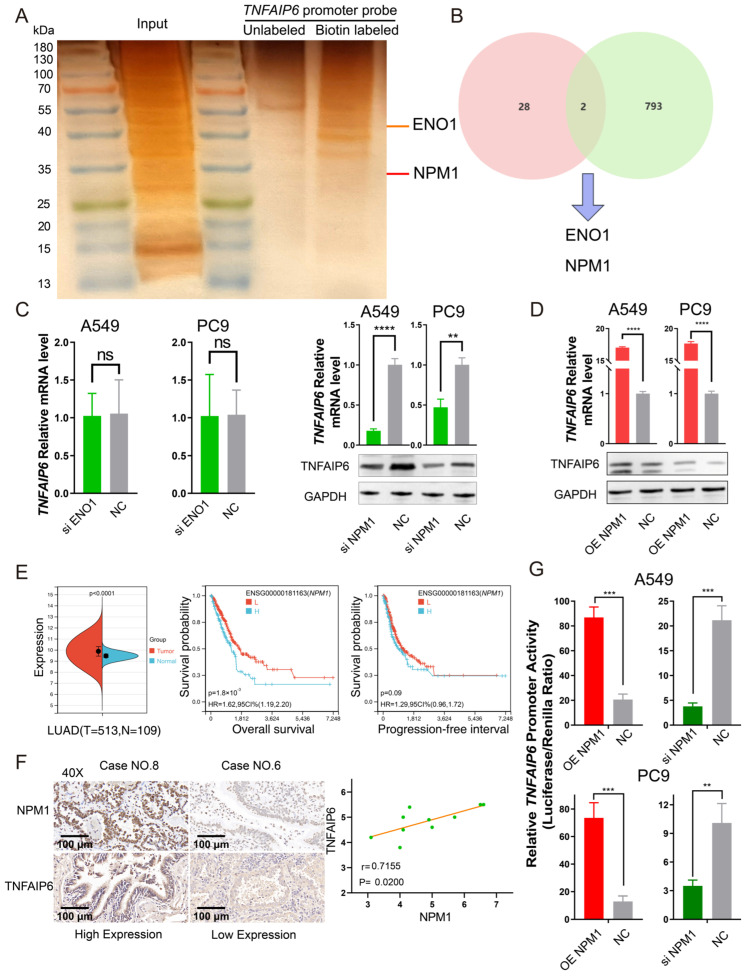
NPM1 upregulates TNFAIP6 expression by promoting its transcriptional activation in lung adenocarcinoma. (**A**) Silver staining of DNA pulldown assay products. Based on evidence of transcriptional upregulation of *TNFAIP6* in lung adenocarcinoma cells (Figure S4), biotin-labeled DNA probes spanning the −2000 to +75 bp region of the *TNFAIP6* promoter were generated. DNA pulldown assays were performed, and the captured proteins were visualized by silver staining and identified by mass spectrometry, as summarized in Supplementary Dataset S1. (**B**) Venn diagram illustrating the screening of transcriptional regulators. The top 30 proteins identified by abundance in the mass spectrometry analysis (light pink circle) were intersected with 795 curated transcription factors obtained from the TRRUST database [25]. NPM1 and ENO1 emerged as candidate transcriptional regulators of TNFAIP6. (**C**) NPM1 knockdown in A549 and PC9 cells significantly reduced TNFAIP6 mRNA and protein (**right panel**) expression, whereas ENO1 silencing did not alter *TNFAIP6* mRNA levels (**left panel**). The uncropped blots are shown in Supplementary Materials S1. (**D**) NPM1 overexpression increased TNFAIP6 mRNA (top) and protein (bottom) expression. The uncropped blots are shown in Supplementary Materials S1. (**E**) Bioinformatic analyses of TCGA data demonstrated that NPM1 is significantly overexpressed in lung adenocarcinoma (LUAD) tissues (red) compared with normal lung tissues (*p* < 0.01; **left panel**). Elevated *NPM1* expression (cyan line) was associated with reduced overall survival (*p* = 0.0018; **middle panel**) and a trend toward a shorter progression-free interval (*p* = 0.09; **right panel**). (**F**) Immunohistochemical analysis revealed a positive correlation between NPM1 and TNFAIP6 protein expression in lung adenocarcinoma tissues (r = 0.72, *p* = 0.02). (**G**) Dual-luciferase reporter assays showed that NPM1 overexpression increased, whereas NPM1 silencing decreased, *TNFAIP6* promoter-driven luciferase activity in A549 and PC9 cells. A schematic of the reporter constructs is shown in Figure S4E. ns, *p* ≥ 0.05; ** *p* < 0.01; *** *p* < 0.001; **** *p* < 0.0001.

Given these observations, *TNFAIP6* promoter probes were constructed and a DNA pulldown assay coupled with mass spectrometry was performed; silver staining of the pulldown products is shown in Figure 5A. NPM1 was identified as a candidate regulator binding to the *TNFAIP6* promoter by intersecting the mass spectrometry results with known transcription factors curated in the TRRUST database [25] (Figure 5B). Quantitative PCR and Western blot analyses further confirmed that NPM1 upregulates TNFAIP6 expression at both the mRNA and protein levels (Figure 5C,D).

Consistent with these findings, bioinformatic analyses based on TCGA demonstrated that *NPM1* is overexpressed in lung adenocarcinoma tissues and is associated with poorer patient prognosis (Figure 5E). At the protein level, NPM1 expression was positively correlated with TNFAIP6 expression in lung adenocarcinoma tissues (Figure 5F). Moreover, overexpression or silencing of NPM1 in A549 and PC9 cells significantly increased or decreased, respectively, the luciferase activity driven by the *TNFAIP6* promoter (Figure 5G).

### 3.6. NPM1 Interacts with the −2000 to −1700 bp Region of the TNFAIP6 Promoter

**Figure 6:**
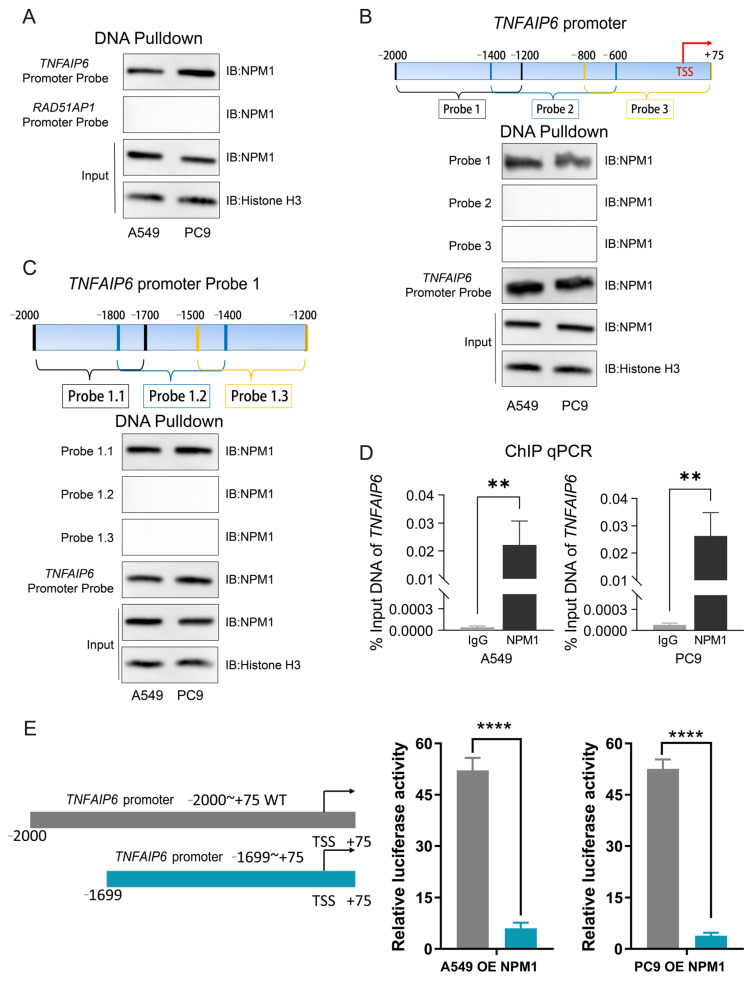
NPM1 associates with the −2000 to −1700 bp region of the *TNFAIP6* promoter. (**A**) DNA pulldown assays confirmed the association of NPM1 with the *TNFAIP6* promoter probe. A RAD51AP1 promoter probe was used as a negative control. The uncropped blots are shown in Supplementary Materials S1. (**B**) Mapping of the NPM1-interacting region within the *TNFAIP6* promoter. The promoter was divided into three fragments (−2000 to −1200, −1400 to −600, and −800 to +75 bp), and corresponding biotinylated DNA probes were synthesized (schematic shown in the upper panel). DNA pulldown followed by Western blot analysis demonstrated that Probe 1 (−2000 to −1200 bp) interacted with NPM1 (lower panel). The uncropped blots are shown in Supplementary Materials S1. (**C**) Fine mapping of the NPM1-interacting site. Probe 1 was further subdivided into three fragments (schematic shown in the upper panel), revealing specific association of NPM1 with the −2000 to −1700 bp region of the *TNFAIP6* promoter (lower panel). The uncropped blots are shown in Supplementary Materials S1. (**D**) Chromatin immunoprecipitation followed by quantitative PCR demonstrated significantly greater enrichment of the −2000 to −1700 bp region of the *TNFAIP6* promoter in NPM1 immunoprecipitates compared with IgG controls in both A549 and PC9 cells. (**E**) Dual-luciferase reporter assays in NPM1-overexpressing cells showed that deletion of the −2000 to −1700 bp region (−1699 to +75 bp, blue bar) significantly reduced luciferase activity compared with the full-length promoter construct (−2000 to +75 bp, wild-type, gray bar). Schematic representations of the reporter constructs are shown in the left panel. ** *p* < 0.01; **** *p* < 0.0001.

The association of NPM1 with the *TNFAIP6* promoter in A549 and PC9 cells was first confirmed by DNA pulldown followed by Western blot analysis (Figure 6A). To define the region of interaction, the *TNFAIP6* promoter was divided into three fragments, revealing NPM1 association within the −2000 to −1200 bp region (Figure 6B). This region was further subdivided, and the NPM1 interaction was localized to the −2000 to −1700 bp segment (Figure 6C). Consistently, chromatin immunoprecipitation followed by quantitative PCR demonstrated significant enrichment of NPM1 at the −2000 to −1700 bp region of the *TNFAIP6* promoter (Figure 6D). Dual-luciferase reporter assays further confirmed that NPM1-mediated transcriptional activation of *TNFAIP6* depends on this −2000 to −1700 bp promoter region (Figure 6E).

## 4. Discussion

**Figure 7:**
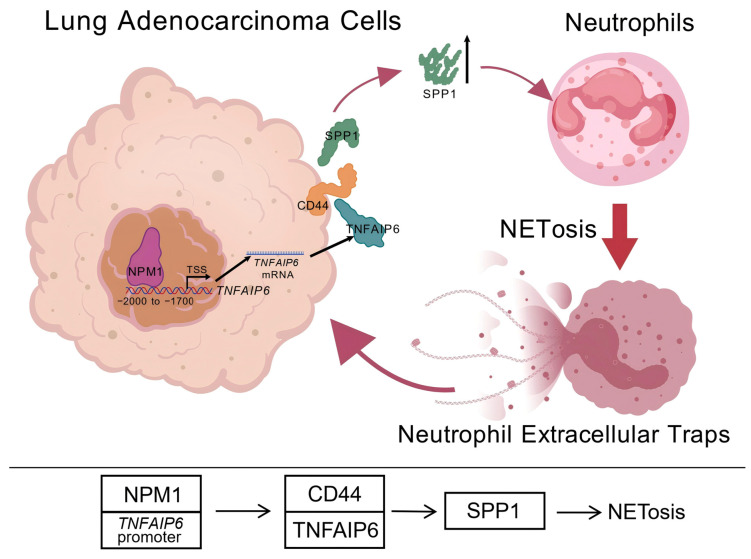
Schematic of the NPM1–TNFAIP6–CD44–SPP1 axis in lung adenocarcinoma-induced NET formation. In lung adenocarcinoma cells, elevated levels of TNFAIP6 interact with CD44, leading to increased extracellular availability of SPP1, which stimulates NETosis; NPM1 upregulates the transcriptional activation of *TNFAIP6* by interacting with its −2000 to −1700 bp promoter region.

The present study demonstrates that lung adenocarcinoma cells directly promote NET formation by upregulating TNFAIP6 (Figure 1). Mechanistically, TNFAIP6 interacts with CD44 in lung adenocarcinoma cells, leading to increased extracellular levels of SPP1 (Figure 3 and Figure 4). SPP1 subsequently induces NET formation in neutrophils (Figure 2B–E), rather than TNFAIP6 directly triggering NETosis (Figure 2A). Upstream analyses further clarified the mechanism underlying TNFAIP6 overexpression in lung adenocarcinoma, attributing it to transcriptional activation (Figure S4). Specifically, NPM1 was shown to enhance *TNFAIP6* transcription by interacting with the −2000 to −1700 bp region of its promoter (Figure 5 and Figure 6). A schematic summary of the NPM1–TNFAIP6–CD44–SPP1 axis uncovered in this study is presented in Figure 7.

NETs were first described in 2004 by Volker Brinkmann as neutrophil-derived reticular structures that trap and eliminate invading microorganisms [33]. Increasing evidence has since implicated NETs in cancer progression across multiple malignancies, including lung adenocarcinoma [34,35,36,37,38]. Despite these observations, the mechanisms governing NET formation within tumors remain incompletely understood. Tumor cells have been reported to directly induce NETosis in neutrophils. For example, melanoma and colon carcinoma cells secrete CXCR1 and CXCR2 agonists, particularly IL-8, to stimulate NET formation [18]. Consistent with these findings, our results suggested that lung adenocarcinoma cells directly induce NETosis within the TIME. Notably, this work provides evidence that this process is dependent on TNFAIP6 upregulation.

The role of TNFAIP6 within the TIME appears to be multifaceted. Previous work showed that TNFAIP6 upregulation in lung adenocarcinoma markedly inhibits early neutrophil apoptosis, as silencing TNFAIP6 in A549 cells increased the proportion of neutrophils in the Annexin V-positive, propidium iodide-negative quadrant [19]. In contrast, the current study demonstrates a NETosis-promoting role for TNFAIP6 in lung adenocarcinoma (Figure 1 and Figure 2). Rather than being contradictory, these findings underscore the complex regulatory functions of TNFAIP6 in tumor-associated neutrophils. TNFAIP6 may simultaneously promote neutrophil survival, either directly or through indirect mechanisms, while interacting with CD44 to enhance the extracellular availability of SPP1 and thereby induce NETosis. Together, these actions finely tune neutrophil responses and reshape the TIME. It should be noted that the present study focuses specifically on the role of TNFAIP6 in NETosis, and the broader regulatory network governing its functions in the tumor context warrants further investigation.

Meanwhile, the NETosis-promoting effects of TNFAIP6 were found to be dependent on extracellular SPP1 (Figure 2). Consistent with the report by Shen et al., SPP1 directly induced NET formation in neutrophils (Figure 2C) [39]. Notably, TNFAIP6 did not regulate SPP1 expression (Figure 3); instead, it increased the extracellular availability of SPP1 by interacting with CD44 in lung adenocarcinoma cells (Figure 4). These findings suggest a potential competitive relationship among TNFAIP6, CD44, and SPP1. Competitive protein–protein interactions are common in diverse biological processes [40], and the present results raise the possibility that such a mechanism underlies the TNFAIP6–CD44–SPP1 axis. However, whether TNFAIP6 mediates this effect through steric hindrance or induces conformational changes upon interactions remains unclear. Furthermore, it is unknown whether other proteins or molecules participate in this process, and whether the elevation in extracellular SPP1 results from enhanced secretion, membrane dissociation, or increased protein stability following TNFAIP6–CD44 interaction. These questions warrant further investigation.

With respect to upstream regulation, we found that NPM1 interacted with the −2000 to −1700 bp region of the *TNFAIP6* promoter (Figure 6). The role of NPM1 as a transcriptional regulator in lung adenocarcinoma has rarely been reported [41]. Moreover, this interaction region is distal to the transcription start site (TSS) and lacks canonical promoter elements, such as TATA, GC, or CAAT boxes, making it difficult to classify NPM1 as a classical transcription factor in this context. Instead, NPM1 is more commonly recognized as a transcriptional co-regulator. For instance, in glioblastoma, NPM1 forms a complex with RUNX1 to bind upstream of a cis-regulatory element of *FOSL2*, thereby maintaining chromatin accessibility and promoting transcription [42]. Similarly, the present study identified NPM1 binding at a moderately distal site relative to the TSS, suggesting that *TNFAIP6* transcription may be enhanced through an analogous co-regulatory mechanism.

In addition, the −2000 to −1700 bp region of the *TNFAIP6* promoter contains guanine-rich sequences (e.g., 5′-TTGGGAGAGGGGAGAAAGAA-3′) that may form G-quadruplex structures upon DNA unwinding. As reviewed by Varshney et al., G-quadruplexes play critical roles in transcriptional regulation [43]. For example, their stable formation has been shown to repress c-MYC transcription [44]. Numerous factors modulate the dynamic equilibrium of G-quadruplex structures in eukaryotic gene regulation [45]. Thus, NPM1 may promote *TNFAIP6* transcription by directly or indirectly influencing G-quadruplex formation within this promoter region.

The present study has several limitations. First, while our Co-IP data suggested an association between TNFAIP6 and CD44, these experiments did not establish direct competition or displacement of SPP1. Moreover, whether the TNFAIP6–CD44 interaction increases extracellular SPP1 via enhanced secretion or other mechanisms remains to be determined. Second, although NPM1 was identified as a transcriptional regulator of *TNFAIP6*, the present study did not systematically screen for other transcription factors or regulatory elements that may also contribute to TNFAIP6 expression in lung adenocarcinoma. It therefore remains unclear whether NPM1 acts independently or cooperatively with other factors to modulate *TNFAIP6* transcription. Third, the human tissue analyses were based on a relatively small cohort (*n* = 10), which limits statistical power and the generalizability of conclusions regarding clinical relevance. Validation in larger, independent patient cohorts is warranted. Fourth, while this study provides mechanistic insights using in vitro and in vivo models, the heterogeneity of patient tumors and the tumor microenvironment may influence the extent to which the NPM1–TNFAIP6–CD44–SPP1 axis is activated. Further validation using patient-derived xenografts or additional orthotopic models would help to confirm the translational relevance of these findings. Addressing these questions in future studies will contribute to a more comprehensive understanding of how TNFAIP6-mediated NETosis regulates tumor progression.

## 5. Conclusions

This study suggests that NPM1 upregulates the transcriptional activation of *TNFAIP6* in lung adenocarcinoma cells. TNFAIP6 then interacts with CD44 in these cells, leading to increased extracellular availability of SPP1 and subsequent induction of NETosis. Specifically, NPM1 interacts with the −2000 to −1700 bp region of the *TNFAIP6* promoter relative to the TSS. Despite certain limitations, these findings establish a novel conceptual and mechanistic framework for the NPM1–TNFAIP6–CD44–SPP1 axis in promoting NET formation within the TIME. Therapeutic disruption of the interaction of NPM1 with the *TNFAIP6* promoter, such as via small-molecule inhibitors, and/or interference with TNFAIP6–CD44 interactions via blocking antibodies, may attenuate tumor-induced NETosis and thereby suppress tumor progression. These findings may provide a conceptual basis for exploring whether targeting this axis could have therapeutic relevance, although further studies are needed to validate this possibility.

## Data Availability

The data presented in this study are available on request from the corresponding author.

## Supplementary Materials

The following supporting information can be downloaded at https://www.mdpi.com/article/10.3390/cancers18061023/s1, Supplementary Materials S1: Original images of western blot. Dataset S1: MS results. Figure S1: TNFAIP6 may influence NETosis in lung adenocarcinoma; Figure S2: Supplementary data for Figure 1 and Figure 2; Figure S3: TNFAIP6 in PC9 cells promotes NETosis via SPP1; Figure S4: *TNFAIP6* is transcriptionally upregulated in lung adenocarcinoma cells; Table S1: Primers used in this study.

## Abbreviations

The following abbreviations are used in this manuscript:

NETs	Neutrophil extracellular traps
TIME	Tumor immune microenvironment
TNFAIP6	Tumor Necrosis Factor Alpha Inducible Protein 6
SPP1	Secreted Phosphoprotein 1
NPM1	Nucleophosmin 1
Cit H3	Citrullinated Histone H3
MPO	Myeloperoxidase
IL-18	Interleukin 18
IL-1β	Interleukin 1 Beta
CHX	Cycloheximide
LUAD	Lung adenocarcinoma
RUNX1	Runt-Related Transcription Factor 1
FOSL2	FOS-Like Antigen 2
ENO1	Enolase 1
CXCR1	C-X-C Chemokine Receptor Type 1
CXCR2	C-X-C Chemokine Receptor Type 2
