# Supplementary material for: Lung Adenocarcinoma Promotes NETosis via the NPM1–TNFAIP6–CD44–SPP1 Axis
Source: Cancers (Basel). 2026 Mar 22;18(6):1023. doi: 10.3390/cancers18061023 (PMC13026014; doi:10.3390/cancers18061023)
Supplement: Supplementary file 1 [file cancers-18-01023-s001.zip › Supplementary Materials Figures and Table.pdf]

Supplementary Figures

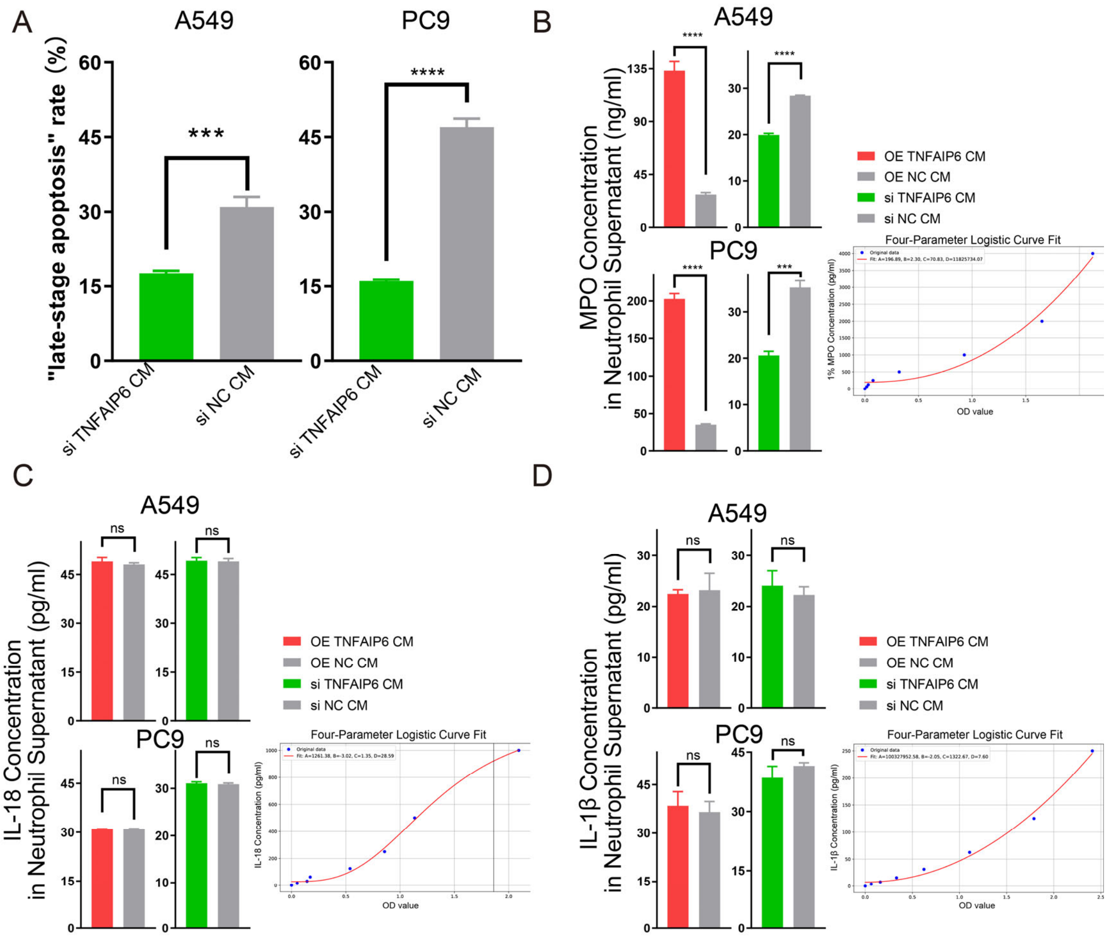

Figure S1. TNFAIP6 may influence NETosis in lung adenocarcinoma. **(A)** In a previous study, TNFAIP6 was silenced in A549 and PC9 cells, and conditioned media (CM) were collected, diluted, and used to treat neutrophils. Annexin V–propidium iodide (AV–PI) analysis demonstrated that CM derived from TNFAIP6-silenced cells significantly reduced the proportion of neutrophils in the Q2 quadrant [19]. **(B–D)** Enzyme-linked immunosorbent assays were performed to quantify myeloperoxidase (MPO), interleukin-18 (IL-18), and interleukin-1β (IL-1β) concentrations in the supernatants of CM-treated neutrophils. TNFAIP6 expression in both A549 and PC9 cells significantly increased MPO levels (B), whereas no significant changes were observed in IL-18 (C) or IL-1β (D). Standard curves were generated using a four-parameter logistic regression model and are shown in the lower right panels of (B–D). ns, $P \geq 0.05$; ***$P < 0.001$; ****$P < 0.0001$.

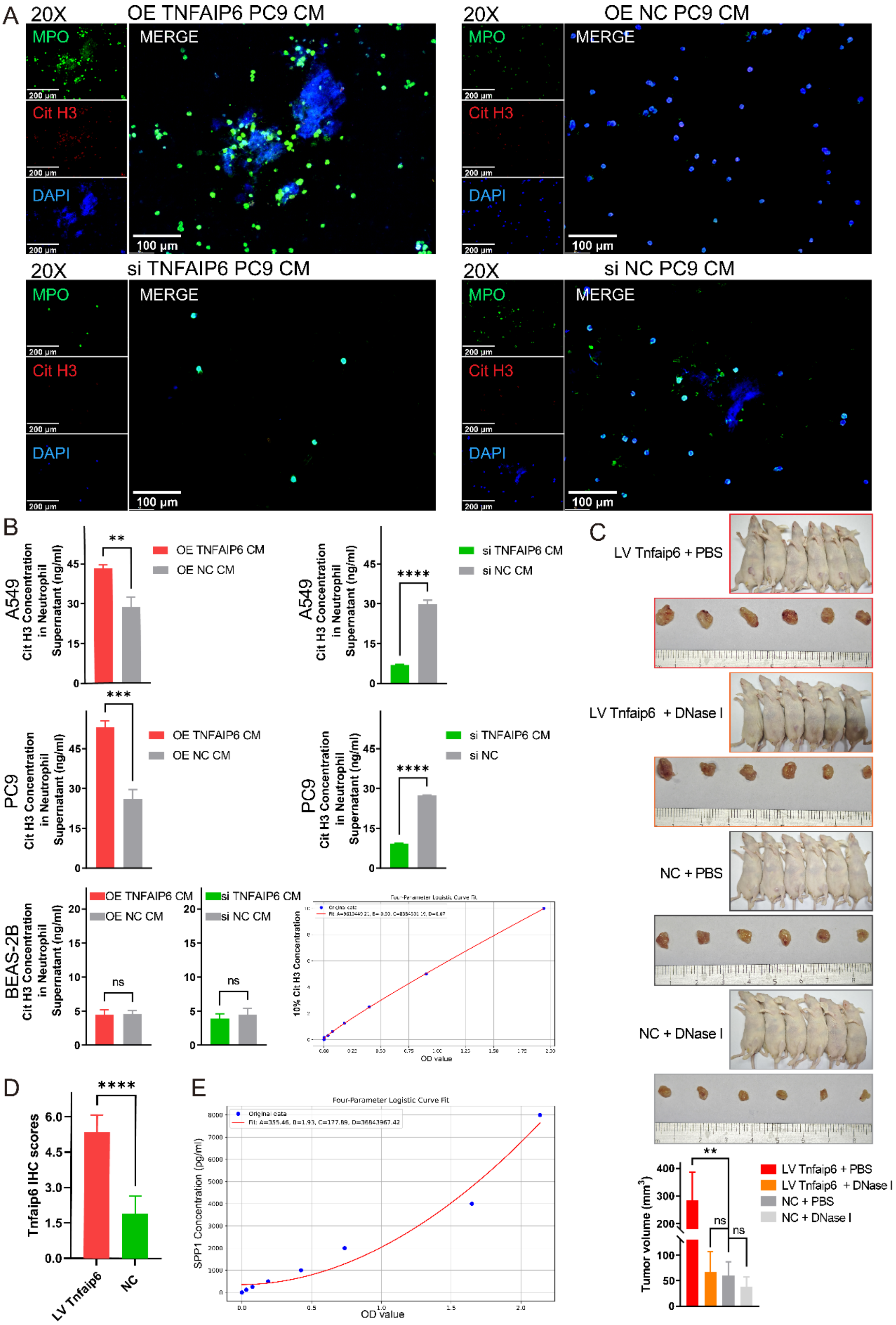

Figure S2. Supplementary data for Figures 1 and 2. **(A)** TNFAIP6 overexpression in PC9 cells enhances myeloperoxidase (MPO, green), citrullinated histone H3 (Cit H3, red), and extracellular DNA (blue) staining, indicating increased NET formation. **(B)** The Cit H3 concentration was significantly increased in neutrophil supernatants when the cells were treated with CM from TNFAIP6-overexpressing A549 and PC9 cells and decreased when they were treated with CM from TNFAIP6-knockdown A549 and PC9 cells

($P < 0.05$). No alterations in Cit H3 concentration were observed when the cells were treated with CM from BEAS-2B cells with either TNFAIP6 overexpression or knockdown ($P > 0.05$). **(C)** Deoxyribonuclease I (DNase I) was used to block NET formation in vivo. The tumor volumes in the LV Tnfaip6+DNase I group were similar to those in the NC+PBS group ($P > 0.05$). **(D)** Immunohistochemical staining confirms the successful lentiviral transduction and stable expression of Tnfaip6 in Lewis lung carcinoma (LLC) cells and in syngeneic tumor (SynT) tissues. **(E)** The standard curve for the SPP1 ELISA kit was generated using a four-parameter logistic regression model implemented in Python 3.13.2.

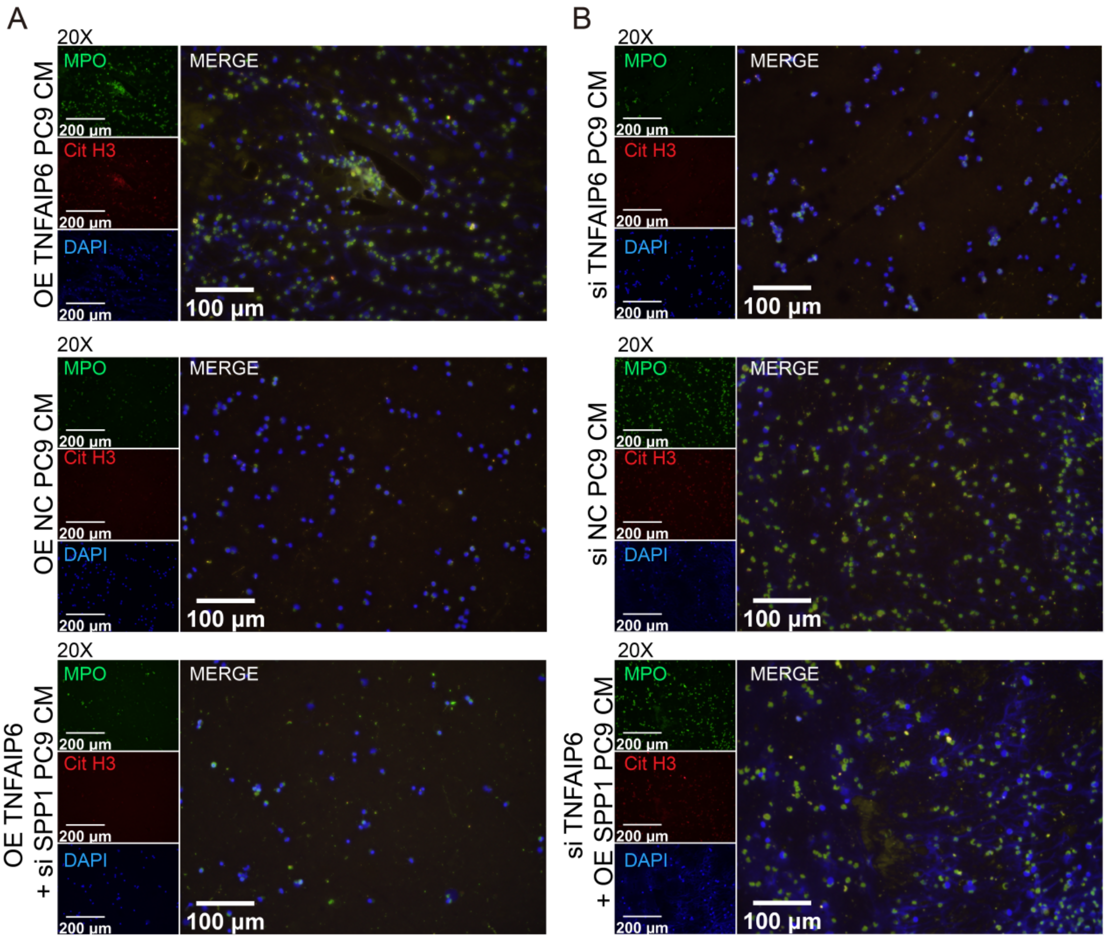

Figure S3. TNFAIP6 in PC9 cells promotes NETosis via SPP1. **(A)** Silencing of SPP1 abrogated the TNFAIP6 overexpression-induced increases in myeloperoxidase (MPO, green), citrullinated histone H3 (Cit H3, red), and extracellular DNA (blue) staining in neutrophils treated with conditioned medium from TNFAIP6-overexpressing PC9 cells. **(B)** Conversely, SPP1 overexpression rescued the inhibitory effects of TNFAIP6 silencing on NET formation.

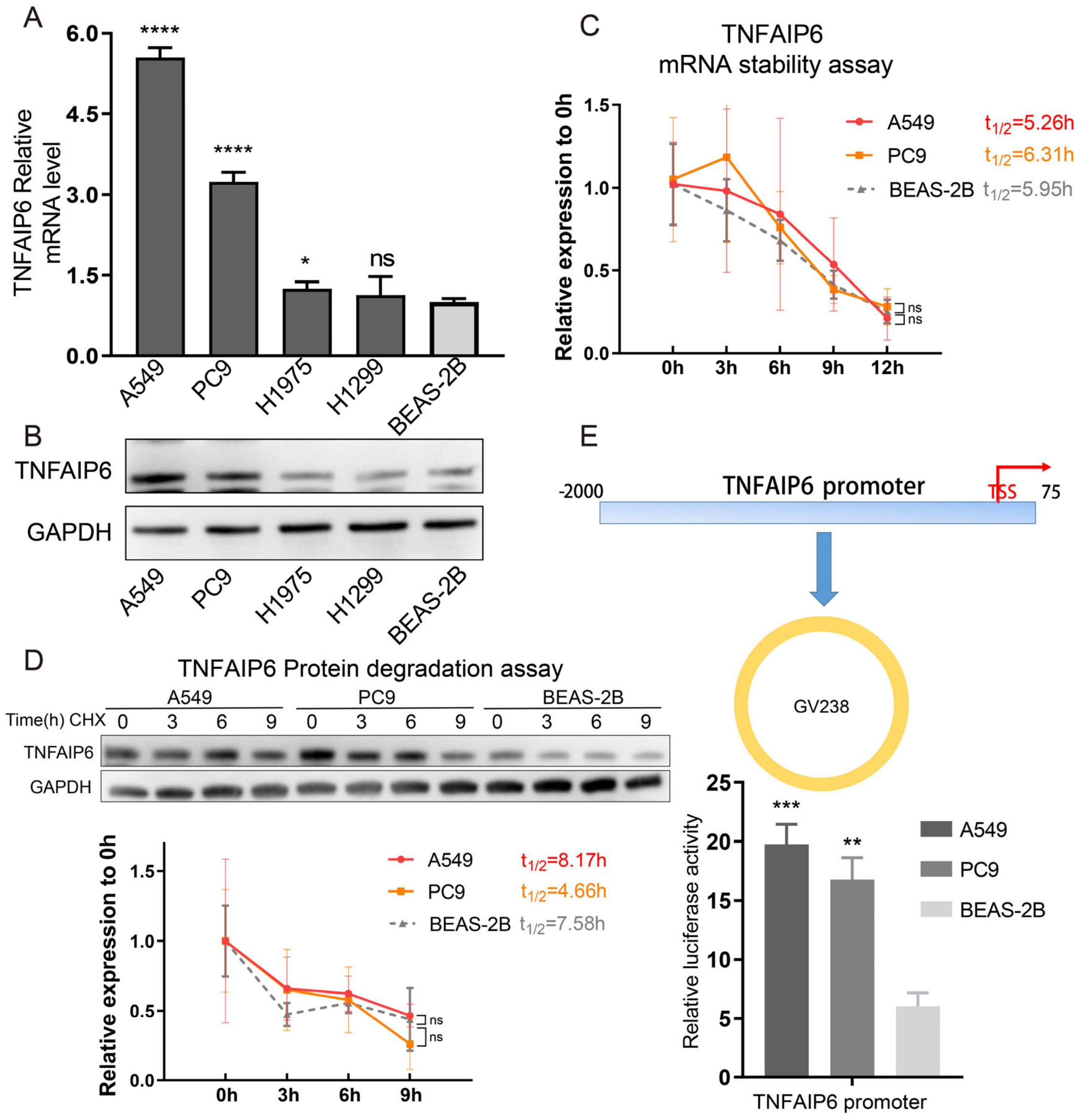

Figure S4. TNFAIP6 is transcriptionally upregulated in lung adenocarcinoma cells. **(A and B)** TNFAIP6 mRNA (A) and protein (B) expression levels were significantly higher in A549 and PC9 cells than in BEAS-2B human normal lung epithelial cells. **(C)** RNA stability assays showed no significant differences in Tnfaip6 mRNA half-life among A549 (red line, t1/2 = 5.26 h), PC9 (orange line, t1/2 = 6.31 h), and BEAS-2B cells (gray line, t1/2 = 5.95 h) following treatment with actinomycin D. **(D)** Protein degradation assays demonstrated no significant differences in TNFAIP6 protein half-life among A549 (red line, t1/2 = 5.26 h), PC9 (orange line, t1/2 = 6.31 h), and BEAS-2B cells (gray line, t1/2 = 5.95 h). Cycloheximide was used to inhibit de novo protein synthesis. **(E)** Dual-luciferase reporter assays showed that the TNFAIP6 promoter region (−2000 to +75 bp), cloned into the GV238 vector (schematic shown in the top panel), exhibited significantly higher relative luciferase activity in A549 and PC9 cells than in BEAS-2B cells (bottom panel). ns, $P \geq 0.05$; *$P < 0.05$; **$P < 0.01$; ***$P < 0.001$; ****$P < 0.0001$.

Supplementary Table

Table S1. Primers used in this study

Target genes	Primers (5'-3')
ACTIN-F	TCGTGCGTGACATTAAGGAGAAGC
ACTIN-R	CAGGAAGGAAGGCTGGAAGAGTG
TNFAIP6-F	TCACCTACGCAGAAGCTAAGGC
TNFAIP6-R	TCCAACTCTGCCCTTAGCCATC
SPP1-F	GAAGTTTCGCAGACCTGACAT
SPP1-R	GTATGCACCATTCAACTCCTCG
TNFAIP6 -2000/+75 F (biotin-labeled)	Biotin-AACCCCATTTCCTCTTTCC
TNFAIP6 -2000/+75 F (unlabeled)	AACCCCATTTCCTCTTTCC
TNFAIP6 -2000/+75 R	ATCGTCAGTTGTAGTGAAG
RAD51AP1 -2000/+50 F	Biotin- AAATCAGTTCTTACATTATTTTCTACACTG
RAD51AP1 -2000/+50 R	GGTCCCTTTCAAGGCTTGG
TNFAIP6 -2000/-1200 F	Biotin-AACCCCATTTCCTCTTTCCT
TNFAIP6 -2000/-1200 R	AGAGTACAGAATTTATTGG
TNFAIP6 -1400/-600 F	Biotin-ATGCCTGTCTTCTCCATAGT
TNFAIP6 -1400/-600 R	CGTCAGGATGTTCCAGCAG
TNFAIP6 -800/+75 F	Biotin-GACATGATGACTTTACTGTA
TNFAIP6 -800/+75 R	ATCGTCAGTTGTAGTGAAG
TNFAIP6 -2000/-1700 F	Biotin-AACCCCATTTCCTCTTTCCT
TNFAIP6 -2000/-1700 R	AGATACCCTTCTGTGTTCAT
TNFAIP6 -1800/-1400 F	Biotin-AAAGCAACTCATTAAATTTT
TNFAIP6 -1800/-1400 R	GGTTACTGAGAGCAAGATTC
TNFAIP6 -1500/-1200 F	Biotin-TCCTAAACCACAAACTTAAA
TNFAIP6 -1500/-1200 R	AGAGTACAGAATTTATTGGA
TNFAIP6 -2000/-1700 F (ChIP)	TGAGAGTTGGGAGAGGGGAG
TNFAIP6 -2000/-1700 R (ChIP)	CTCCAAGTAGAGCGAAGTGTGA
