# Supplementary material for: Lung Adenocarcinoma Promotes NETosis via the NPM1–TNFAIP6–CD44–SPP1 Axis
Source: Cancers (Basel). 2026 Mar 22;18(6):1023. doi: 10.3390/cancers18061023 (PMC13026014; doi:10.3390/cancers18061023)
Supplement: Supplementary file 1 [file cancers-18-01023-s001.zip › Supplementary Materials S1/Original images of uncropped WB membranes and silver-stained gels.pdf]

Fig 3A A549 IB TNFAIP6

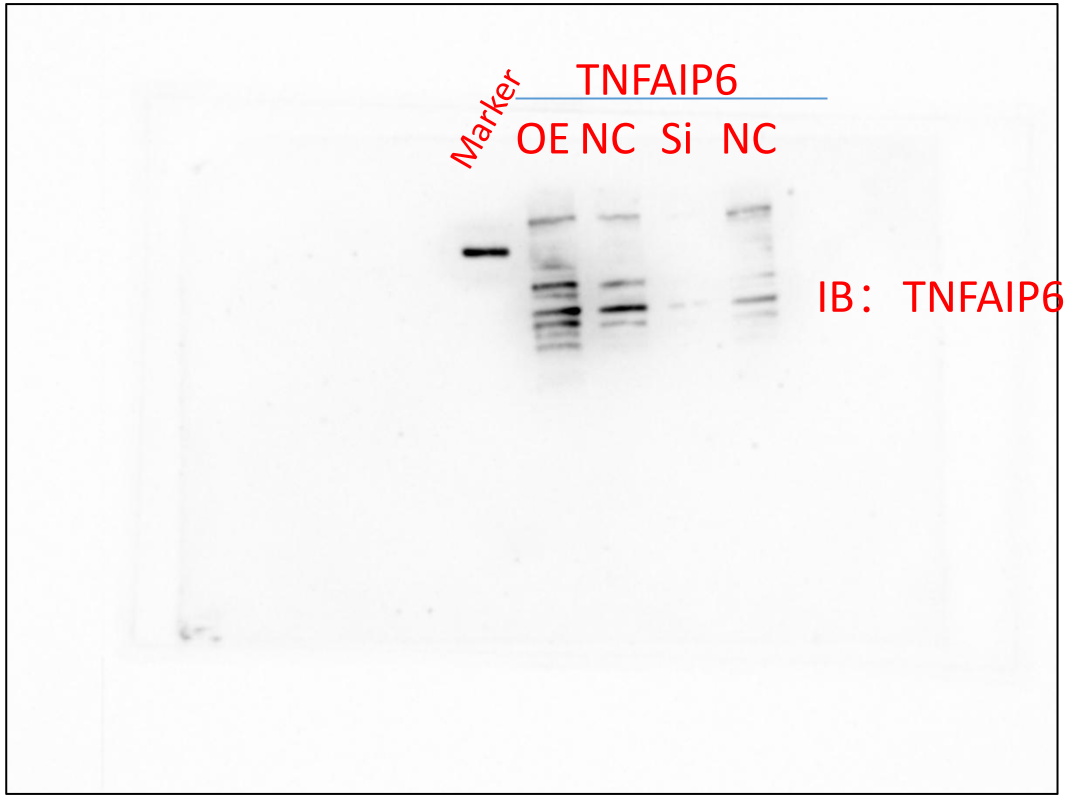

Without marker

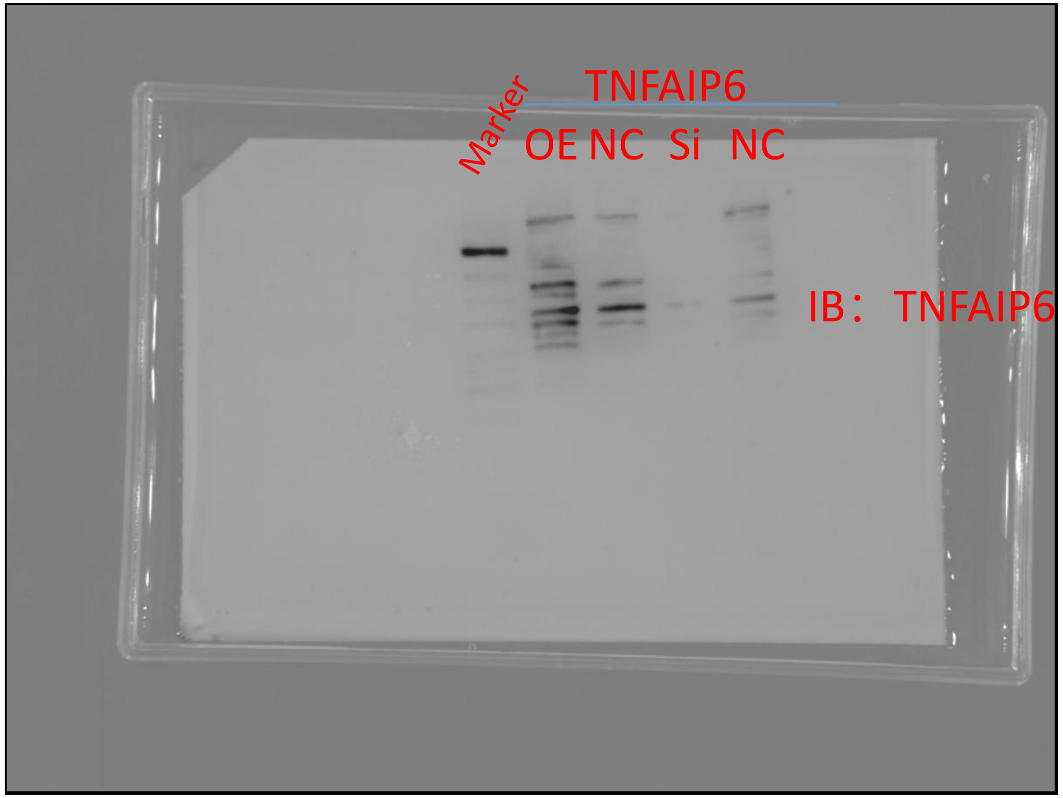

With marker

Fig 3A A549 IB GAPDH

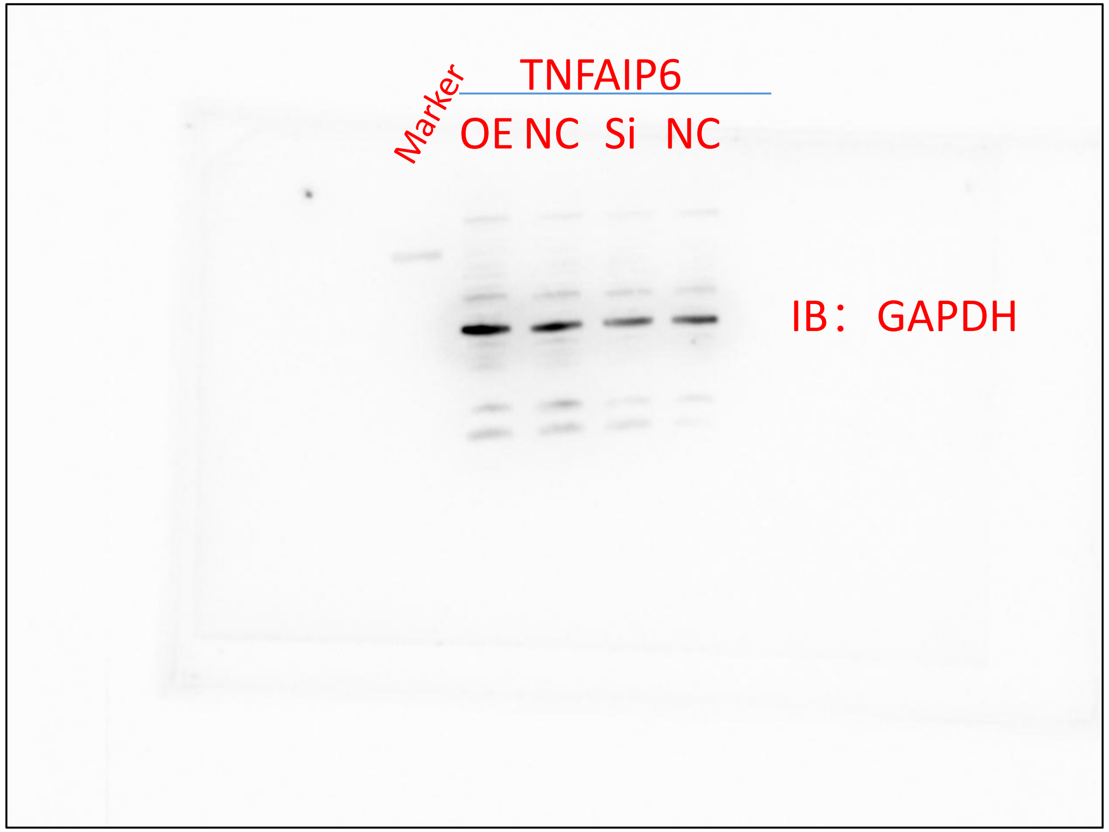

Without marker

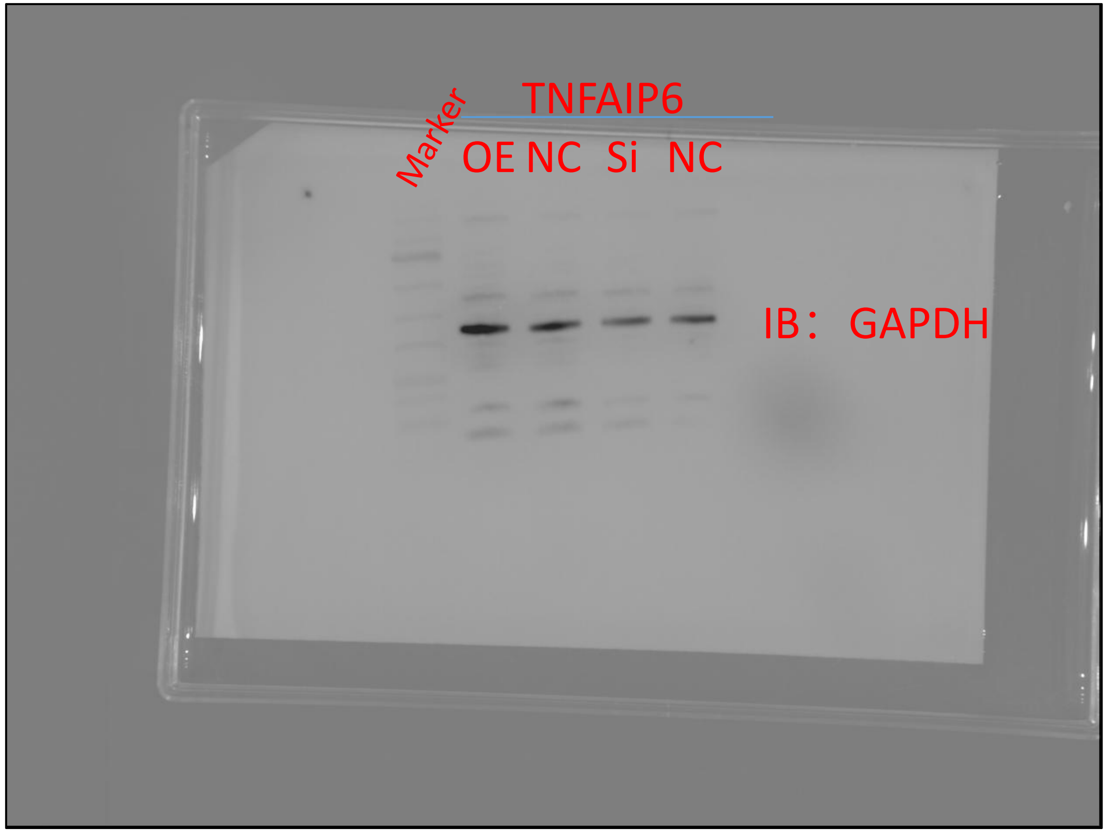

With marker

Fig 3A PC9 IB TNFAIP6

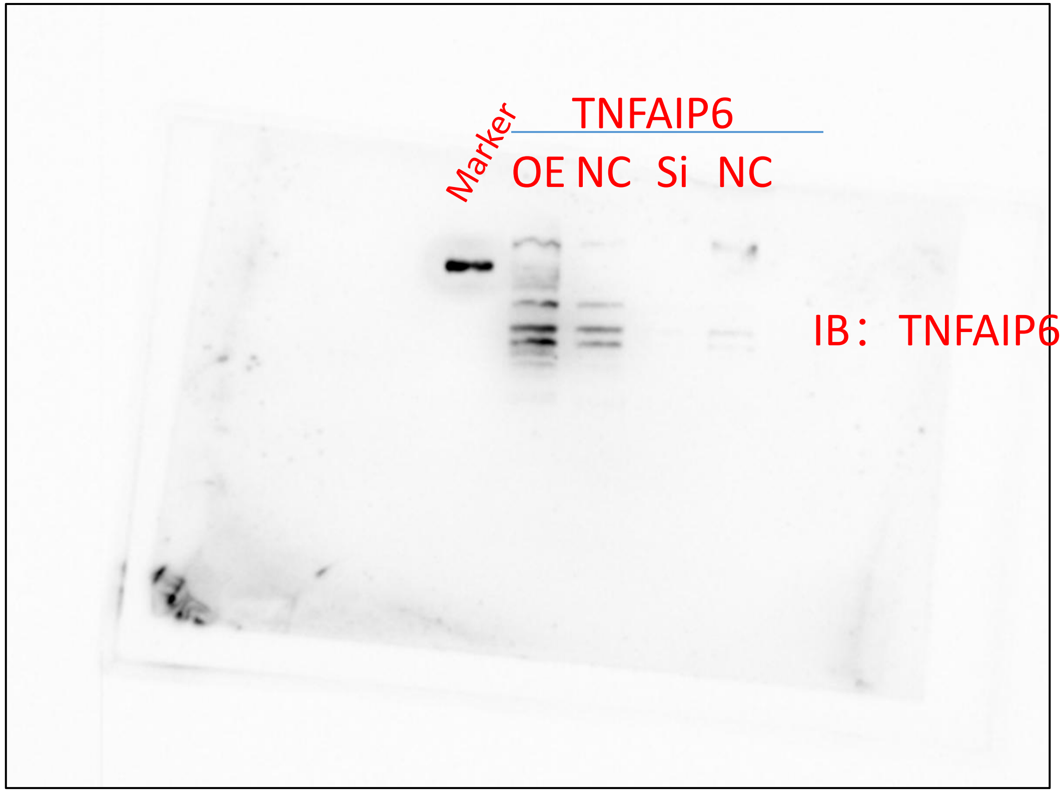

Without marker

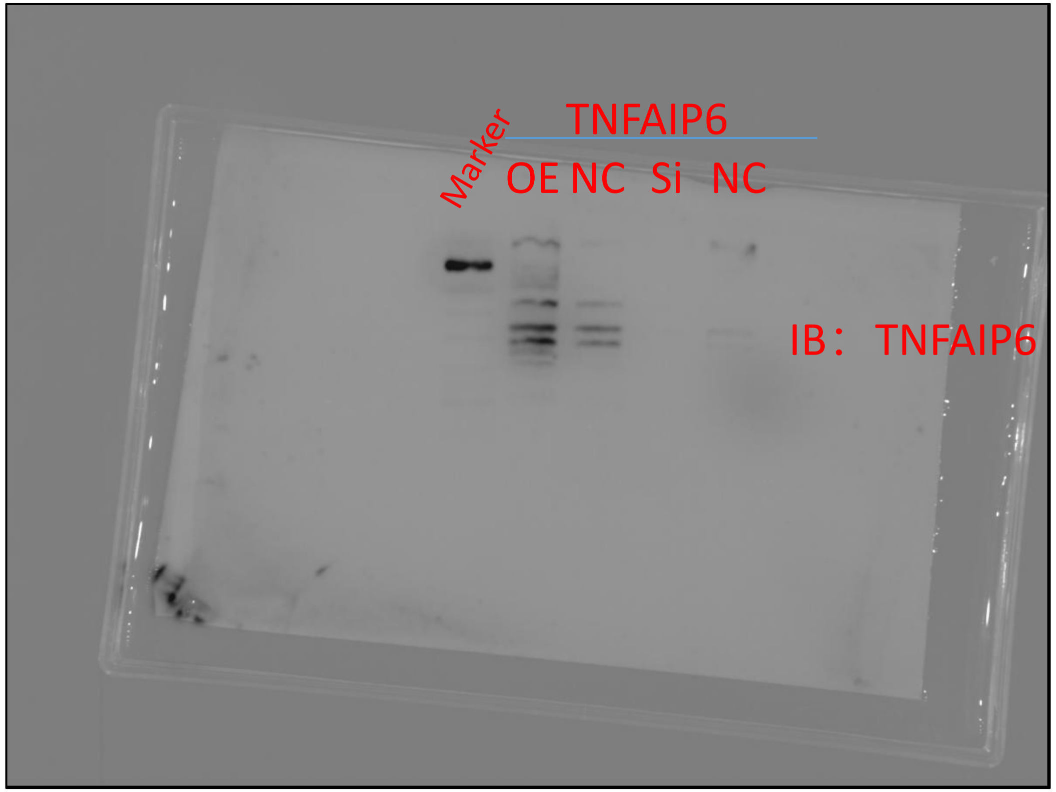

With marker

Fig 3A PC9 IB GAPDH

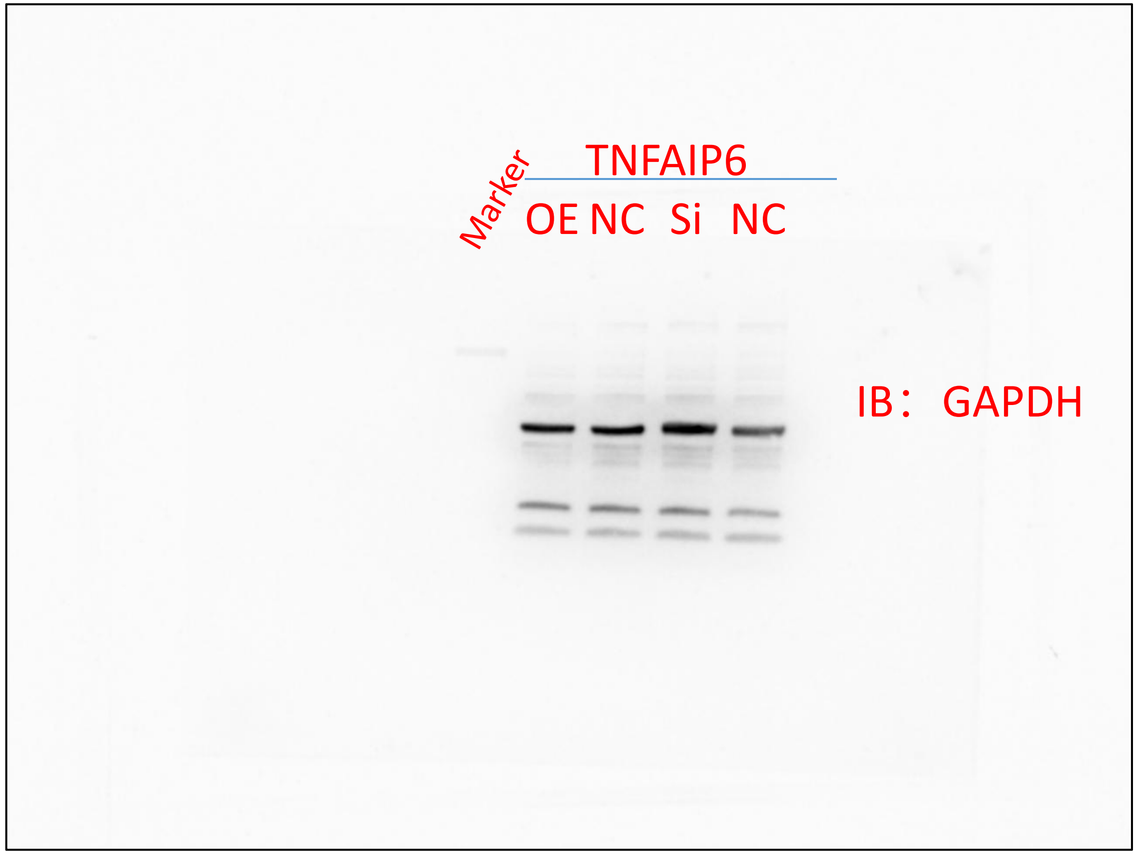

Without marker

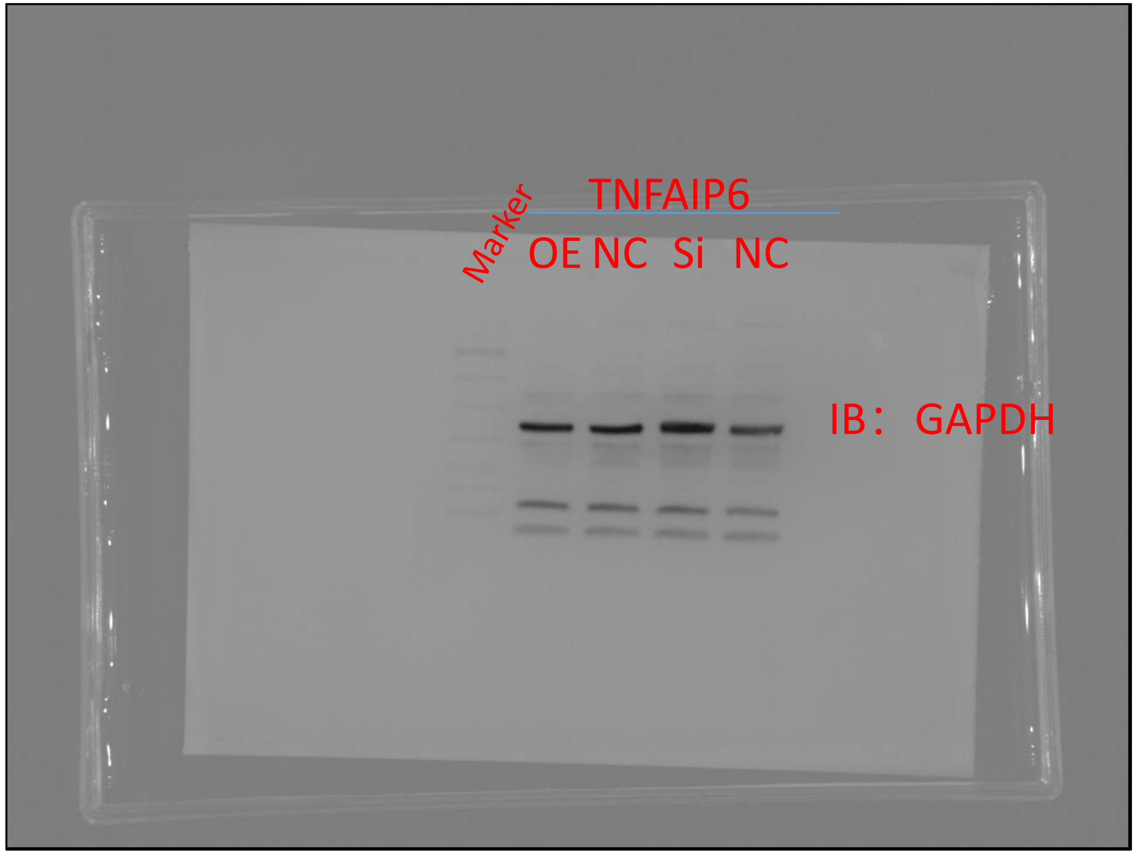

With marker

Fig 3C A549 IB SPP1

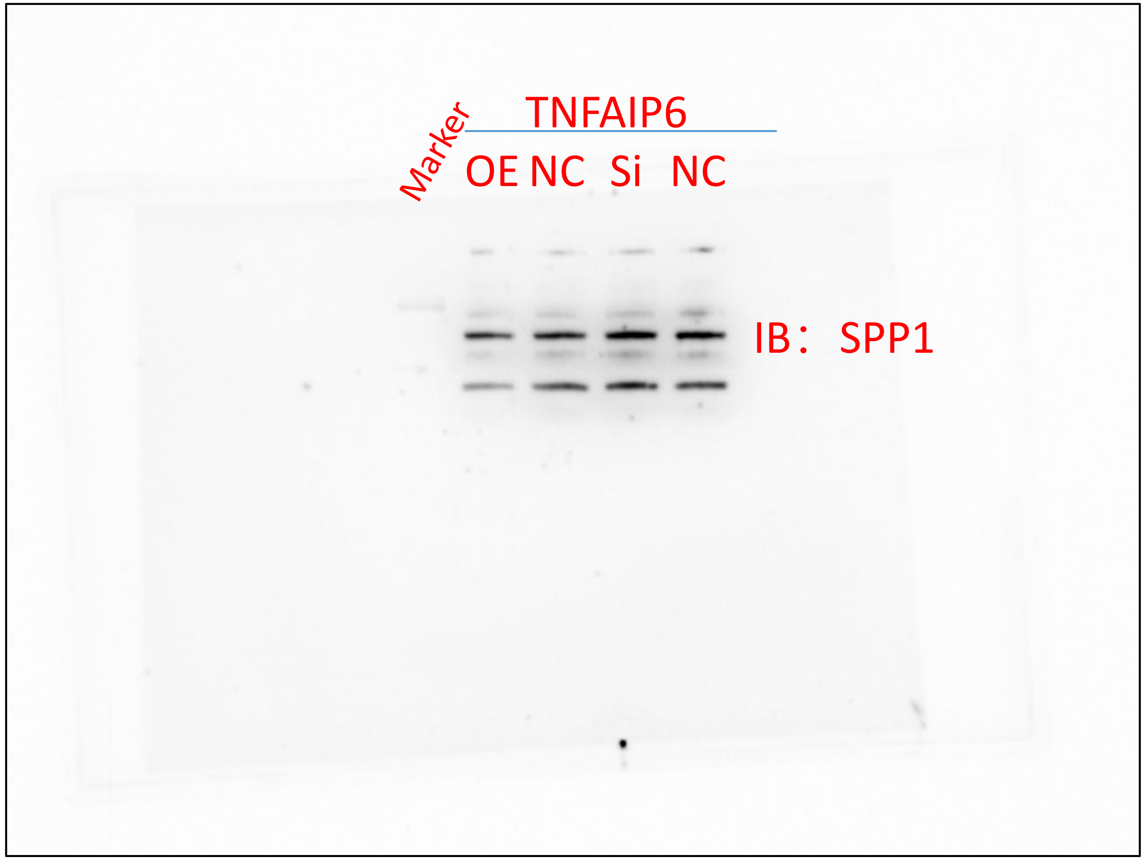

Without marker

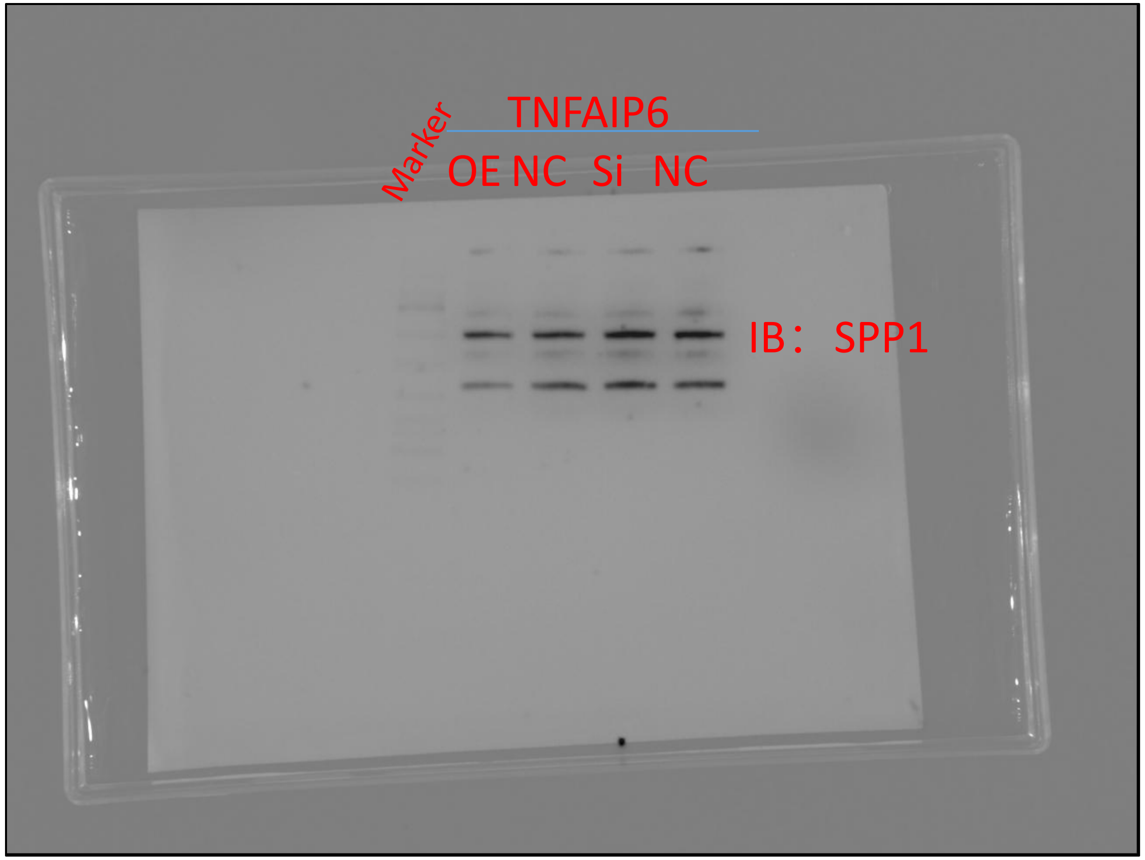

With marker

Fig 3C A549 IB GAPDH

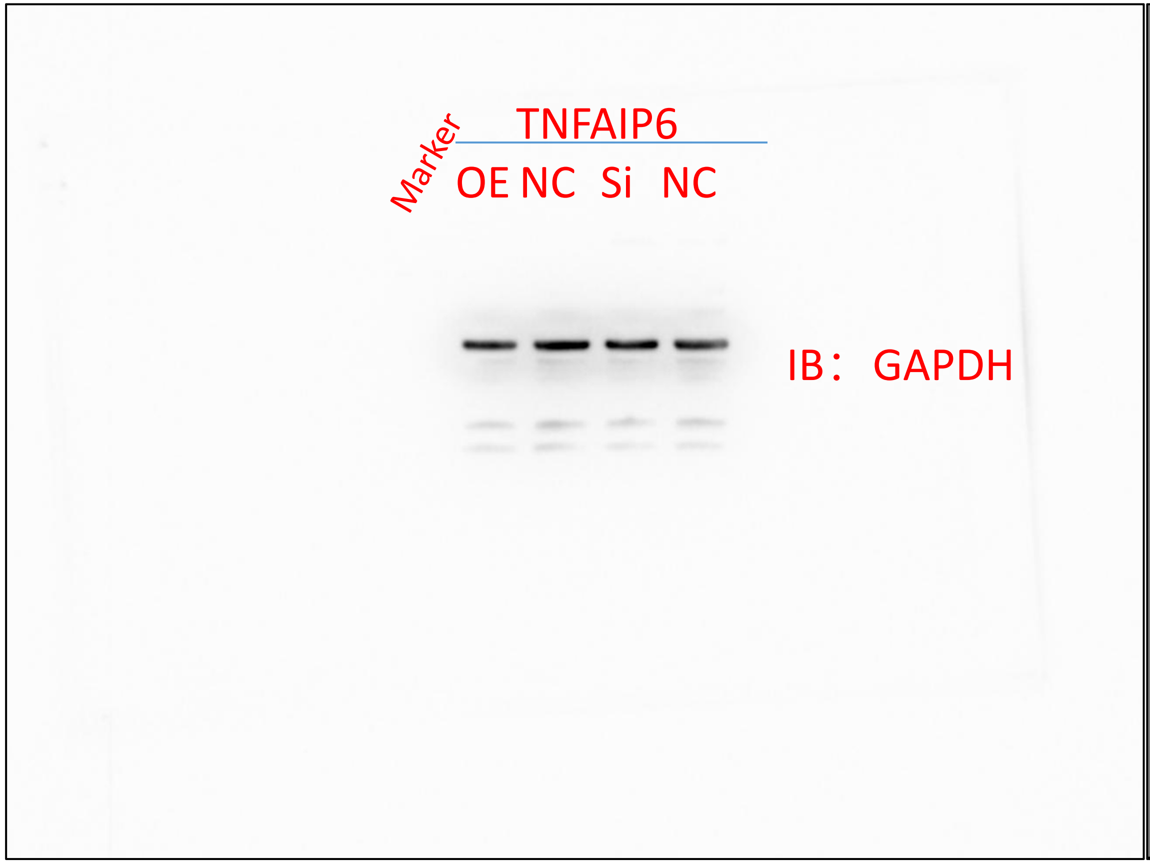

Without marker

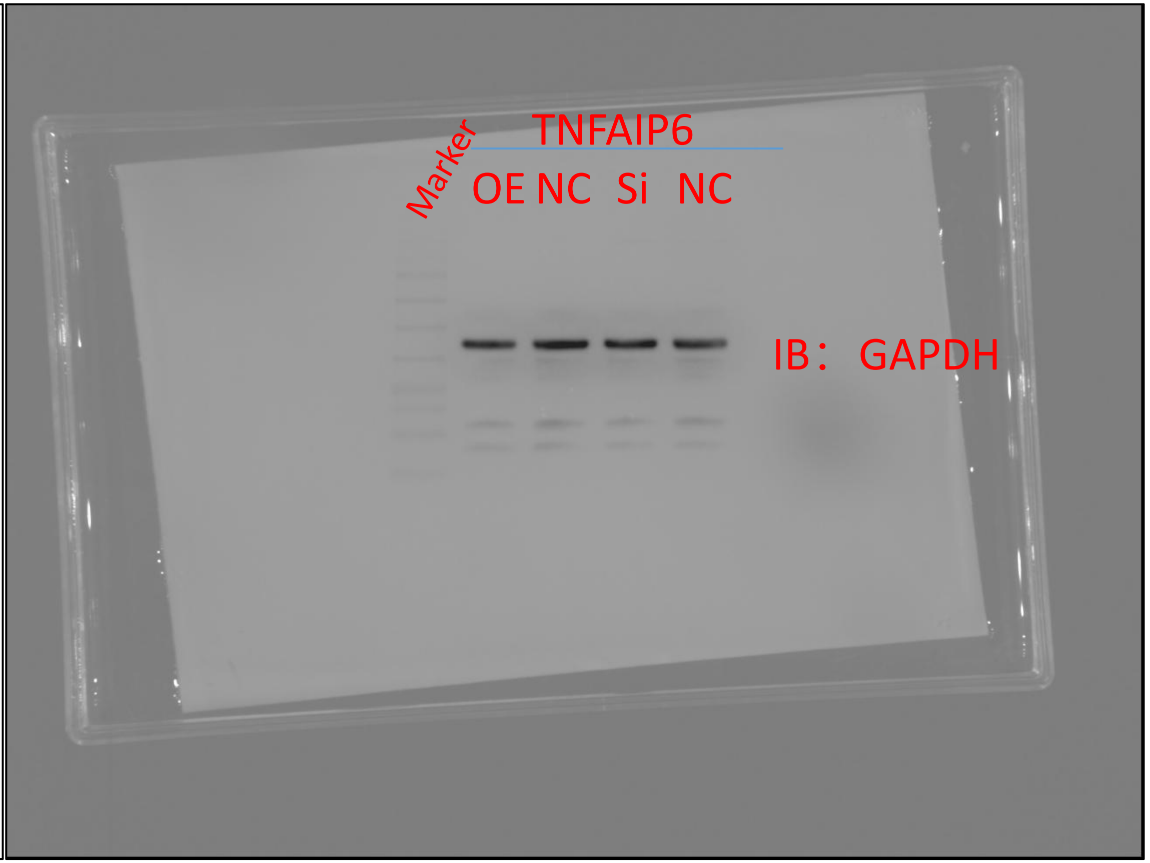

With marker

Fig 3C PC9 IB SPP1

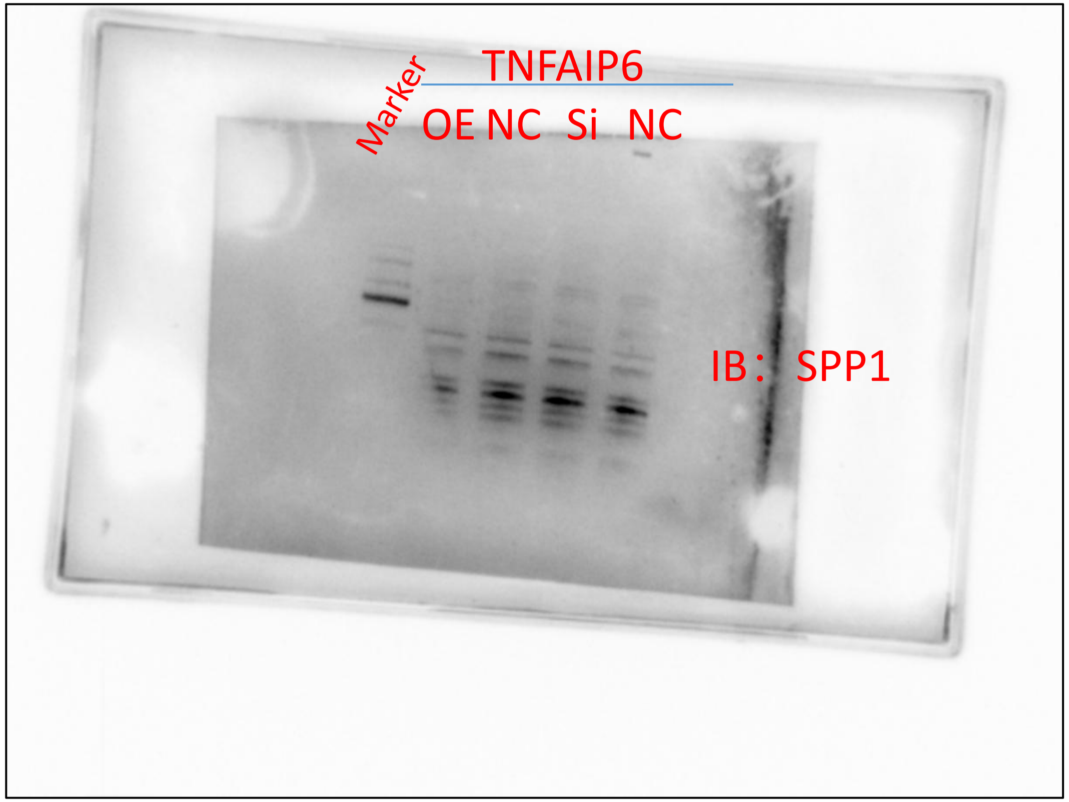

Without marker

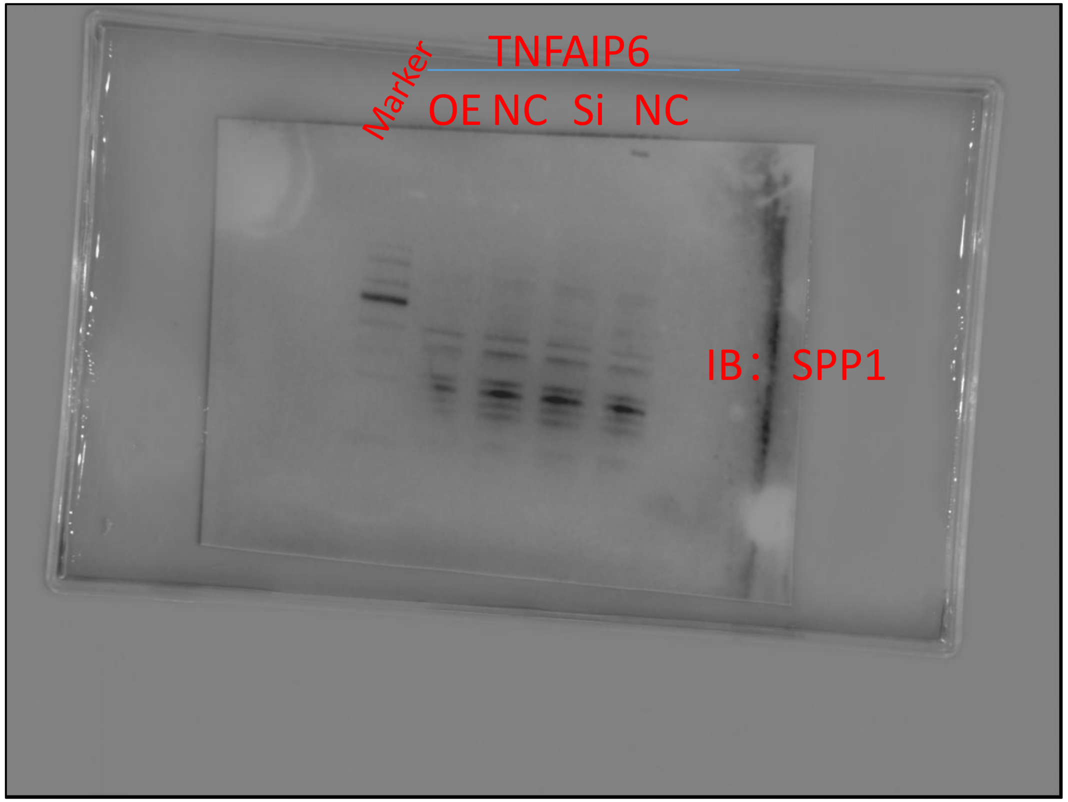

With marker

Fig 3C PC9 IB GAPDH

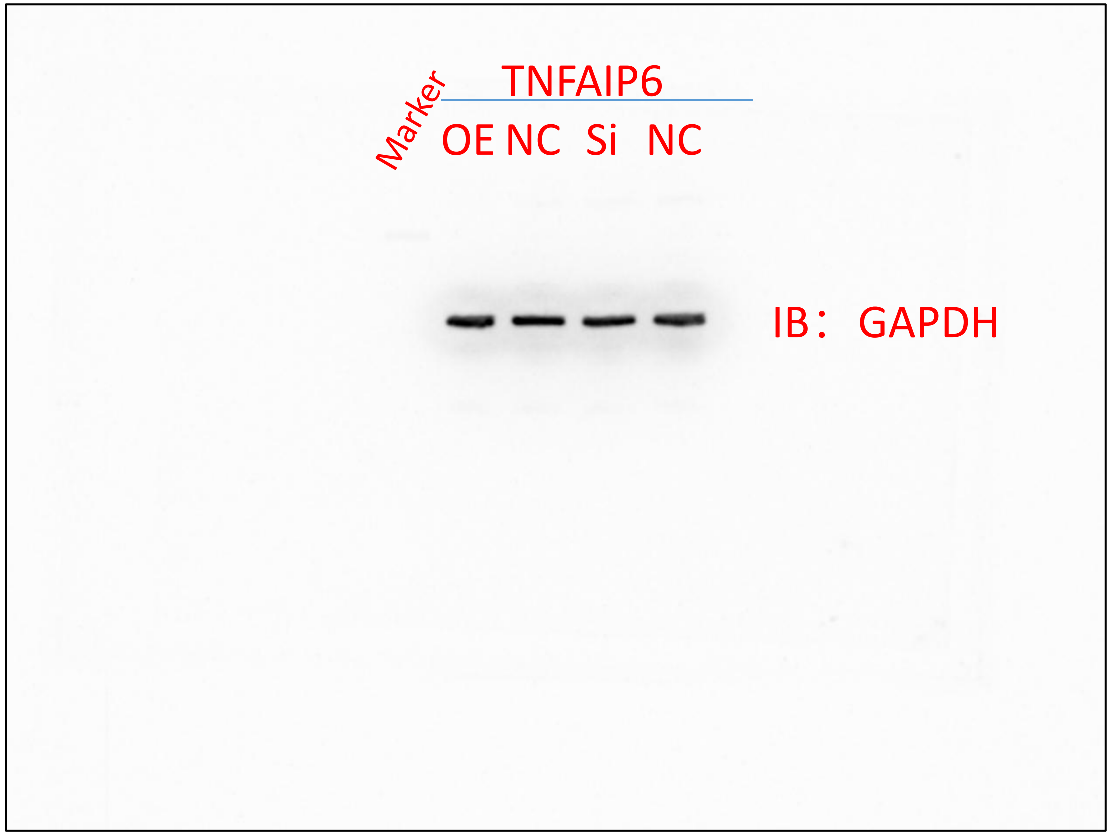

Without marker

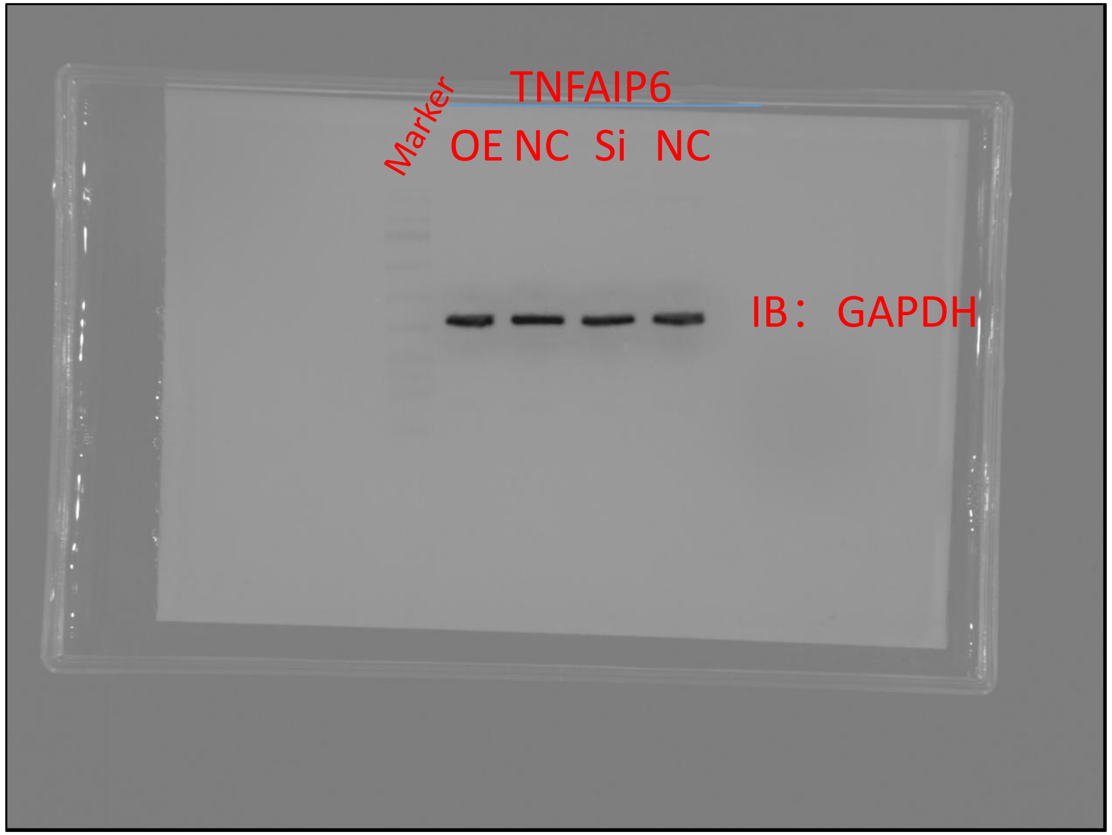

With marker

Fig 4C *i* IB CD44

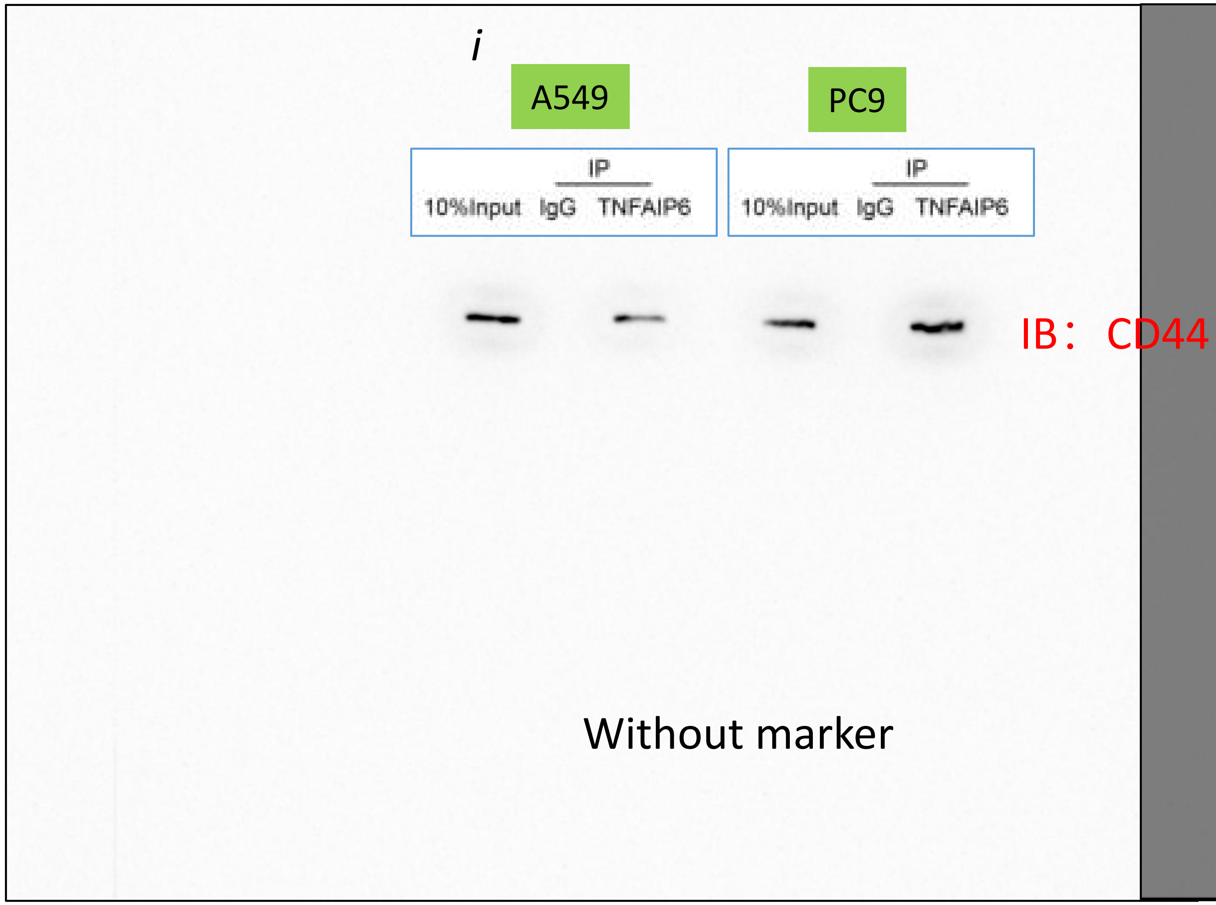

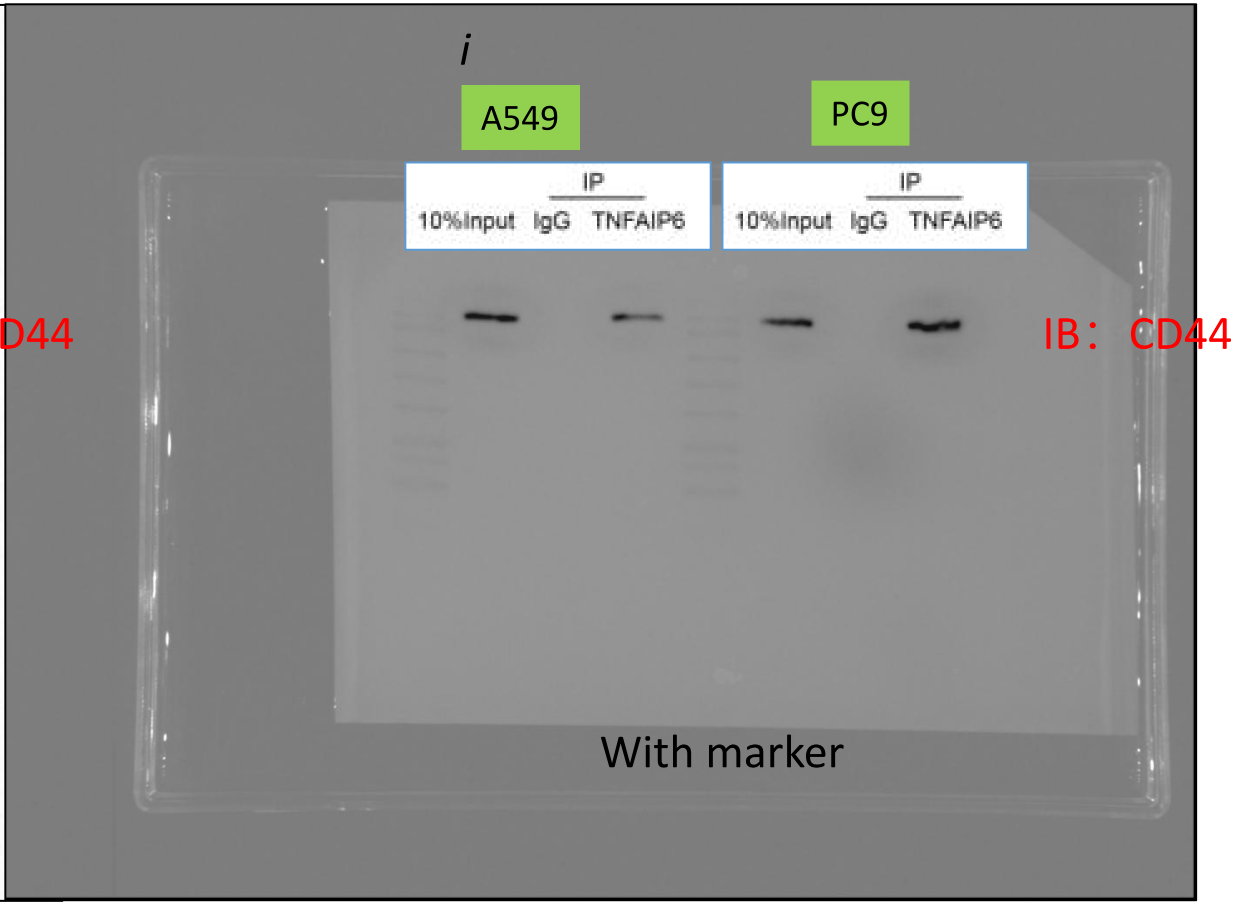

Fig 4C *i* IB TNFAIP6

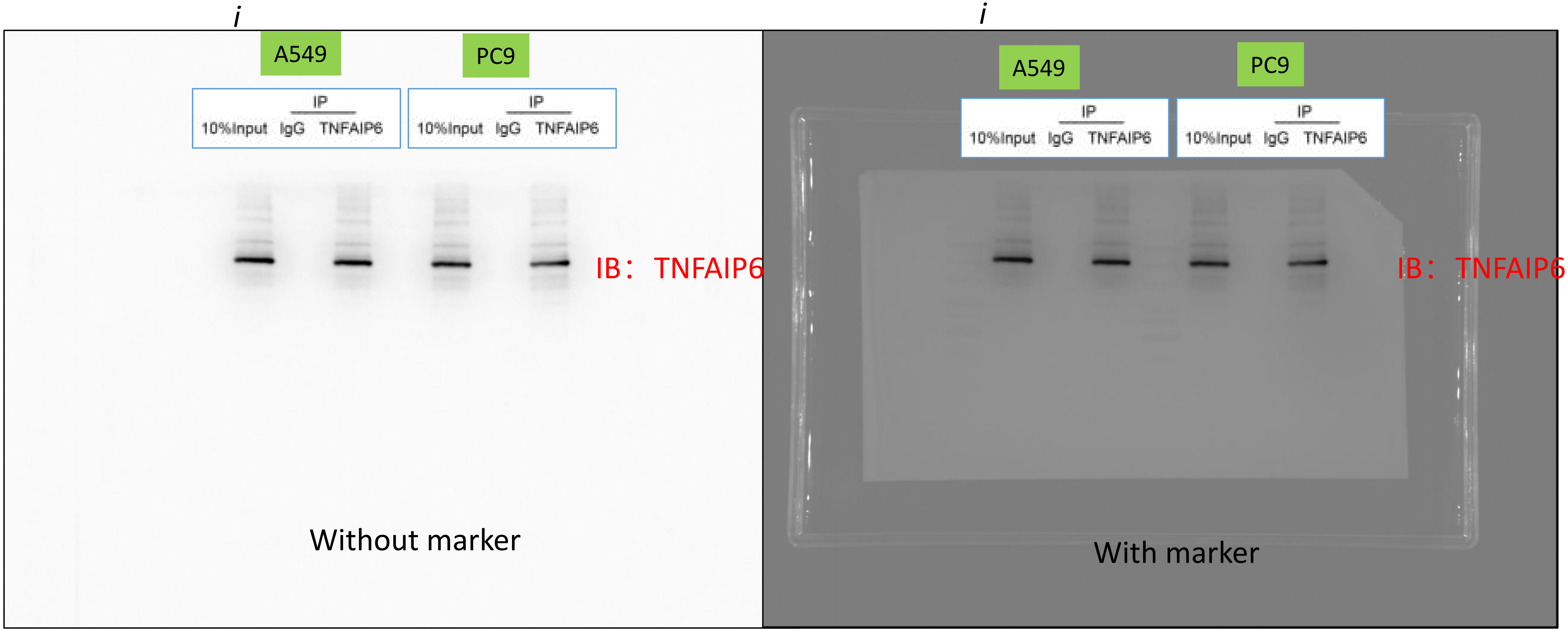

Fig 4C *i* IB GAPDH

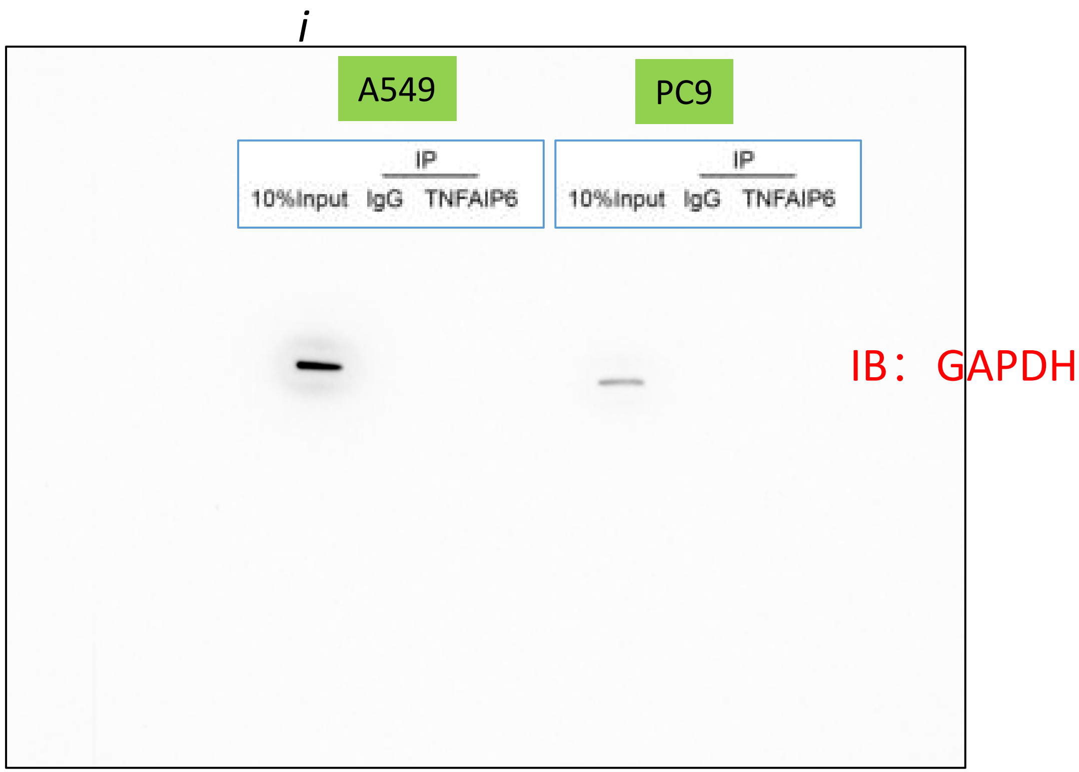

Without marker

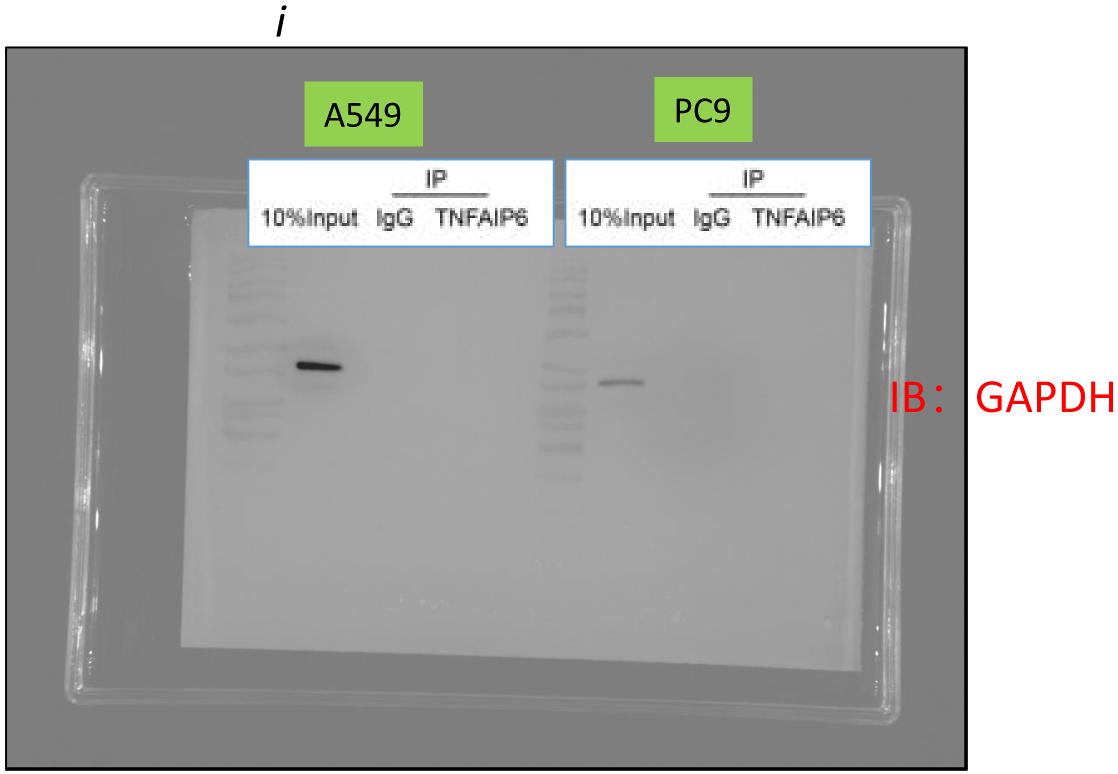

With marker

Fig 4C *ii* IB CD44

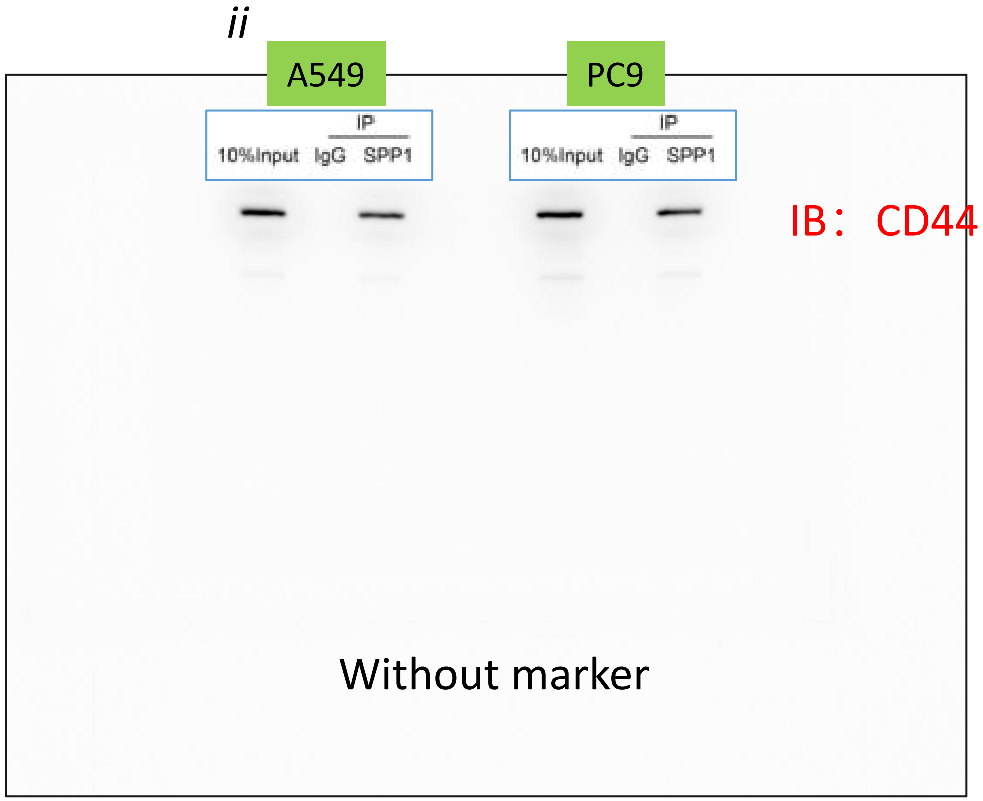

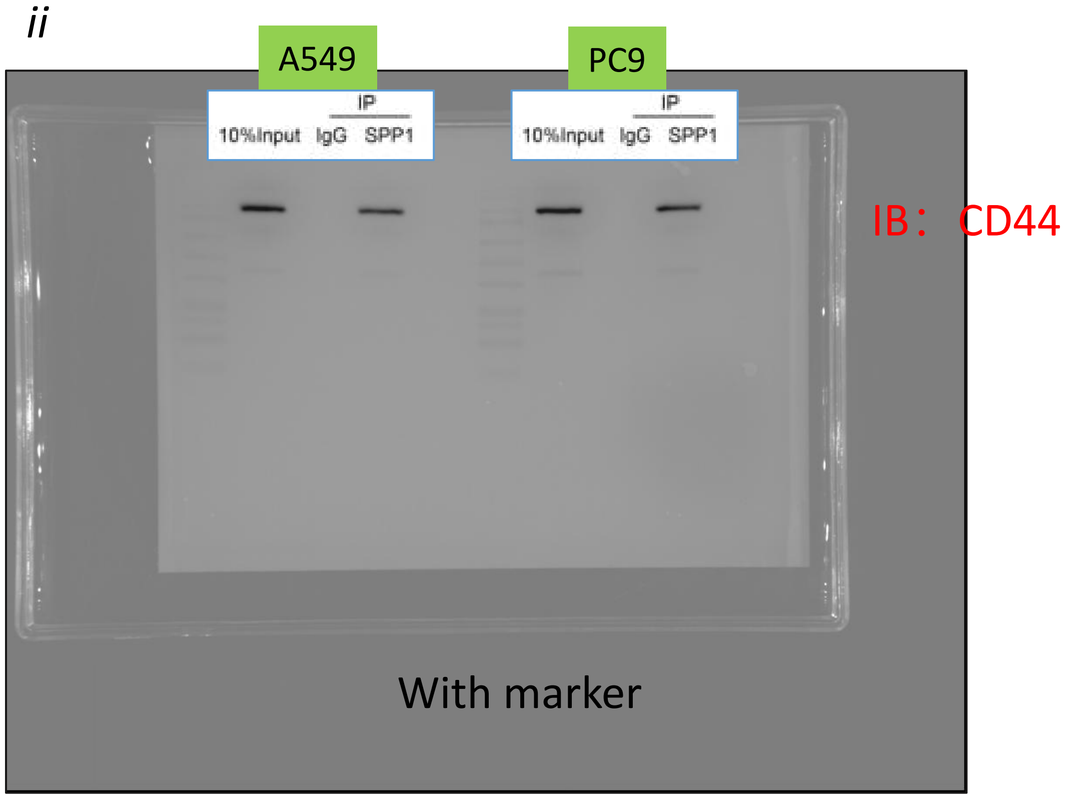

Fig 4C *ii* IB SPP1

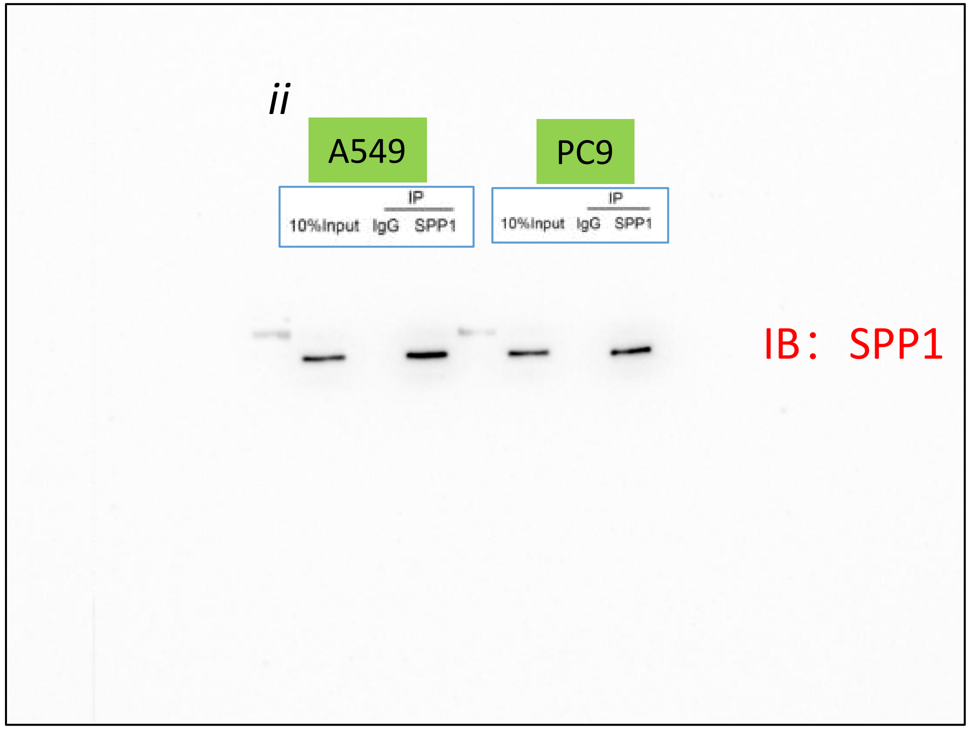

Without marker

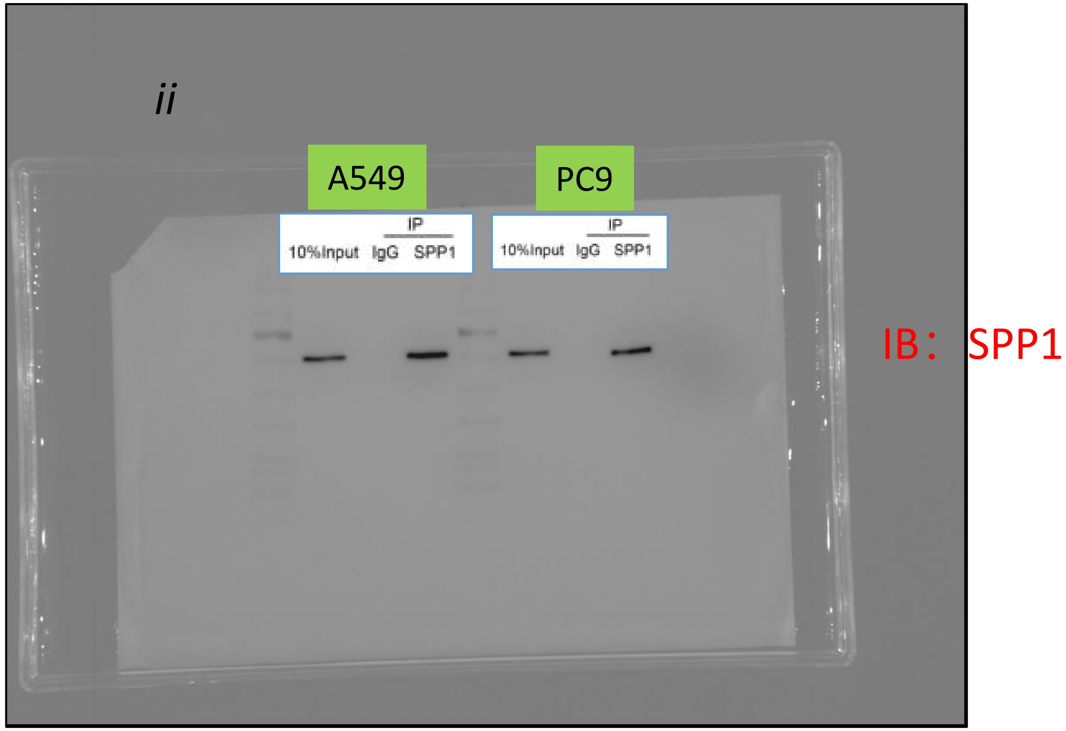

With marker

Fig 4C *ii* IB GAPDH

ii

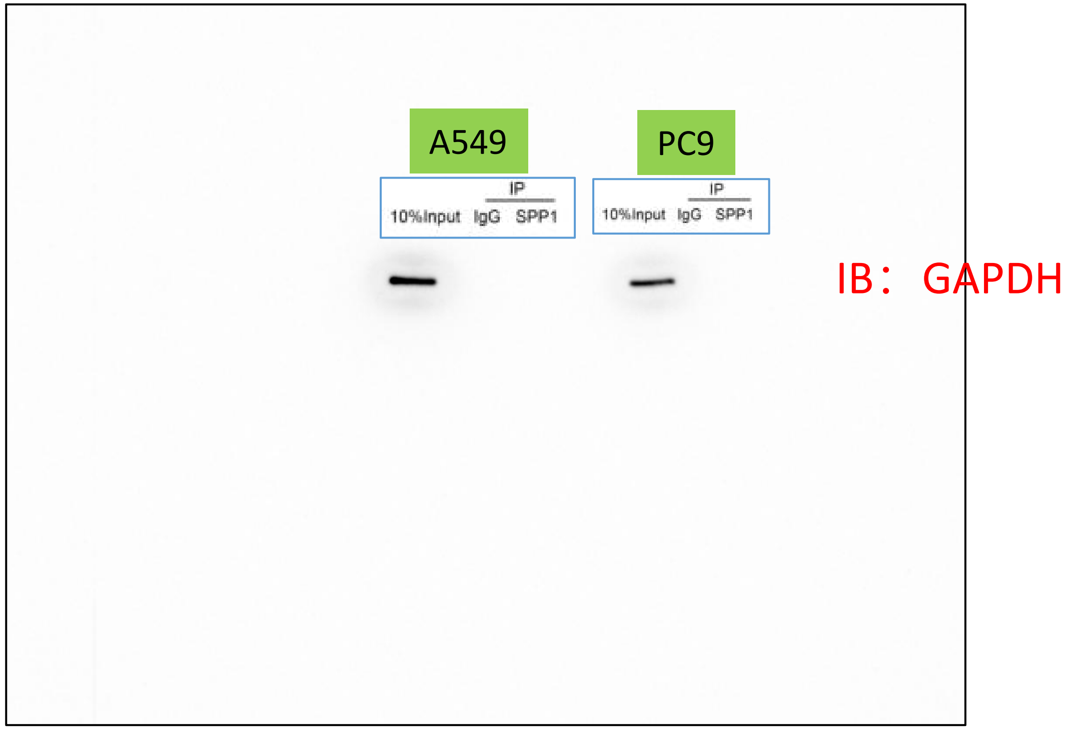

Without marker

ii

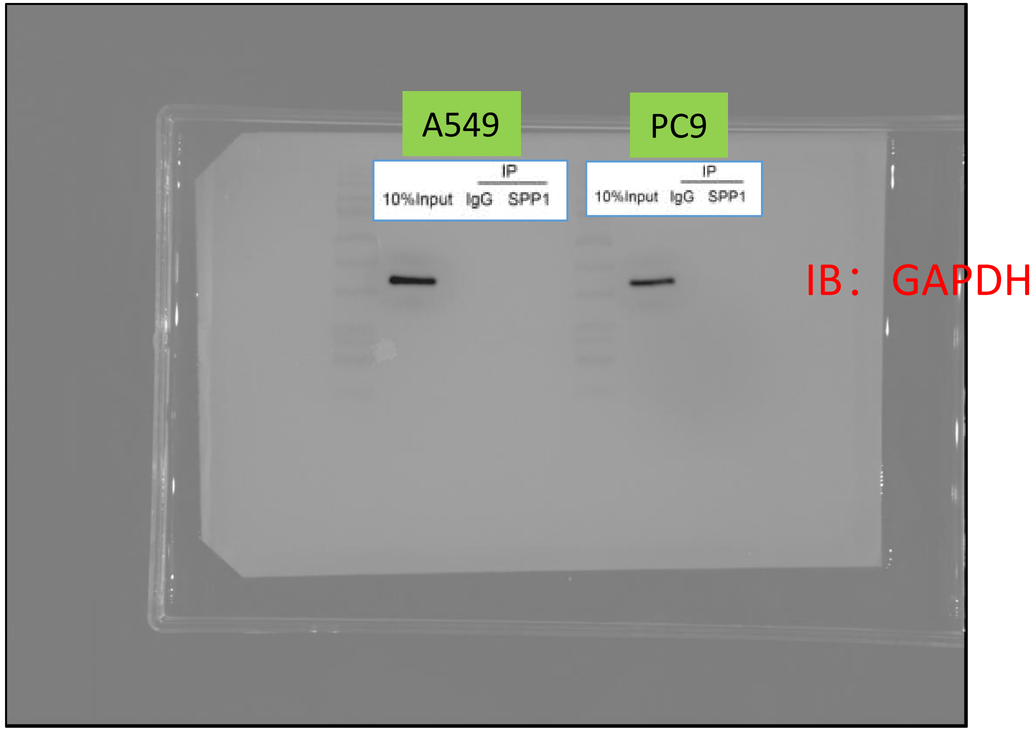

With marker

Fig 4D *i* IB SPP1 A549

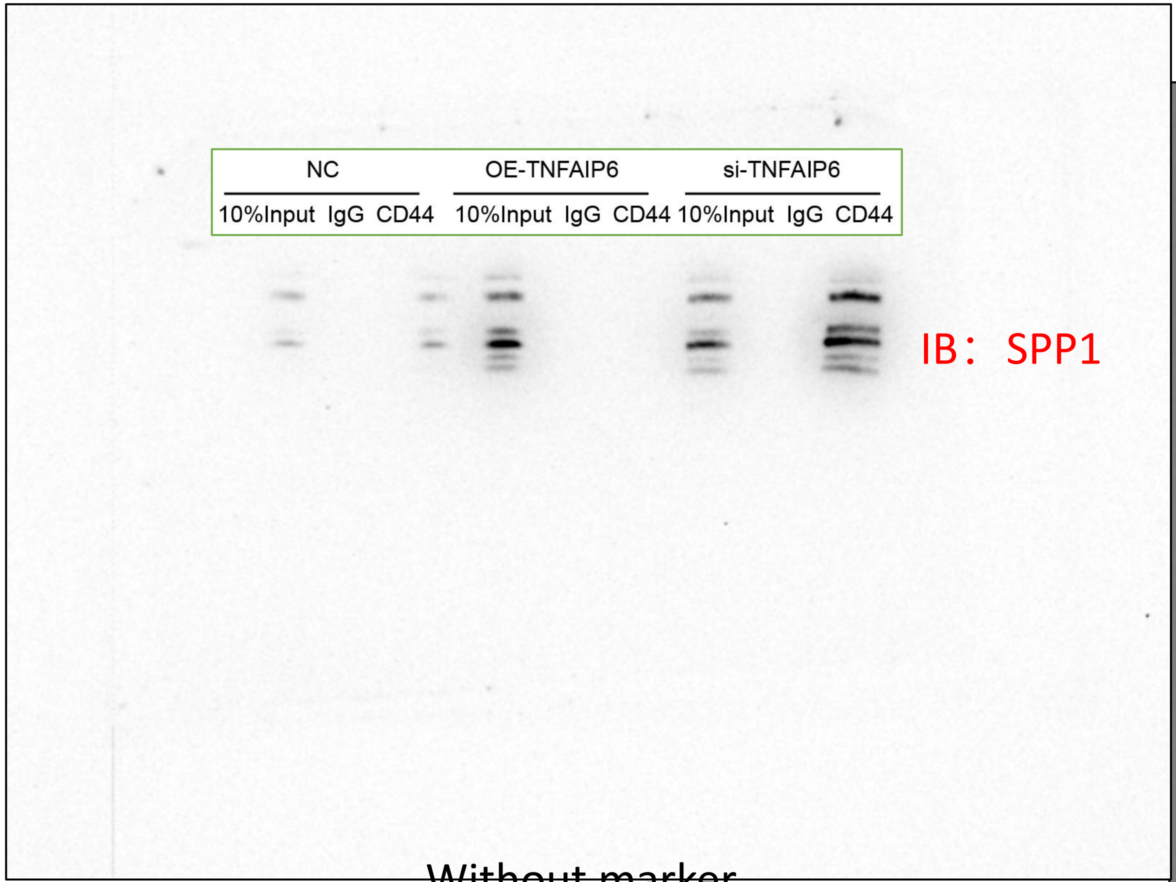

Without marker

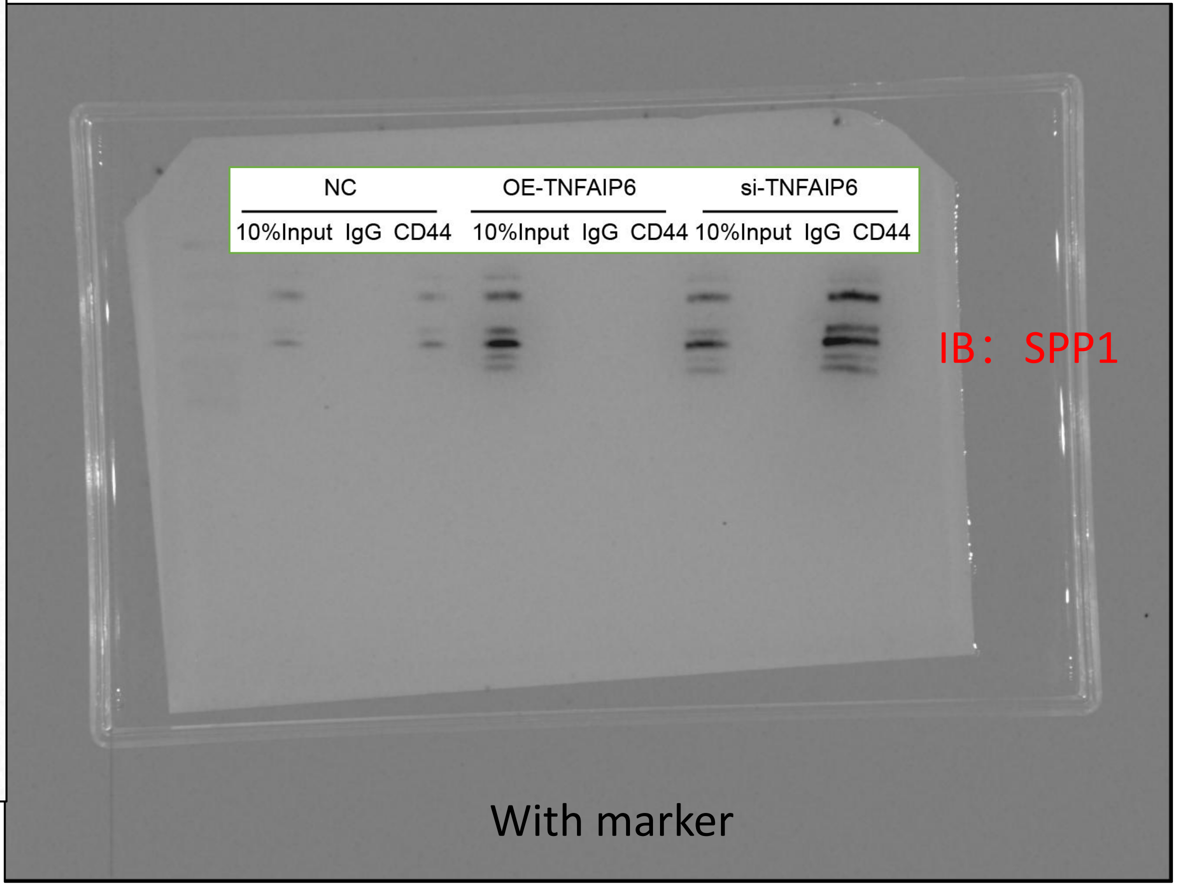

With marker

Fig 4D *i* IB CD44 A549

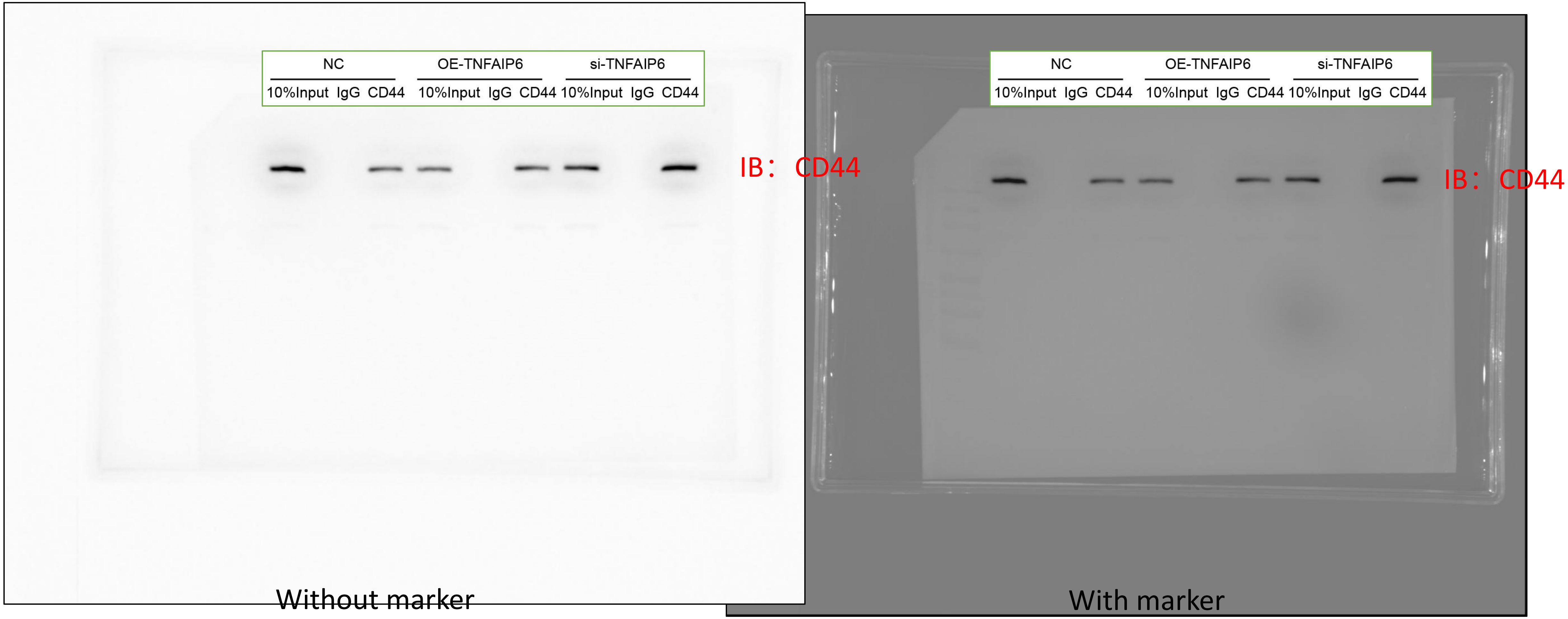

Fig 4D *i* IB GAPDH A549

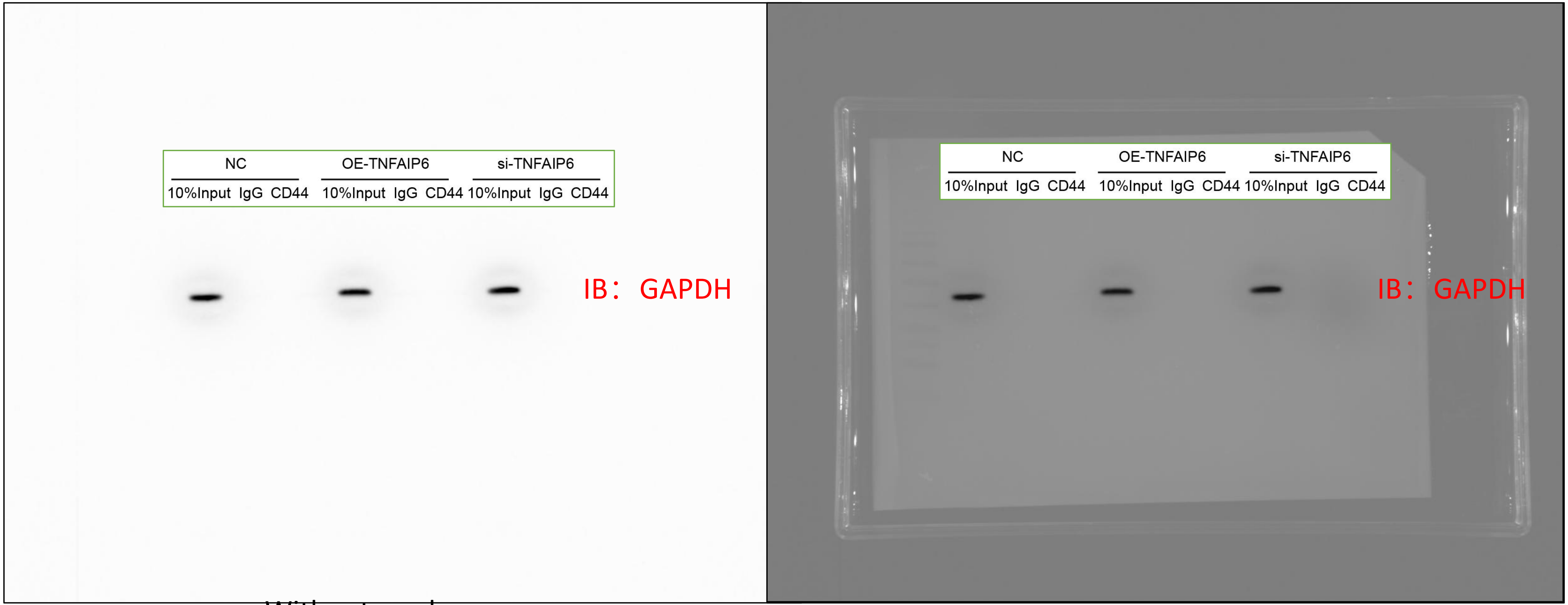

Without marker

With marker

Fig 4D *ii* IB SPP1 PC9

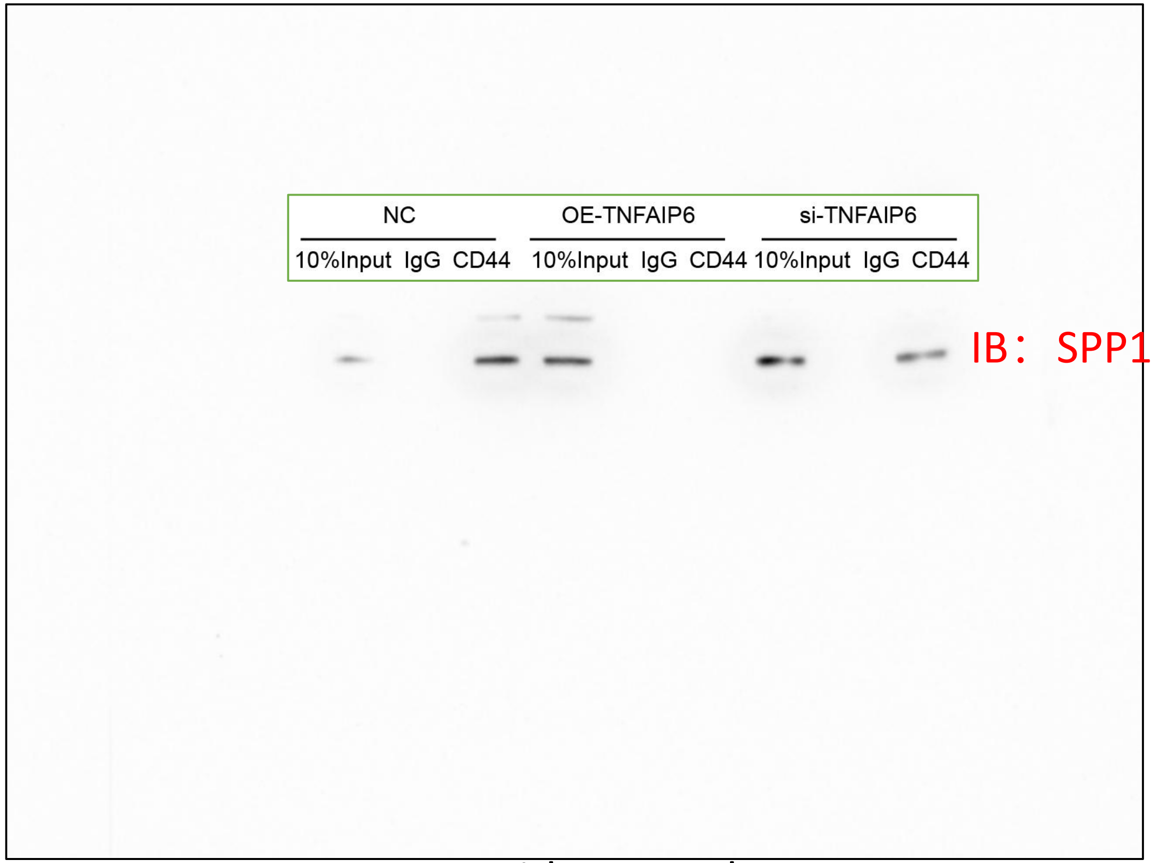

Without marker

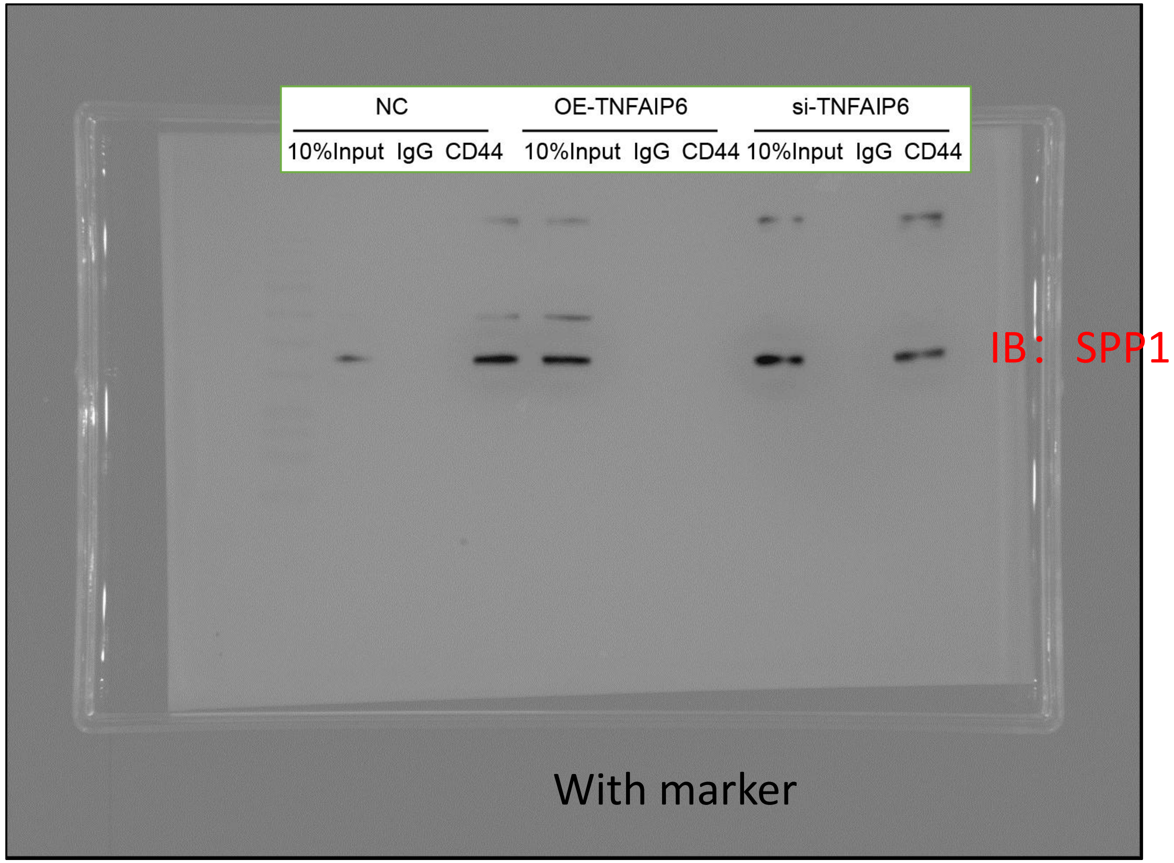

With marker

Fig 4D *ii* IB CD44 PC9

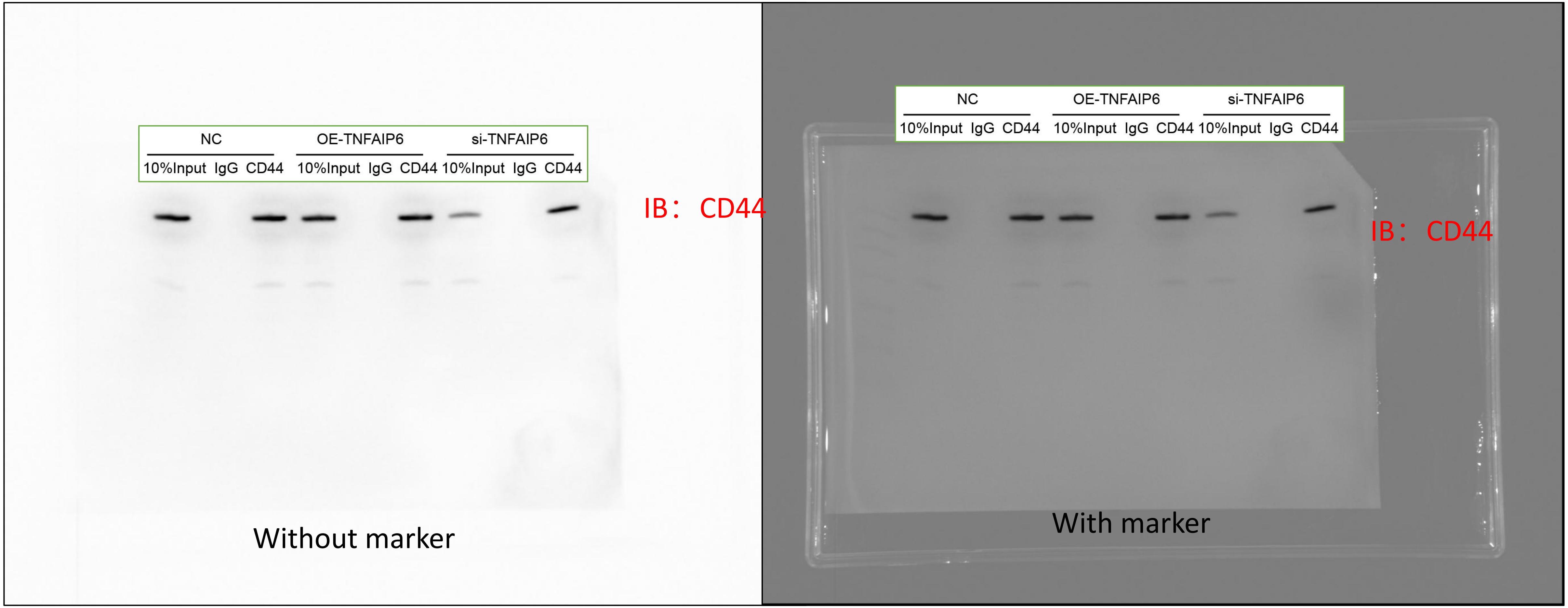

Fig 4D *ii* IB GAPDH PC9

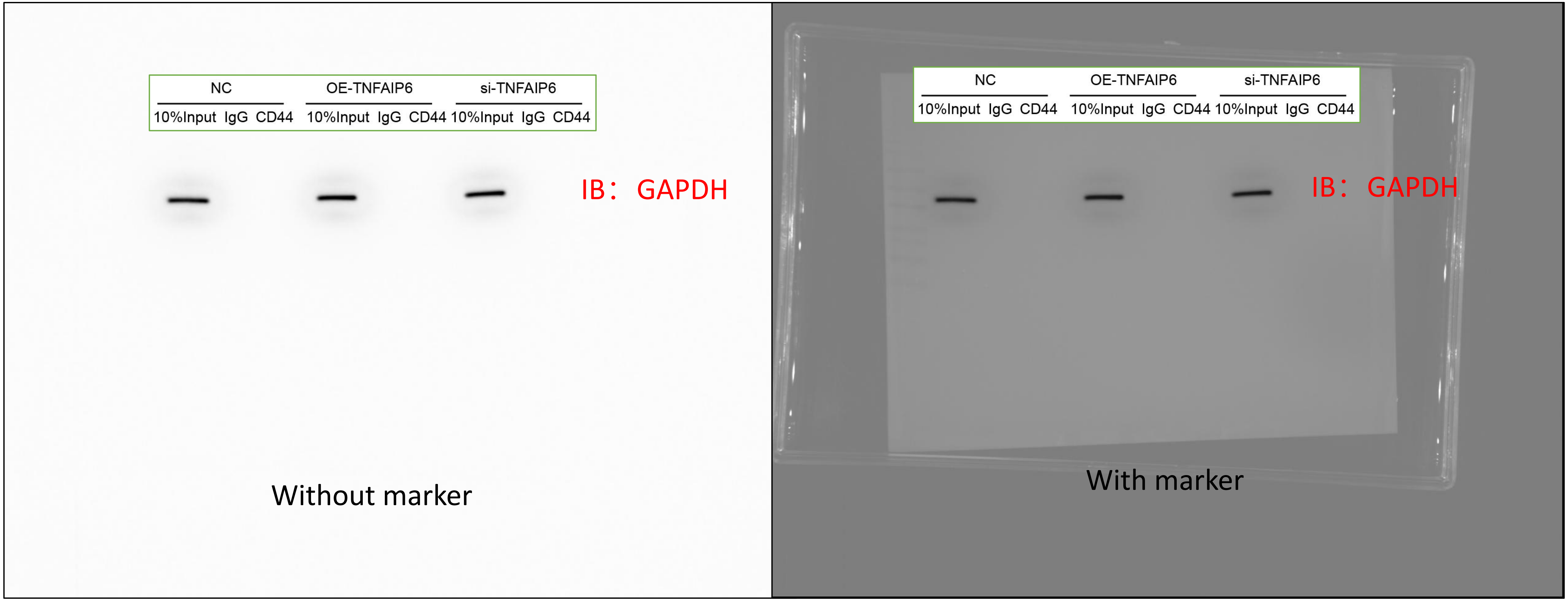

Fig 4E *i* IB TNFAIP6 A549

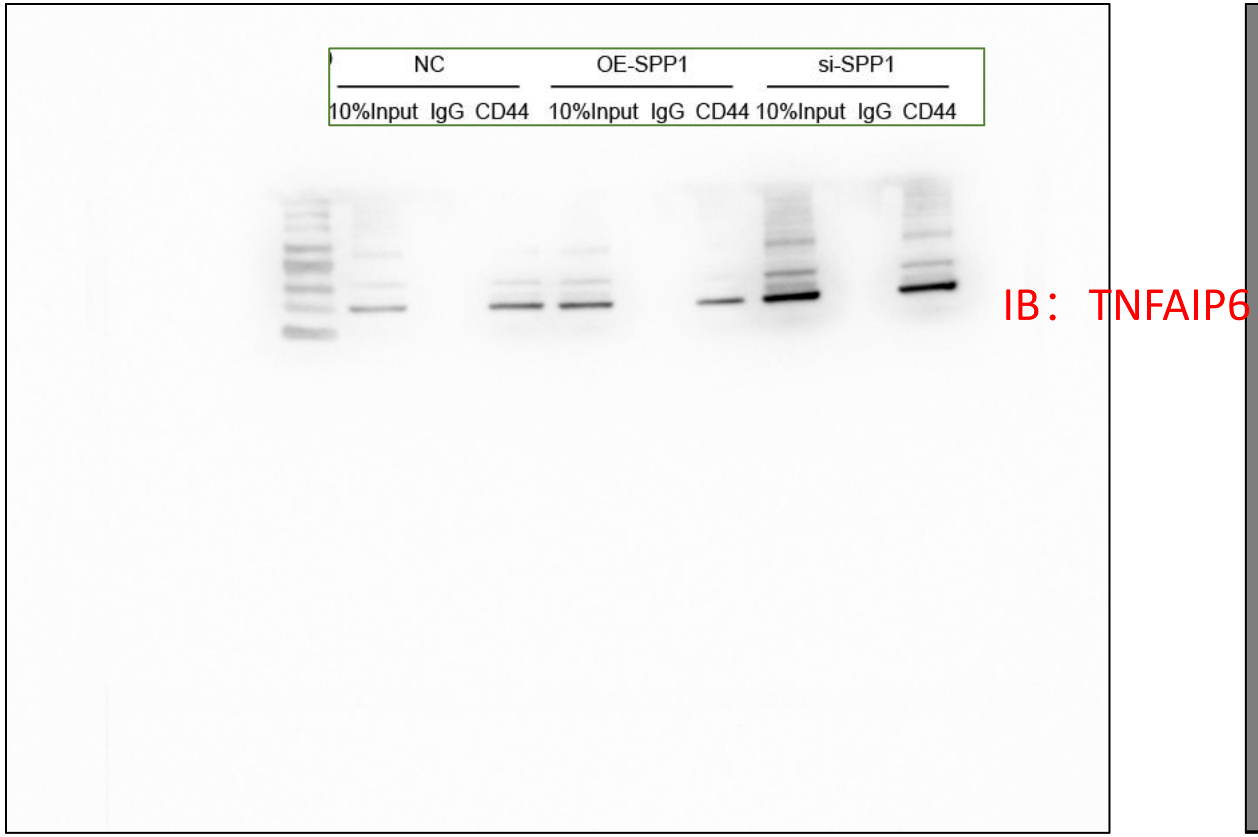

Without marker

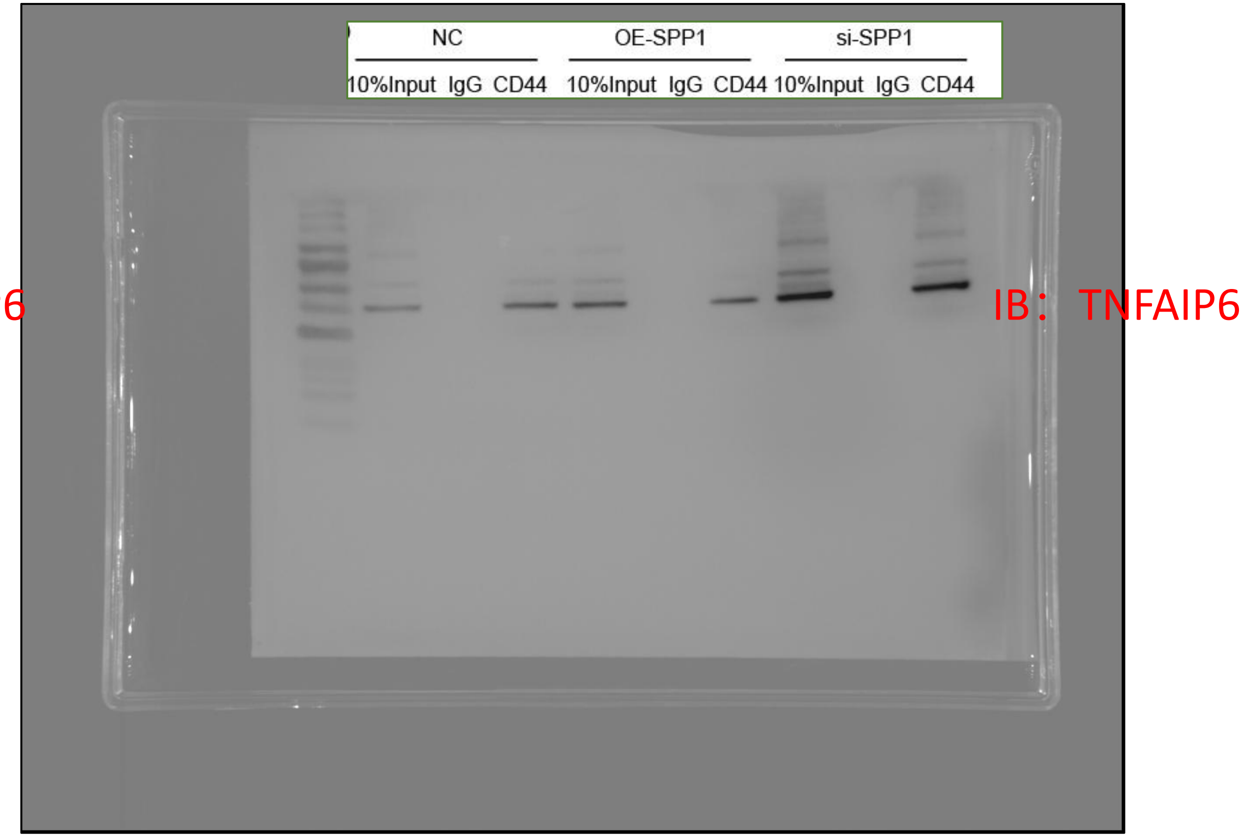

With marker

Fig 4E *i* IB CD44 A549

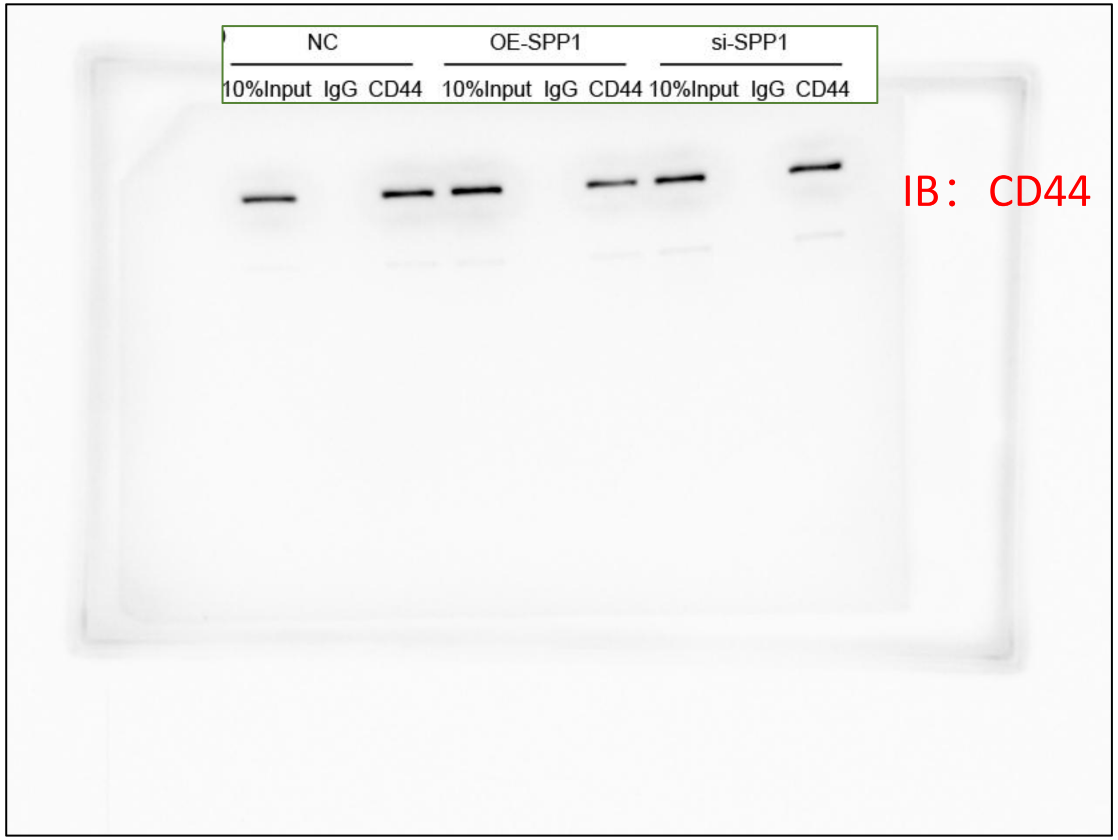

Without marker

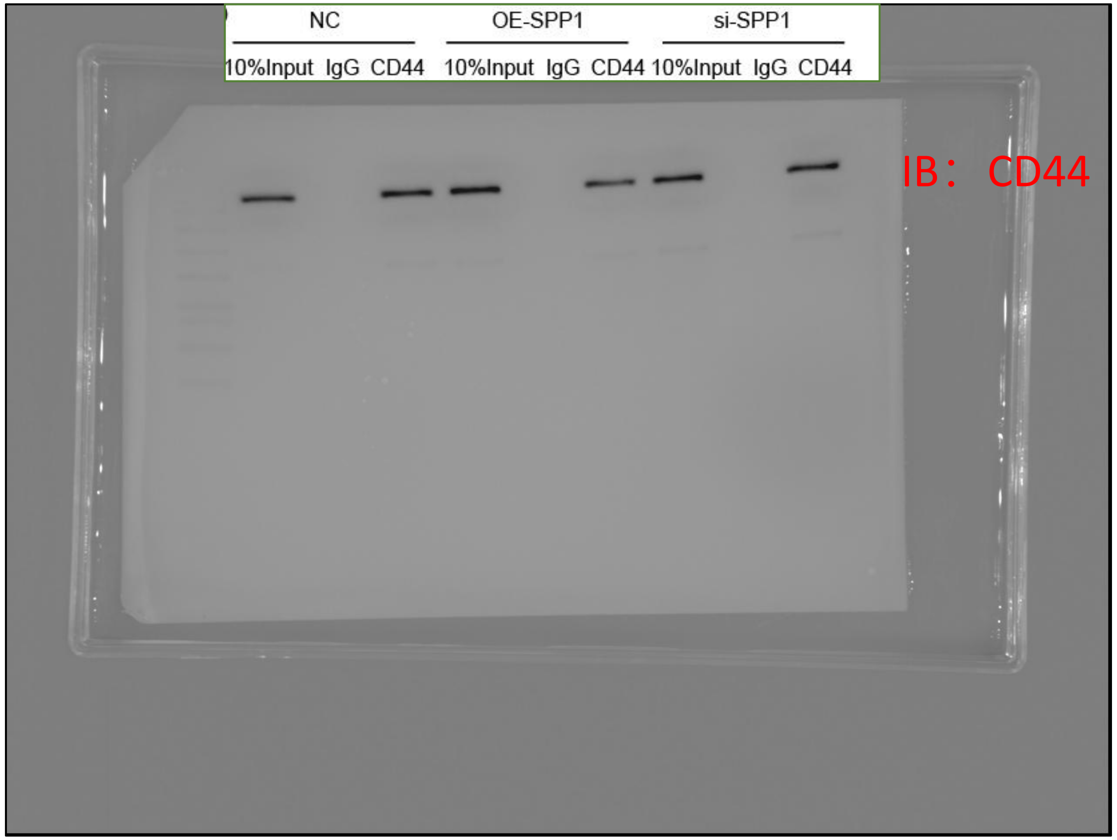

With marker

Fig 4E *i* IB GAPDH A549

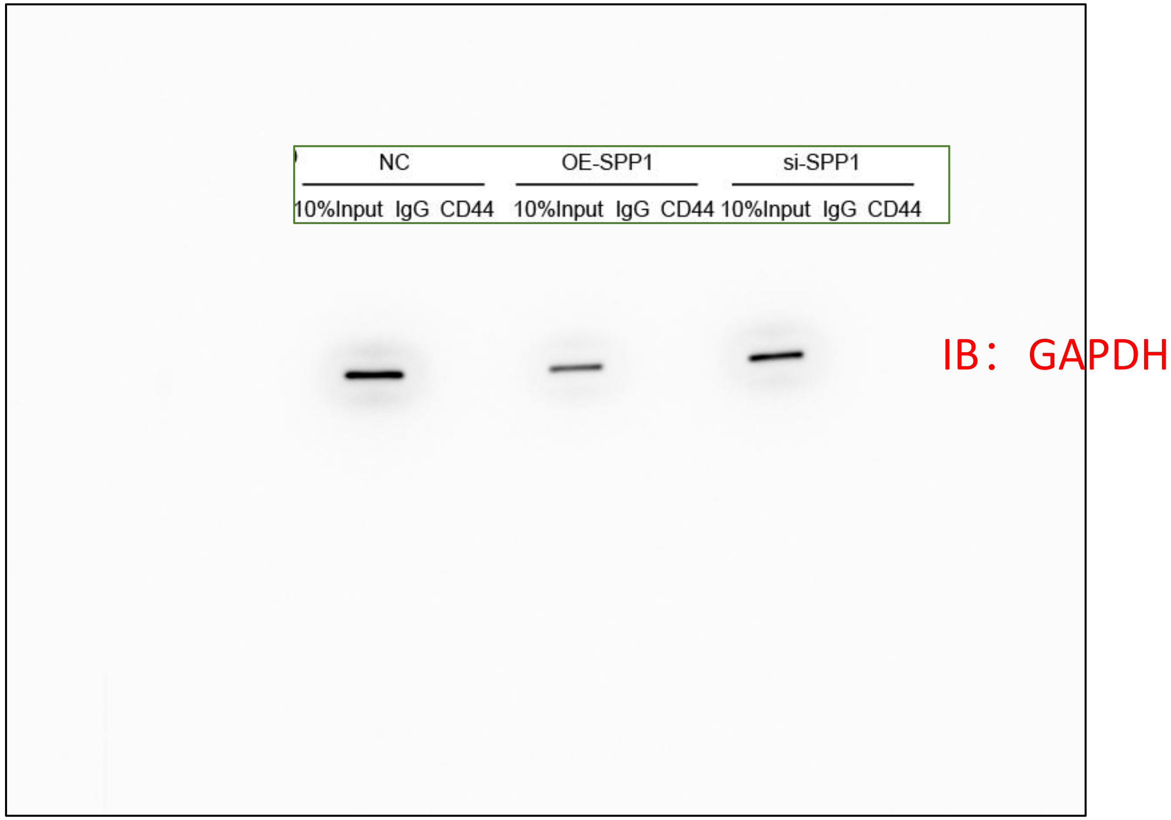

Without marker

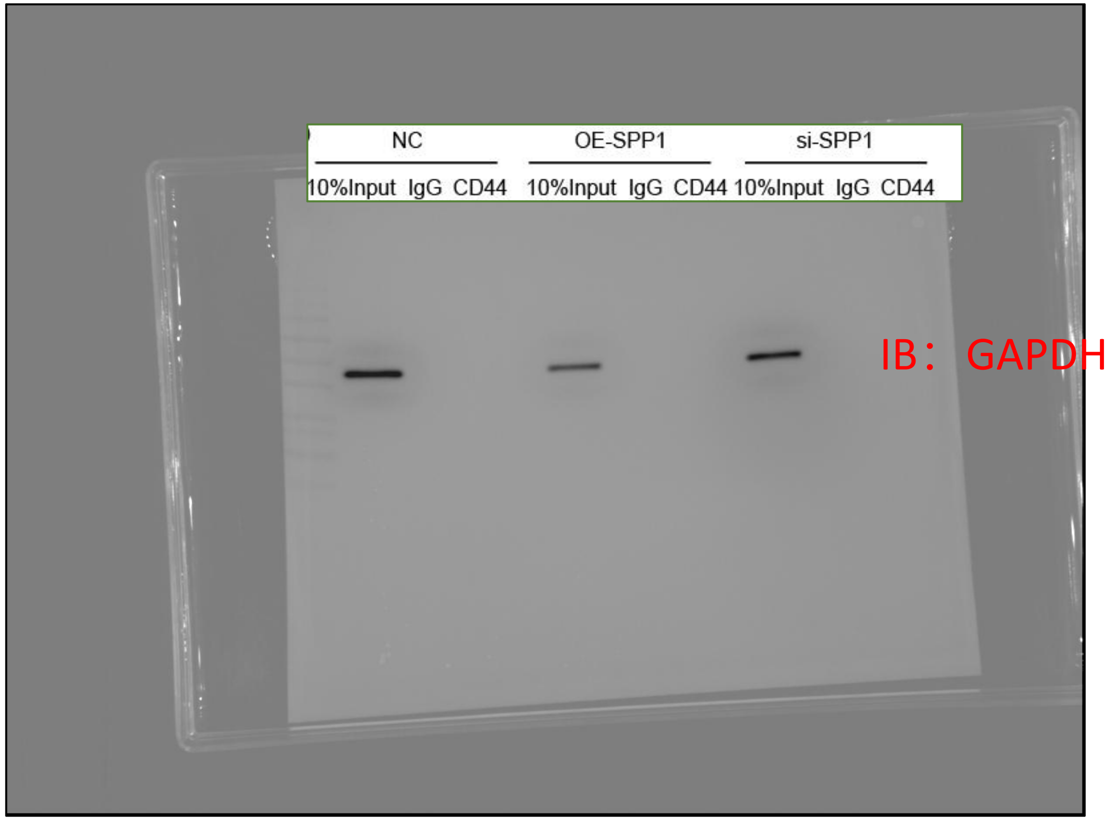

With marker

Fig 4E *ii* IB TNFAIP6 PC9

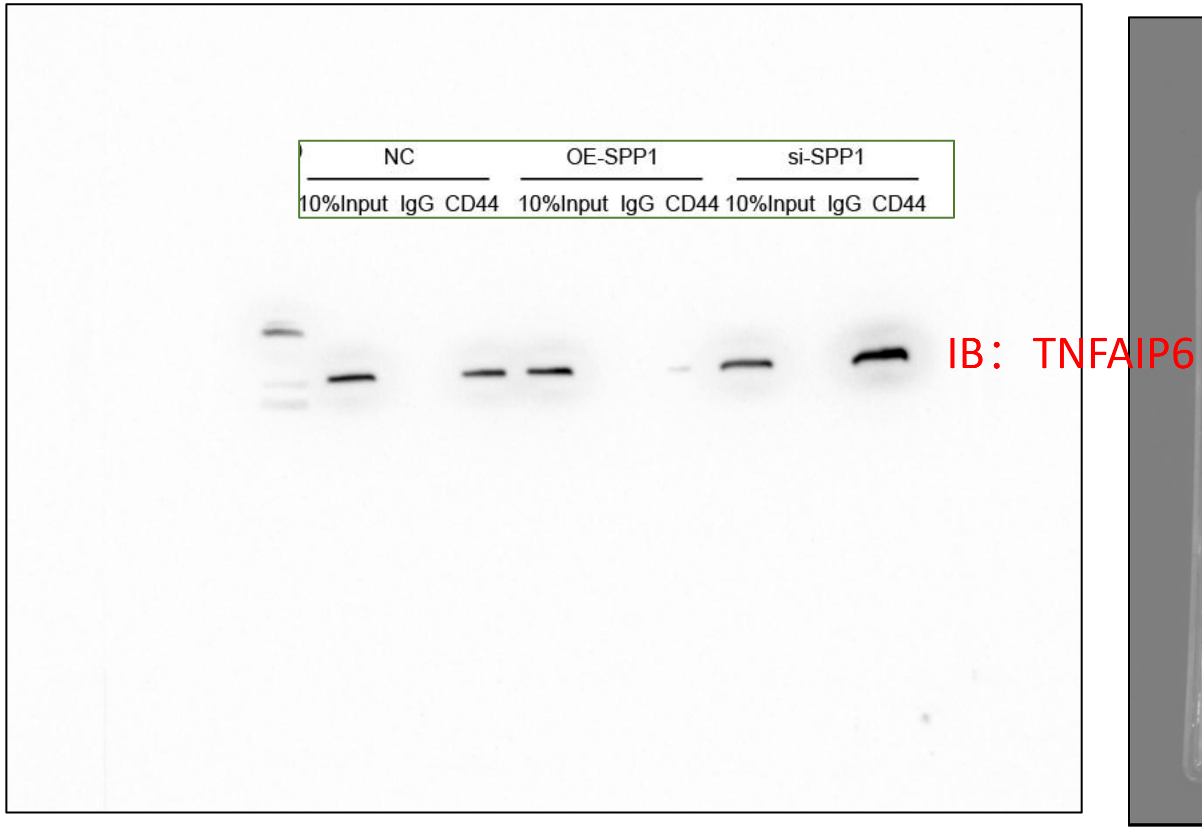

Without marker

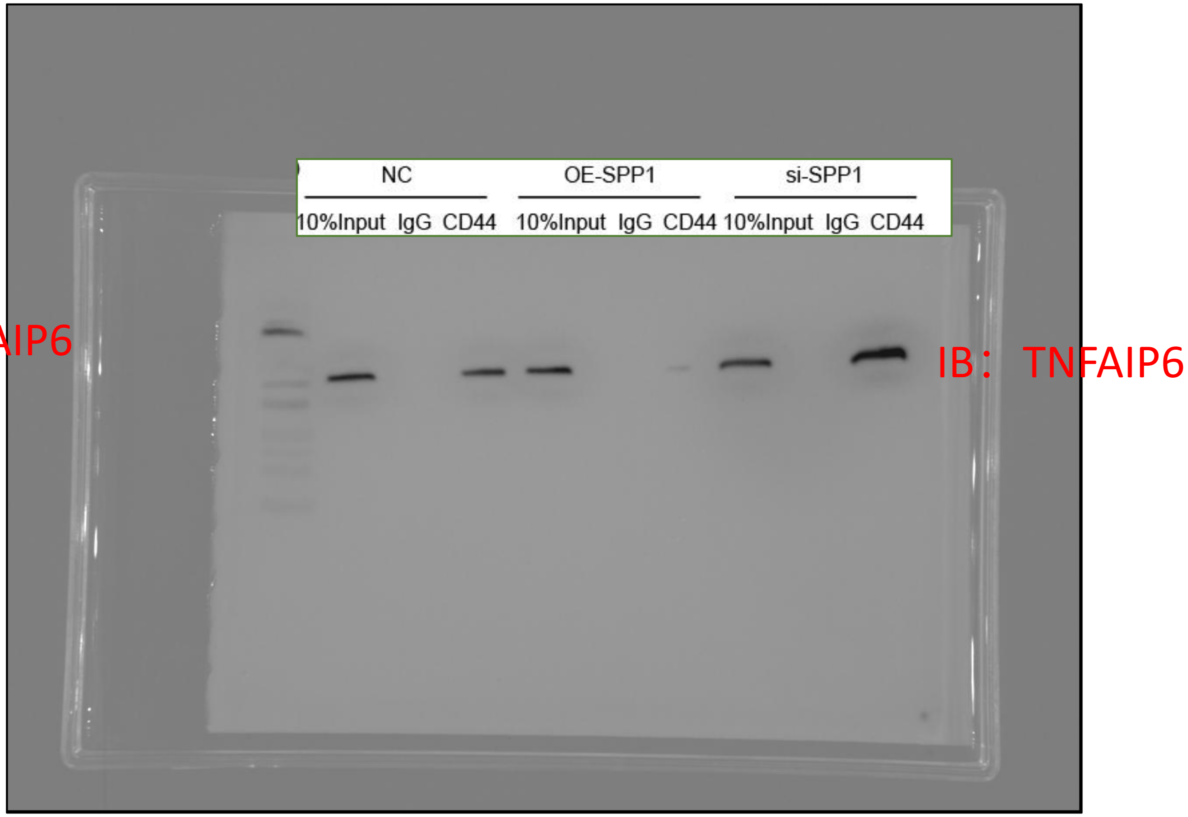

With marker

Fig 4E *ii* IB CD44 PC9

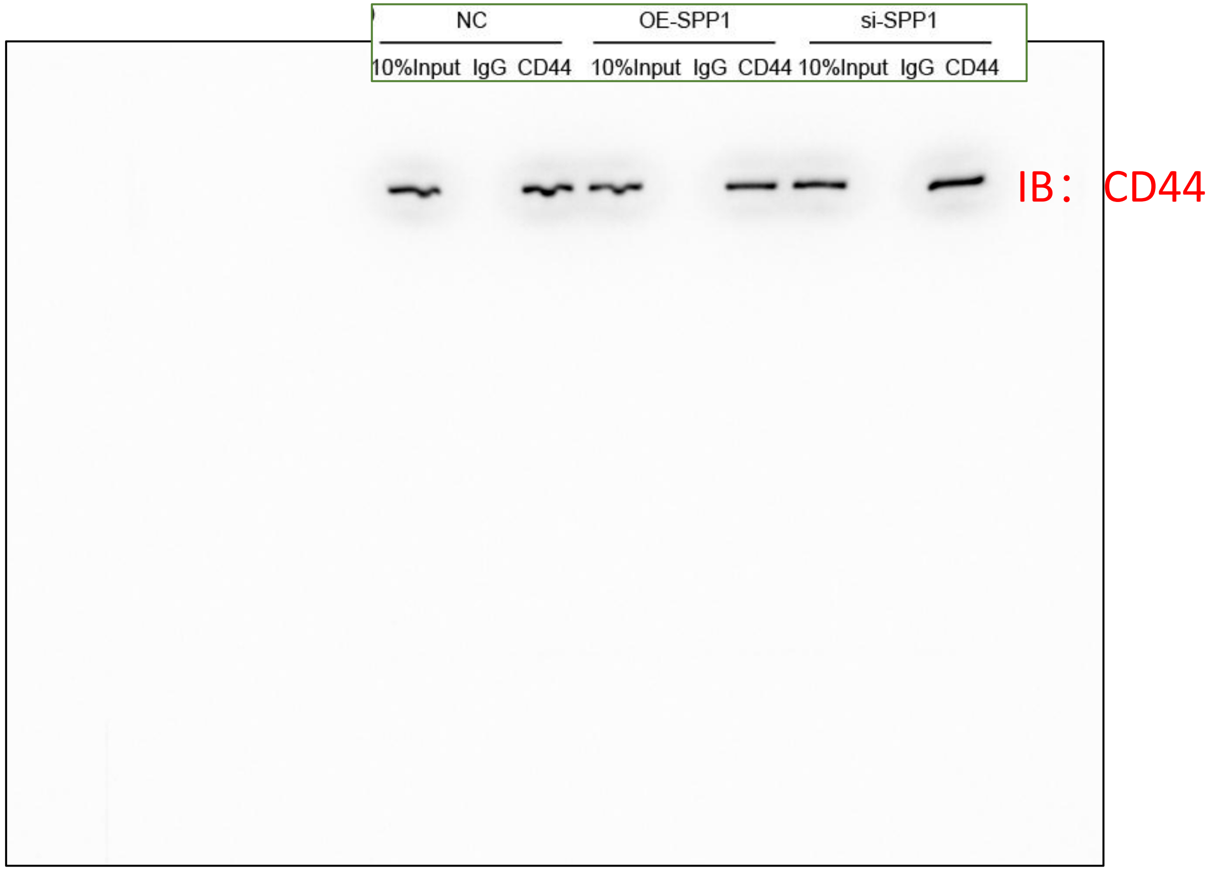

Without marker

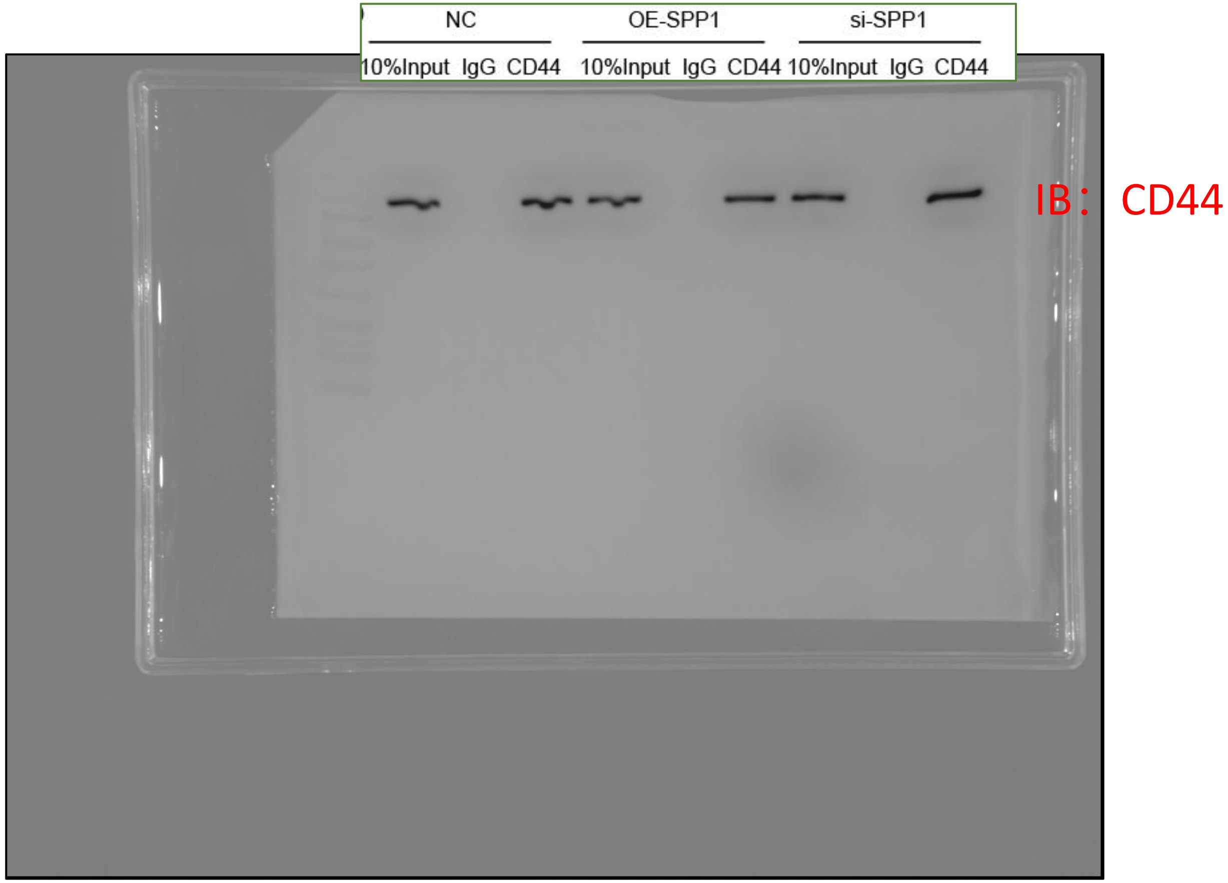

With marker

Fig 4E *ii* IB GAPDH PC9

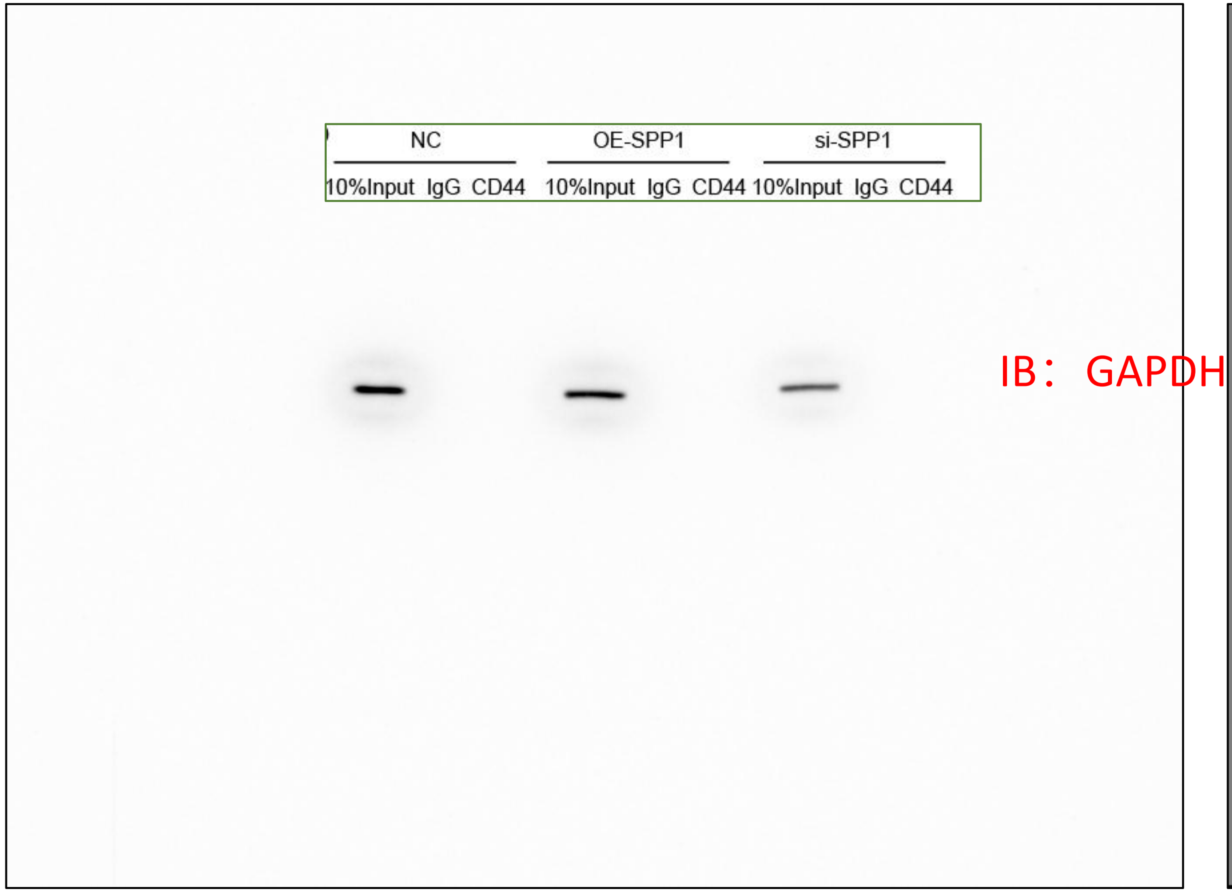

Without marker

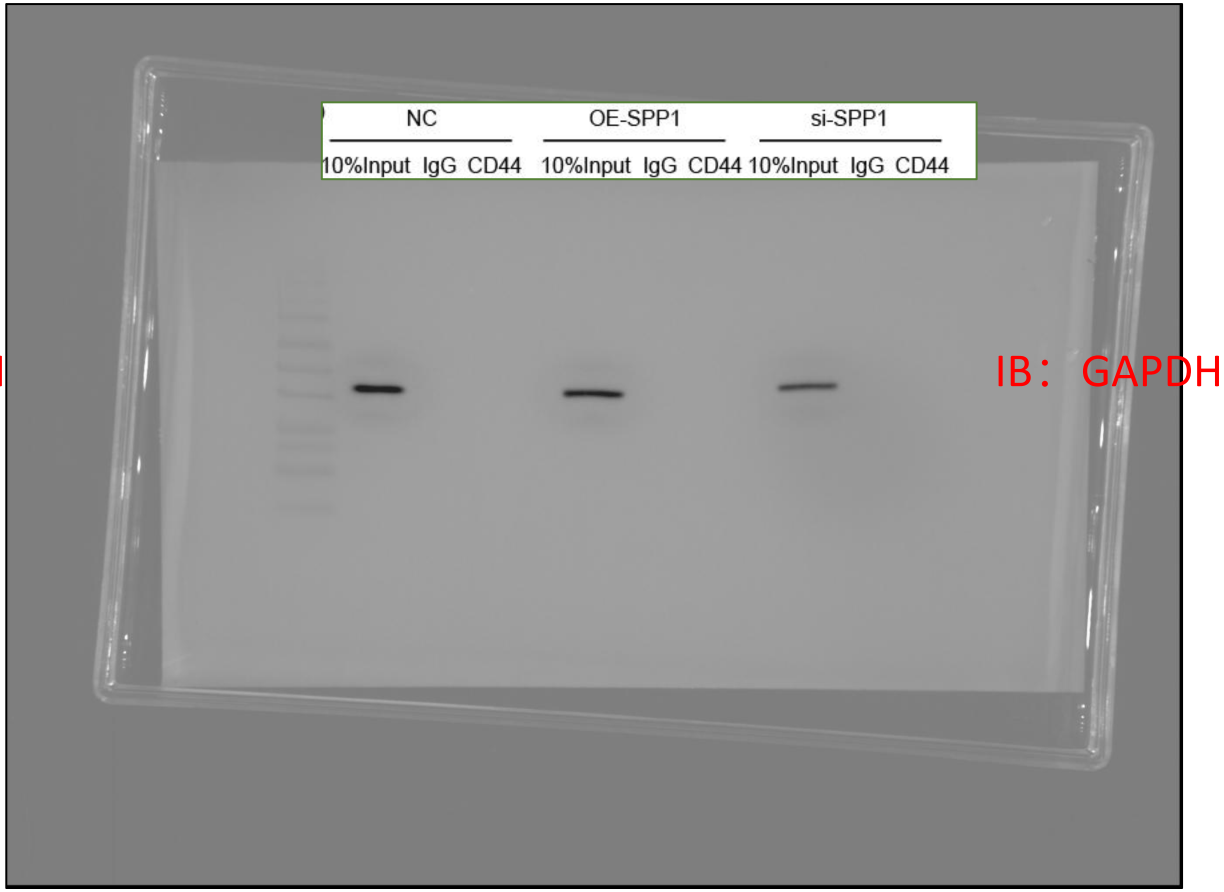

With marker

Fig 5A

TNFAIP6 promoter (-2000,+75) probe
DNA pulldown Silver staining

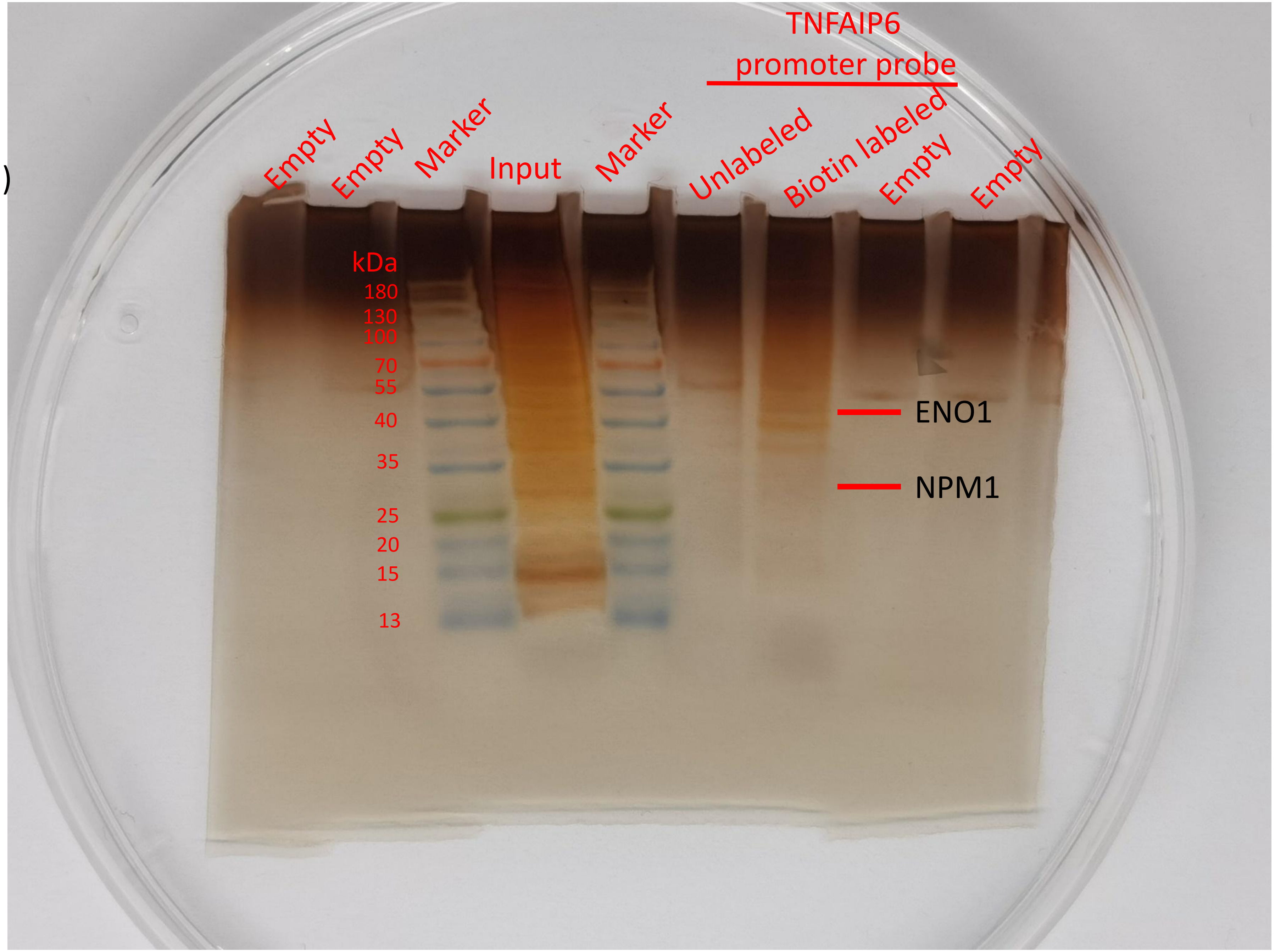

Fig 5C si NPM1 VS NC IB TNFAIP6

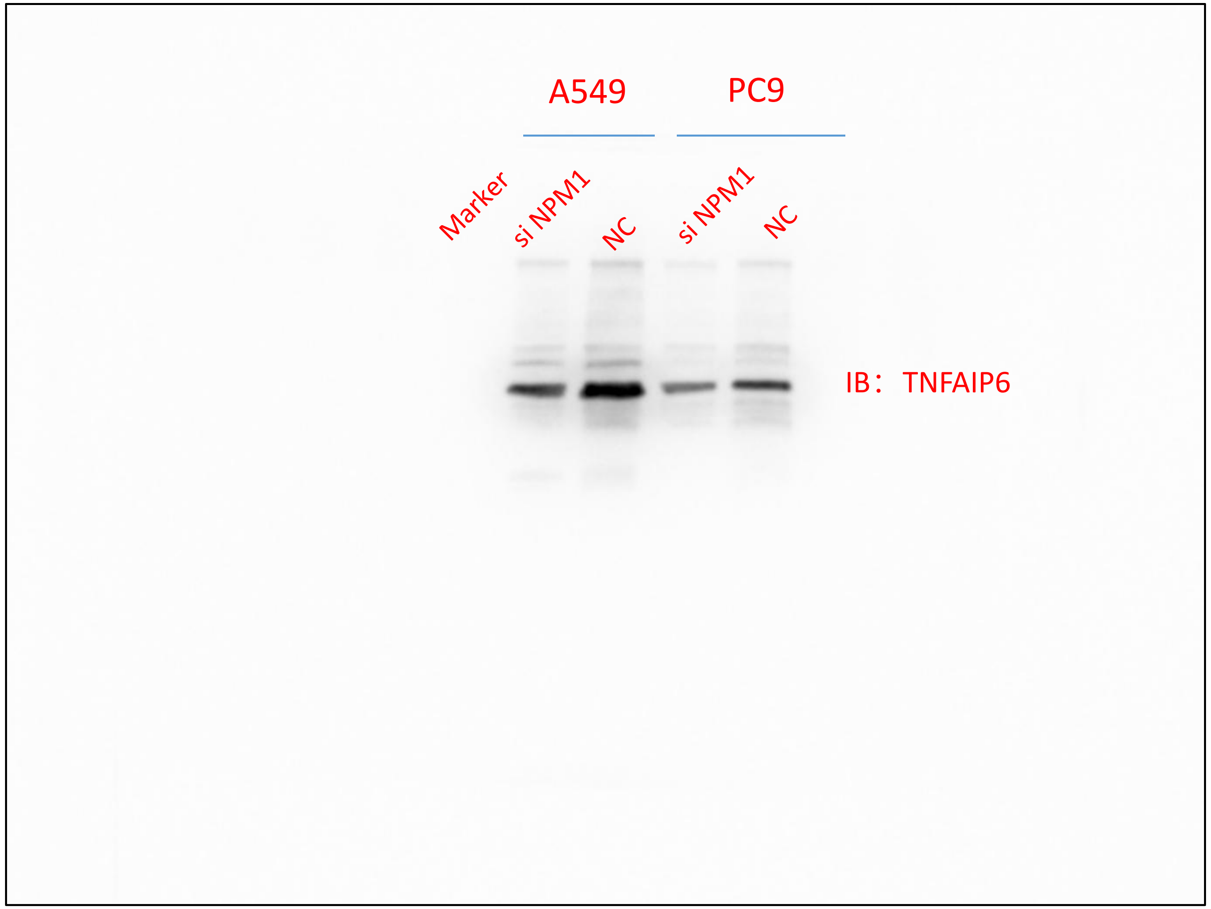

Without marker

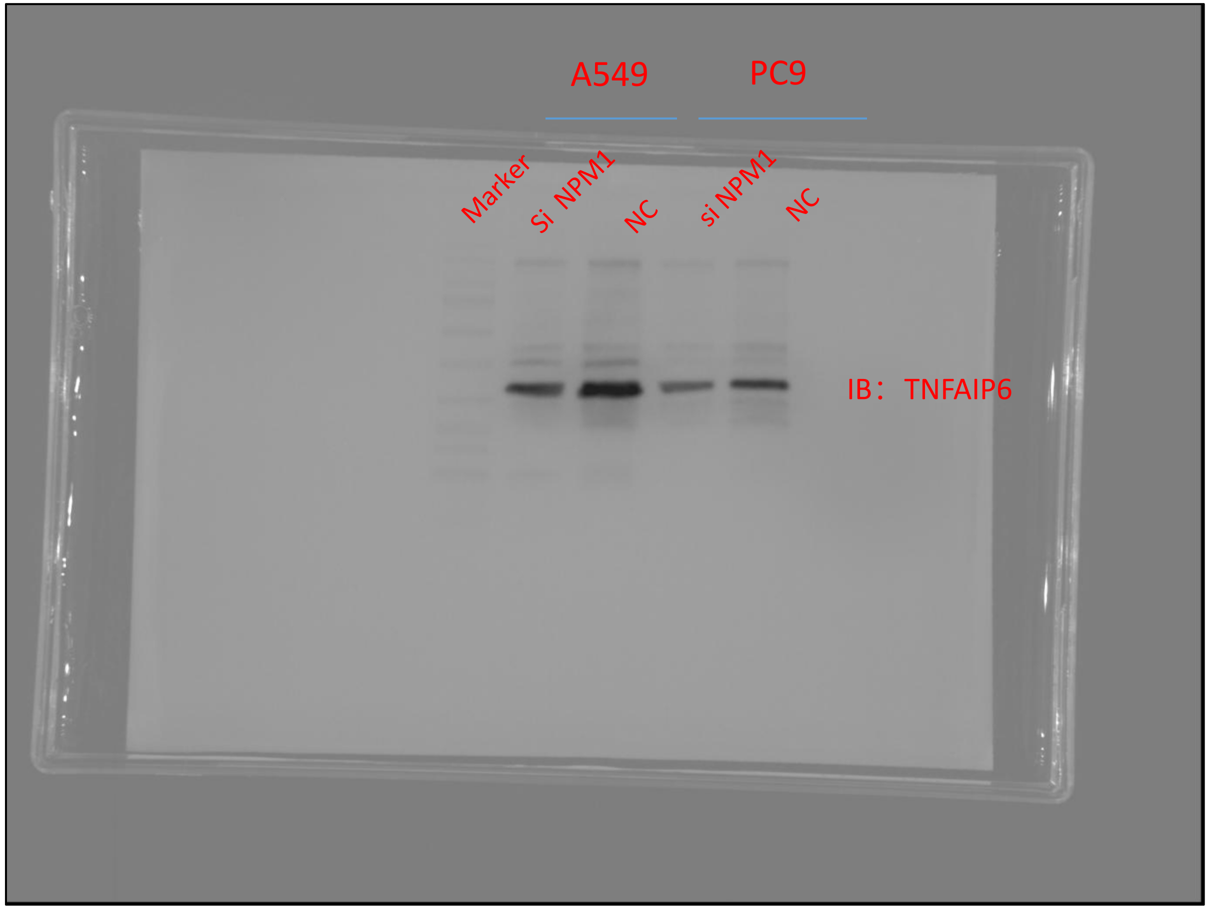

With marker

Fig 5C si NPM1 VS NC IB GAPDH

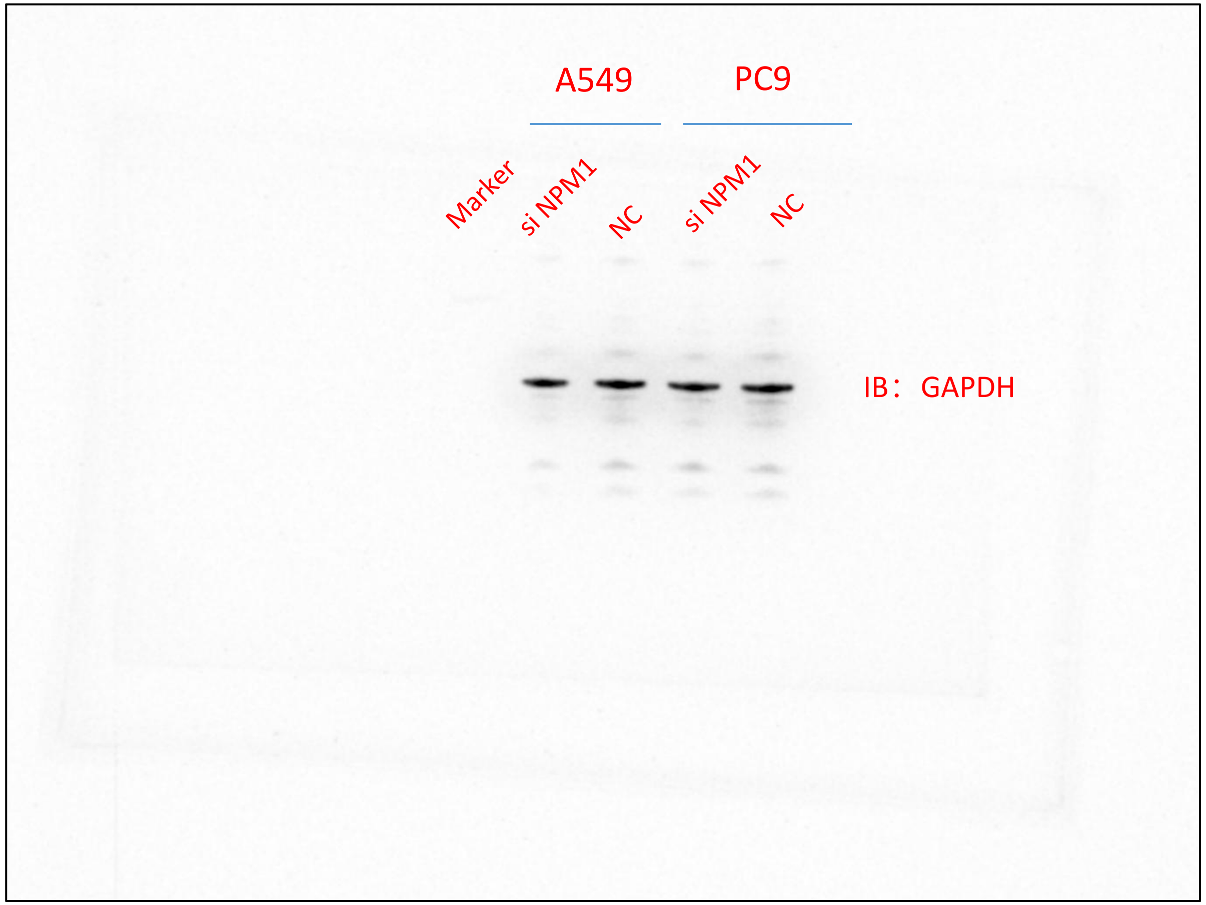

Without marker

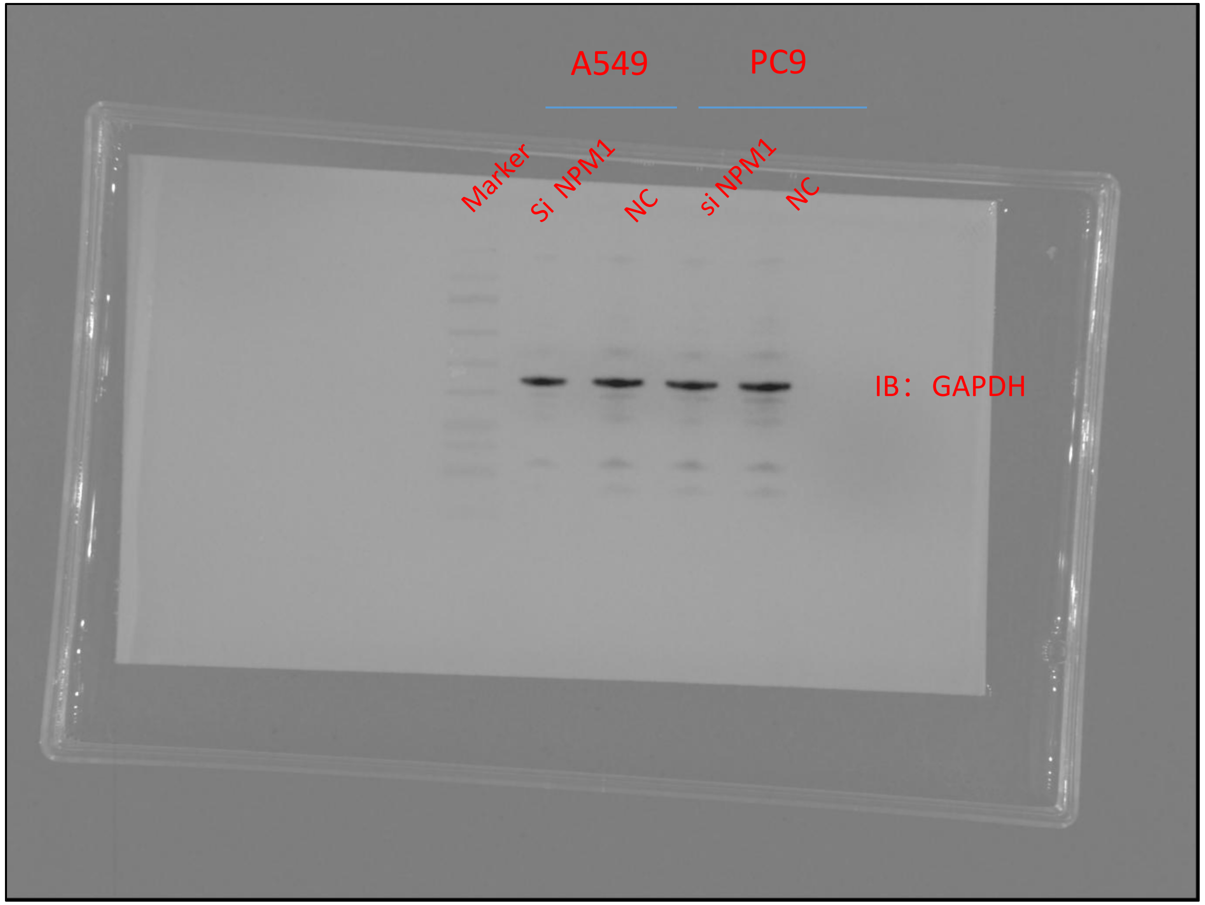

With marker

Fig 5D OE NPM1 VS NC IB TNFAIP6

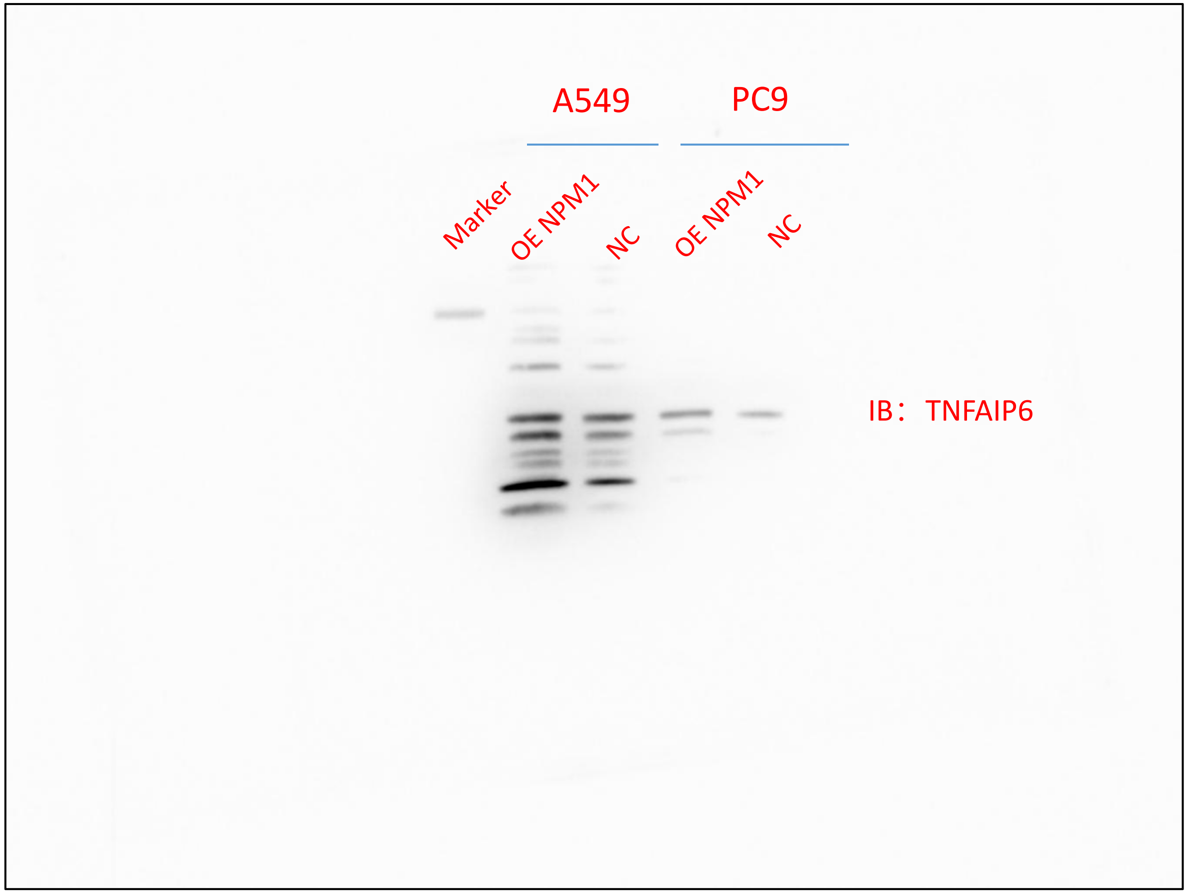

Without marker

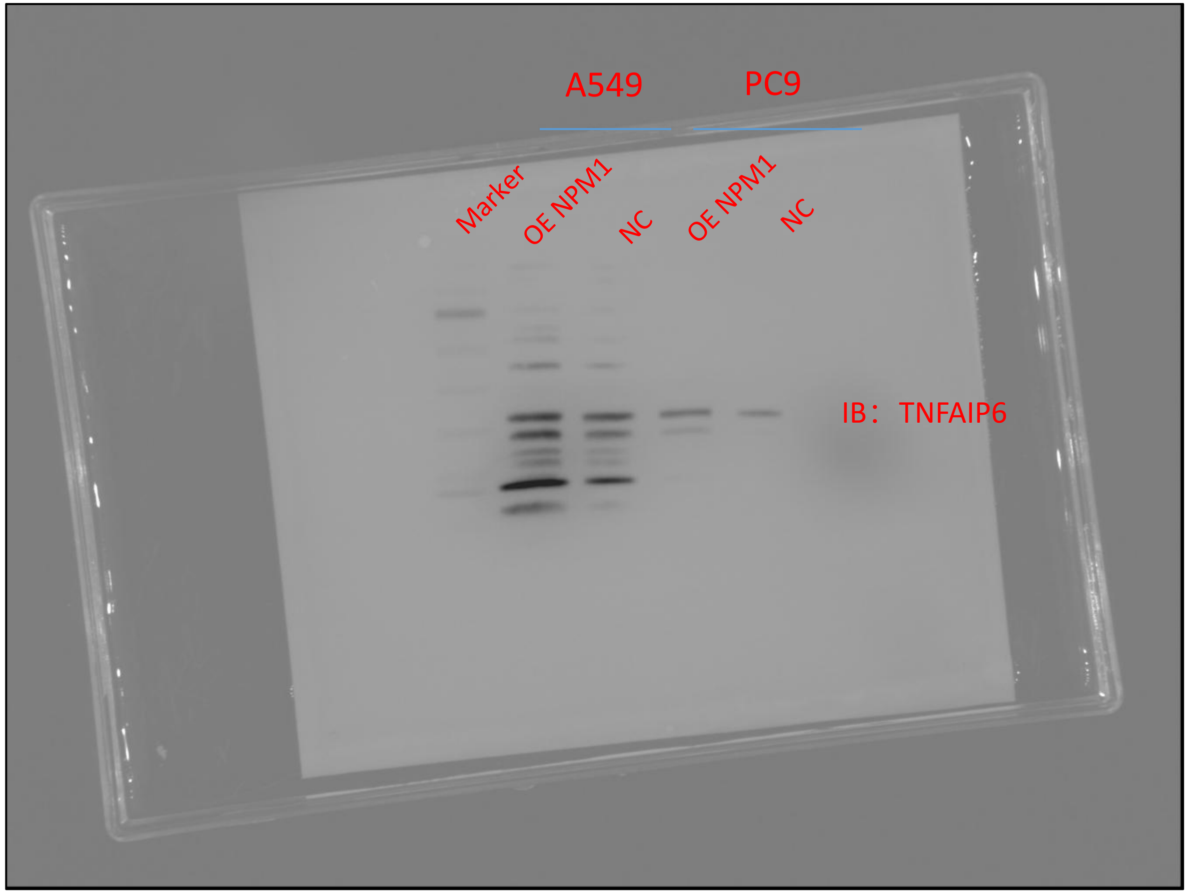

With marker

Fig 5D OE NPM1 VS NC IB GAPDH

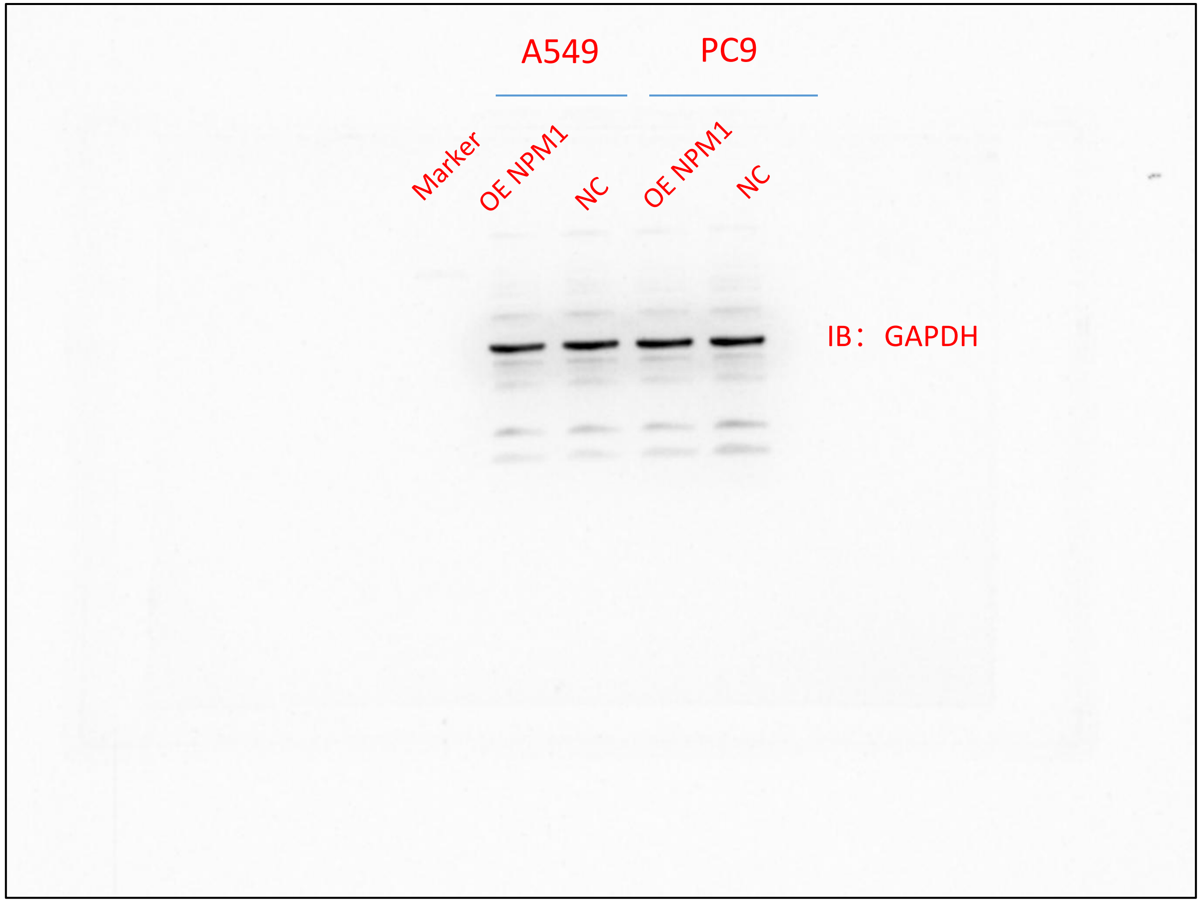

Without marker

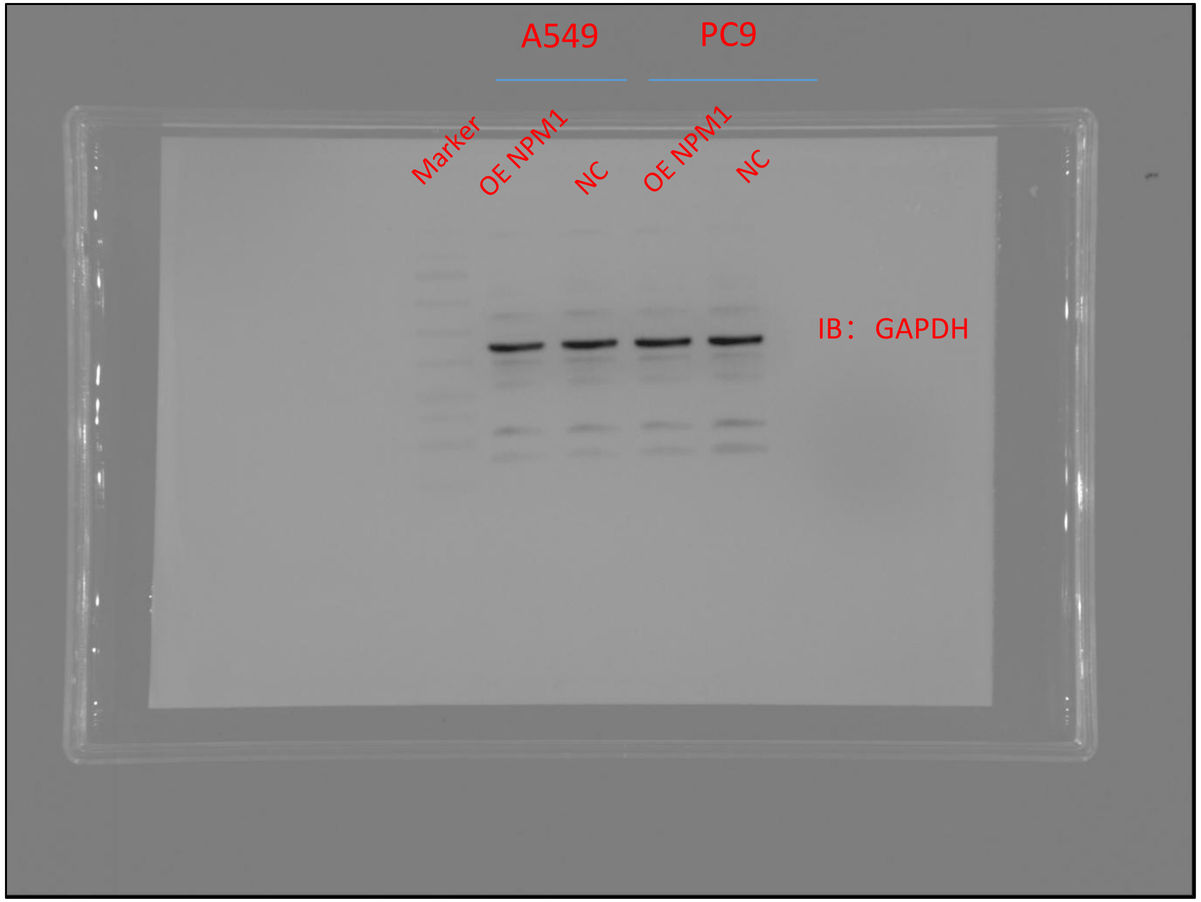

With marker

Fig 6A Probes IB NPM1

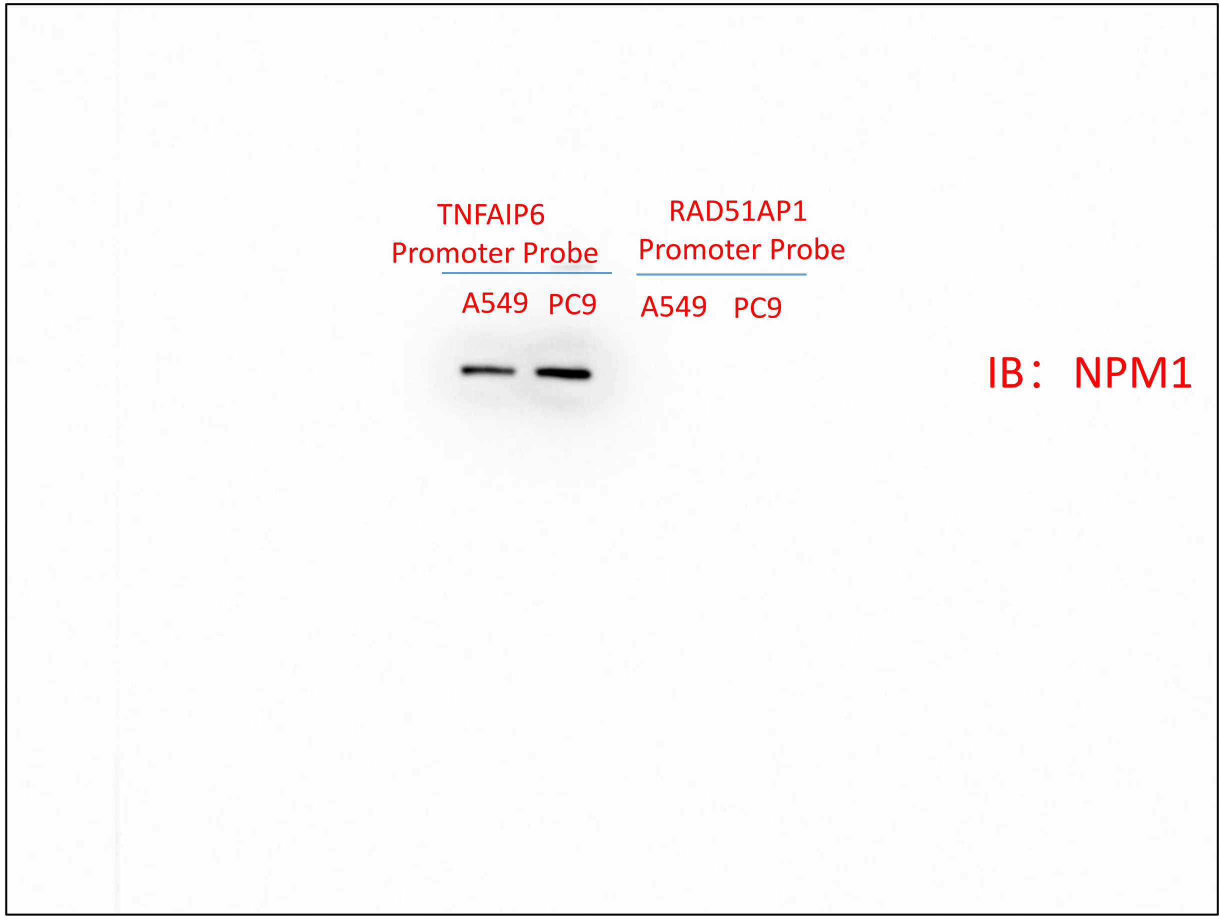

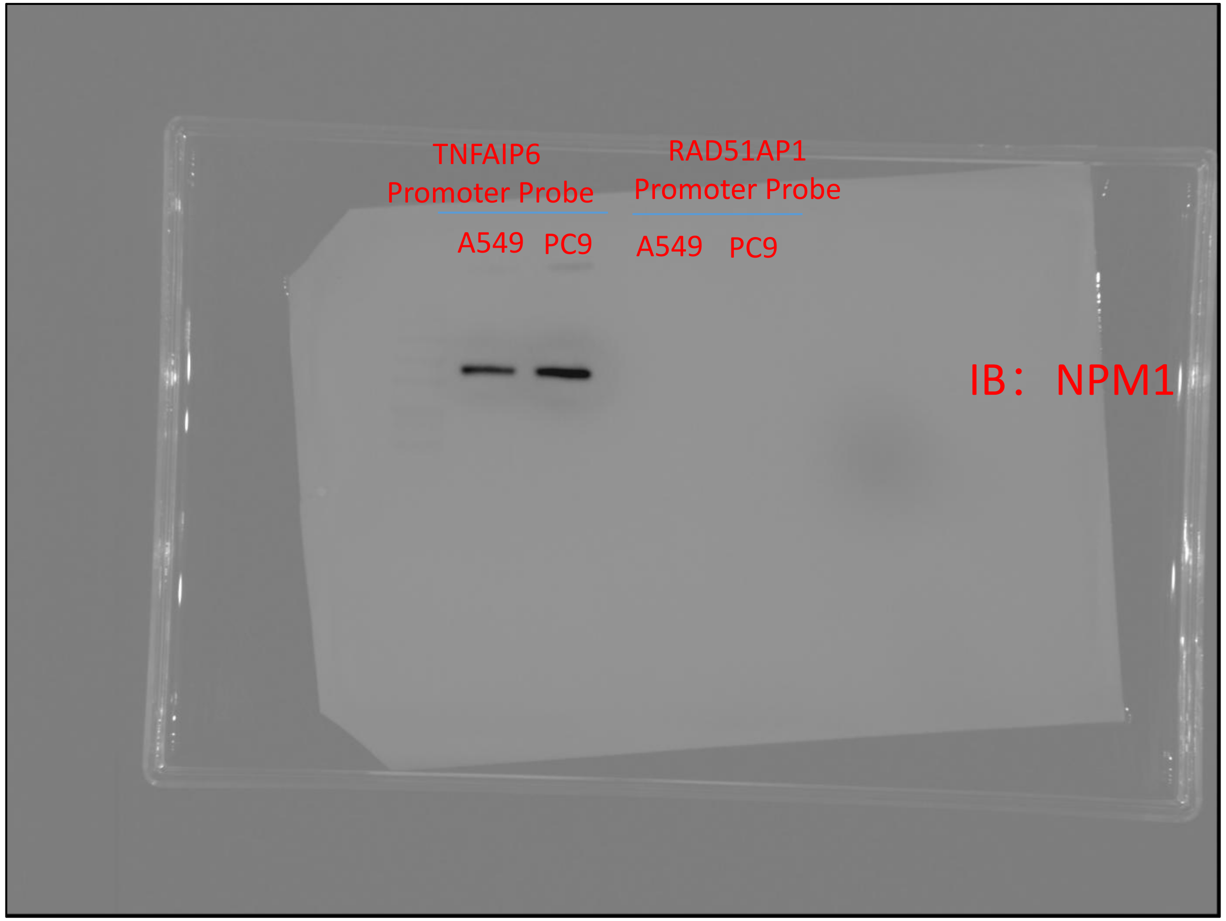

Fig 6A input IB NPM1

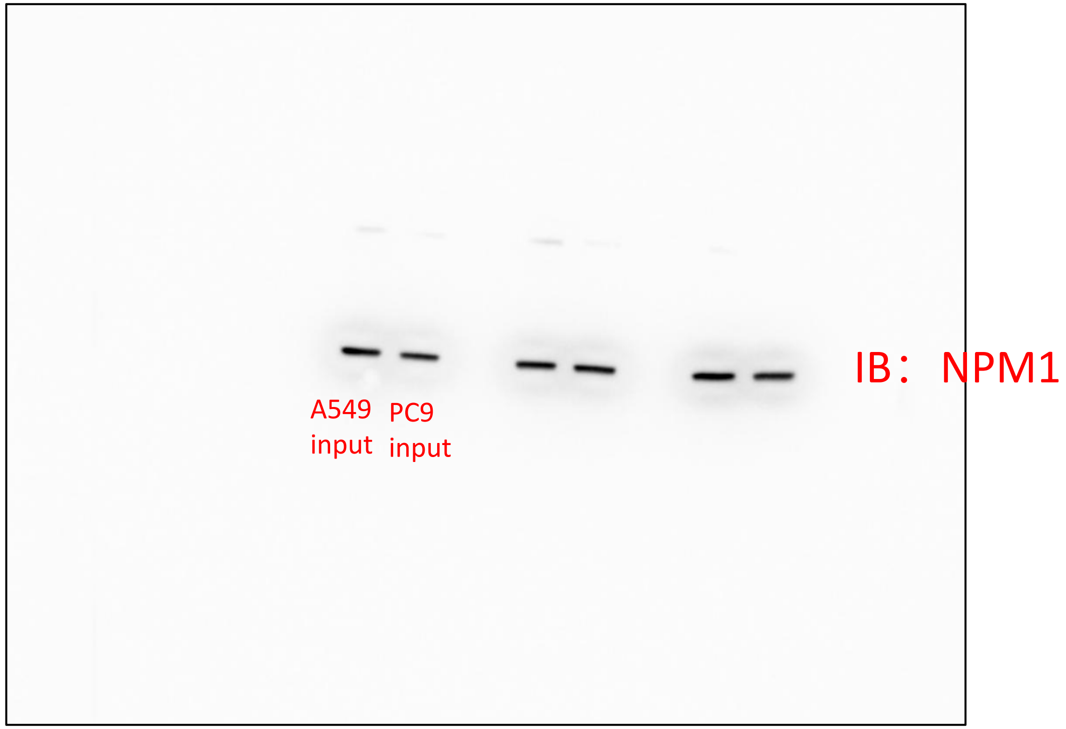

Without marker

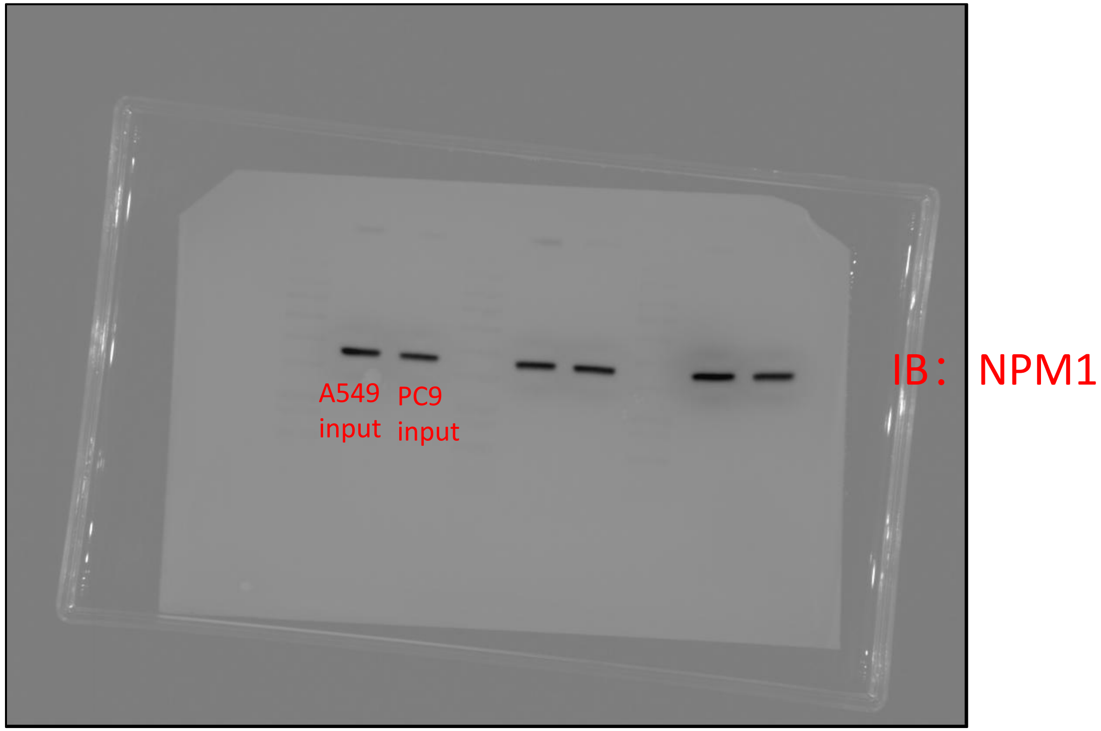

With marker

Fig 6A input IB Histone H3

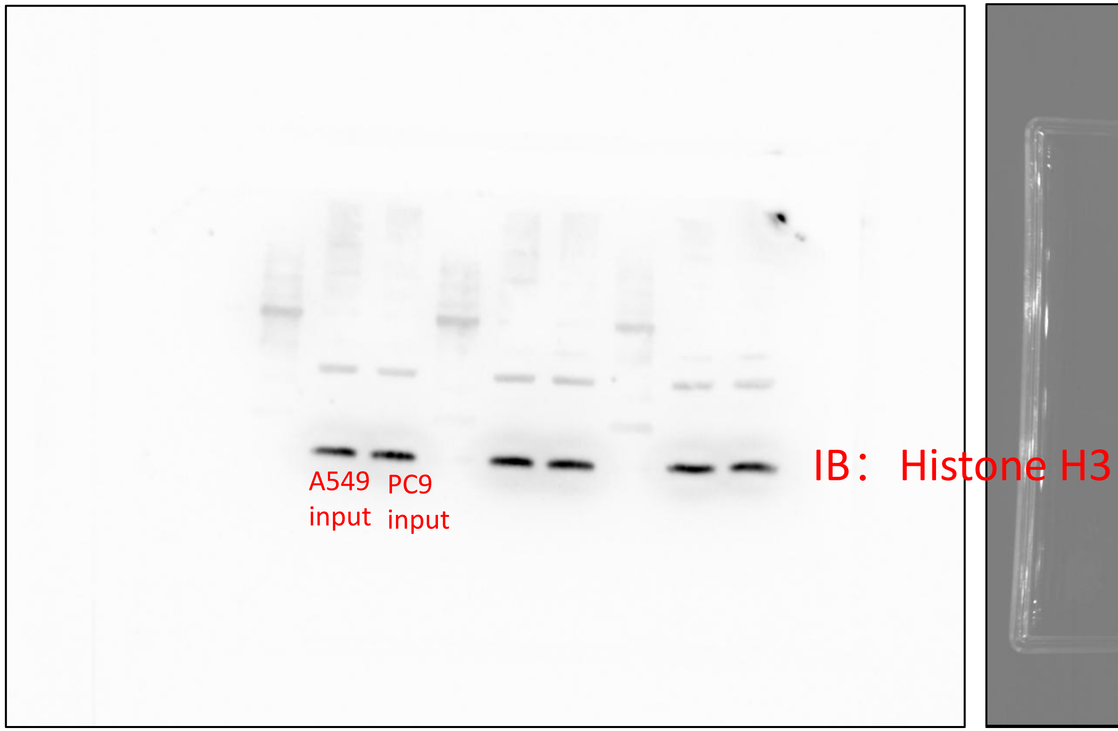

Without marker

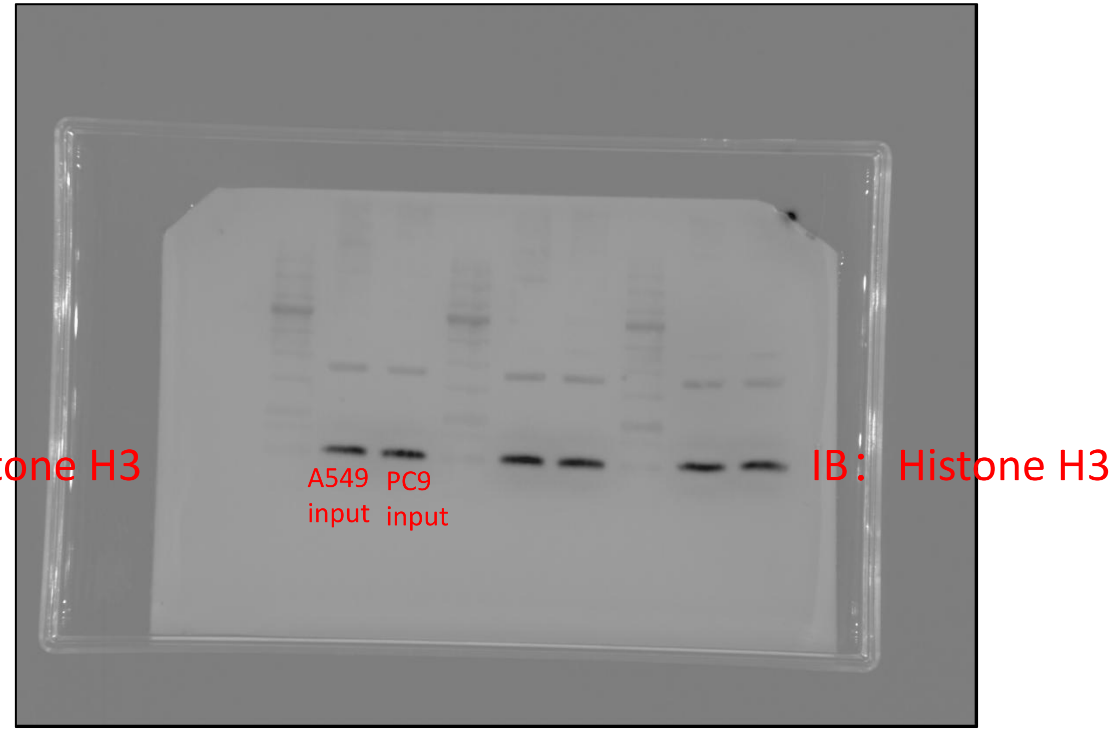

With marker

Fig 6B Probes IB NPM1

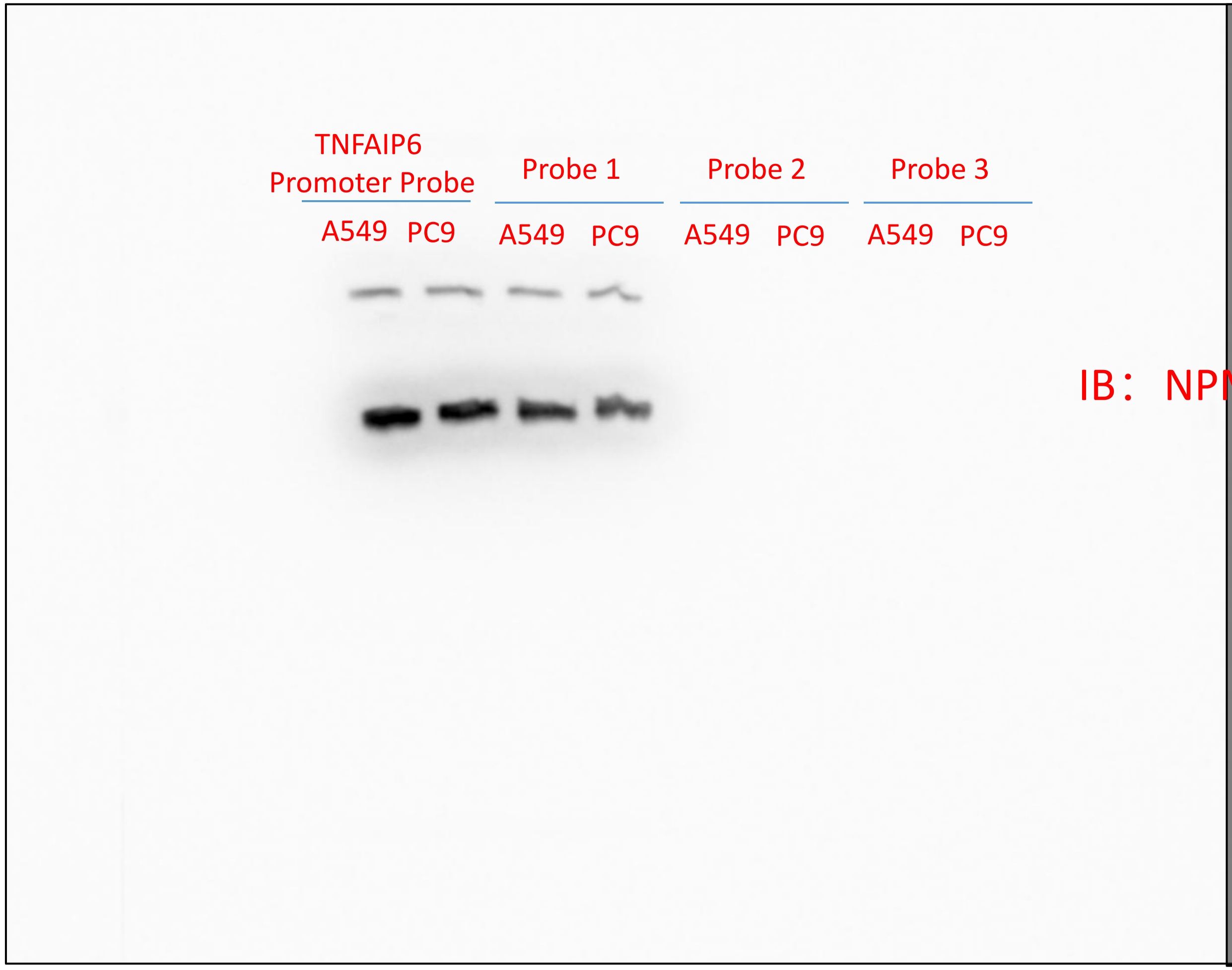

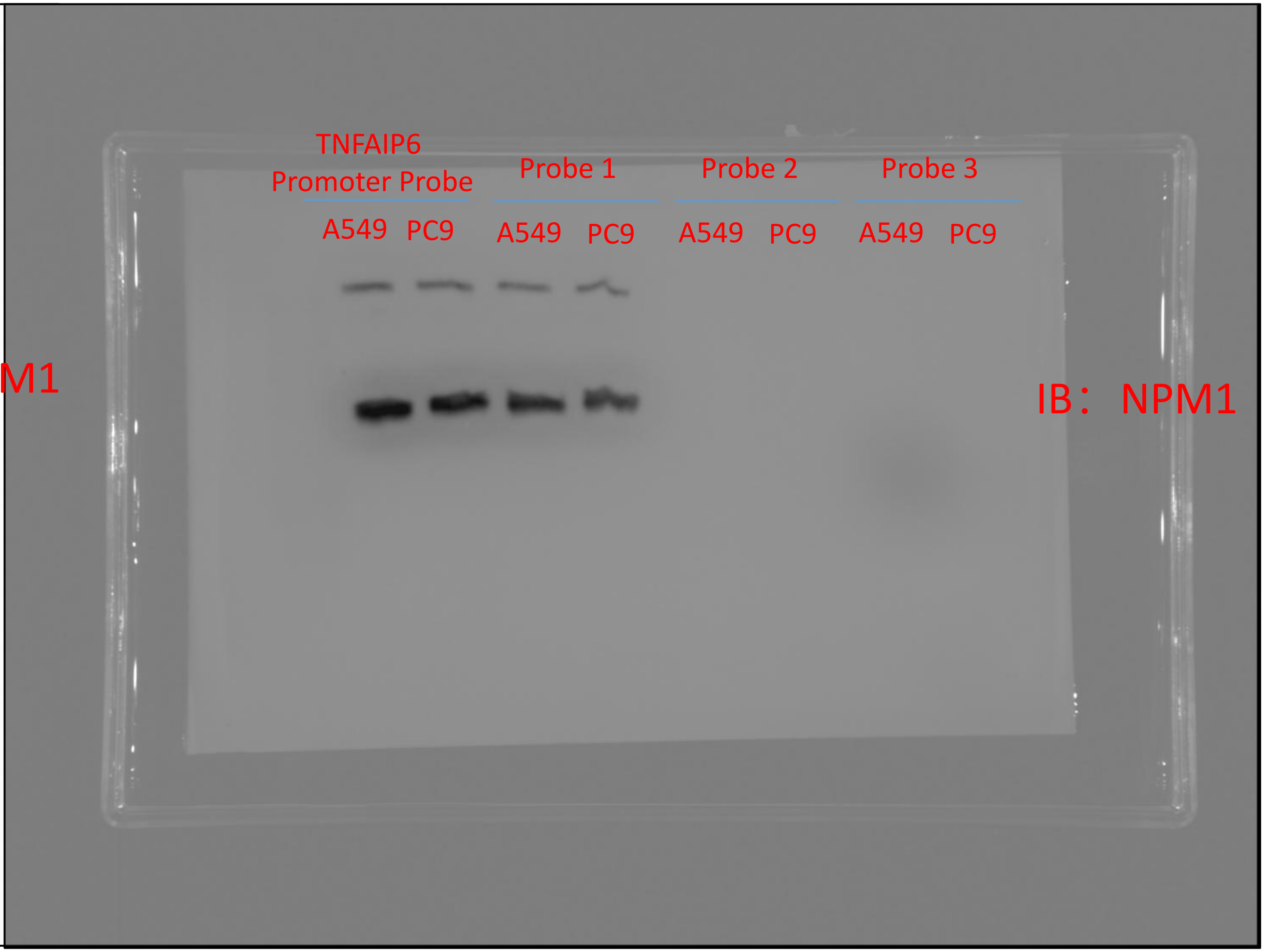

Fig 6B input IB NPM1

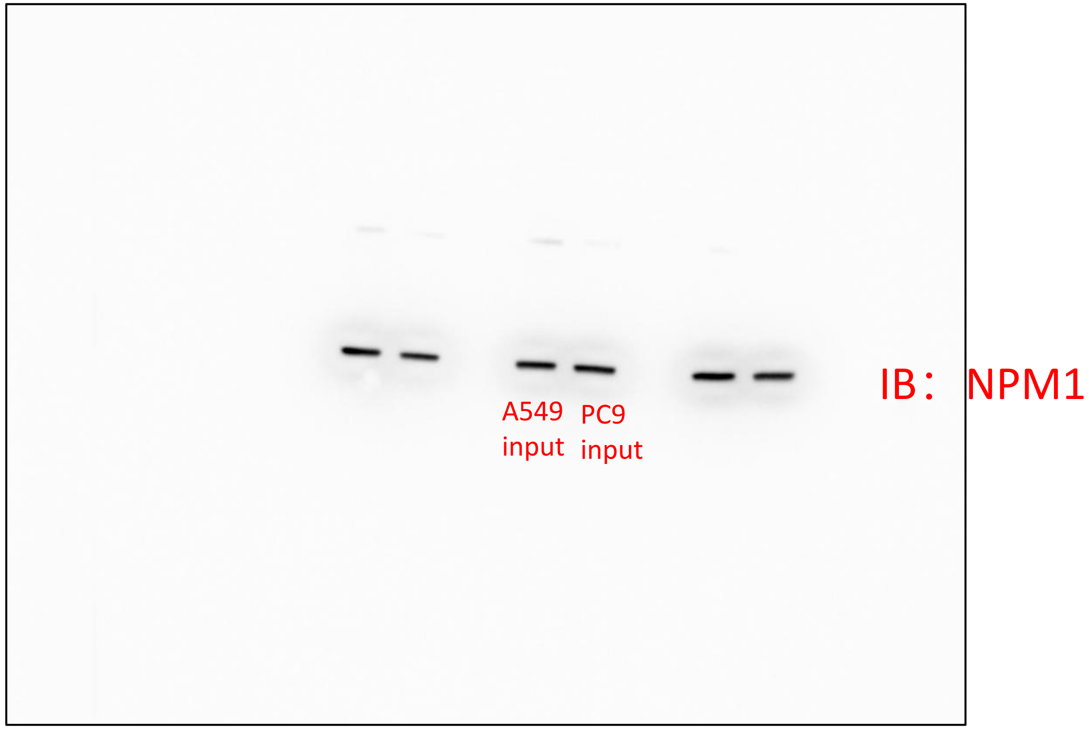

Without marker

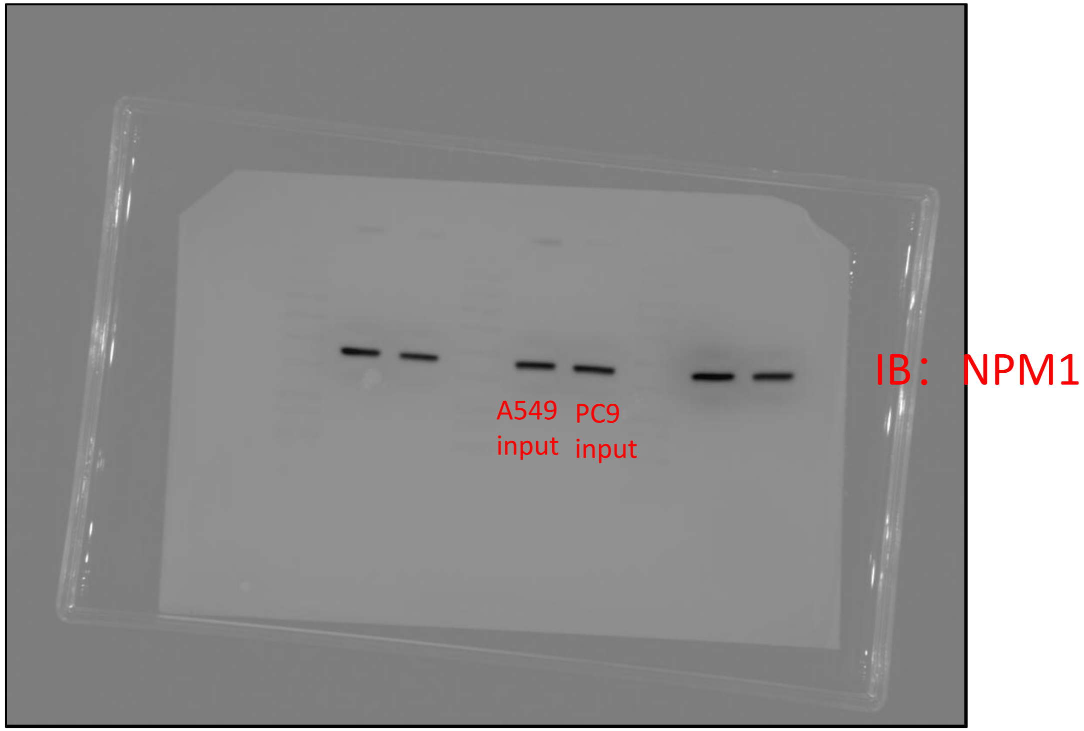

With marker

Fig 6B input IB Histone H3

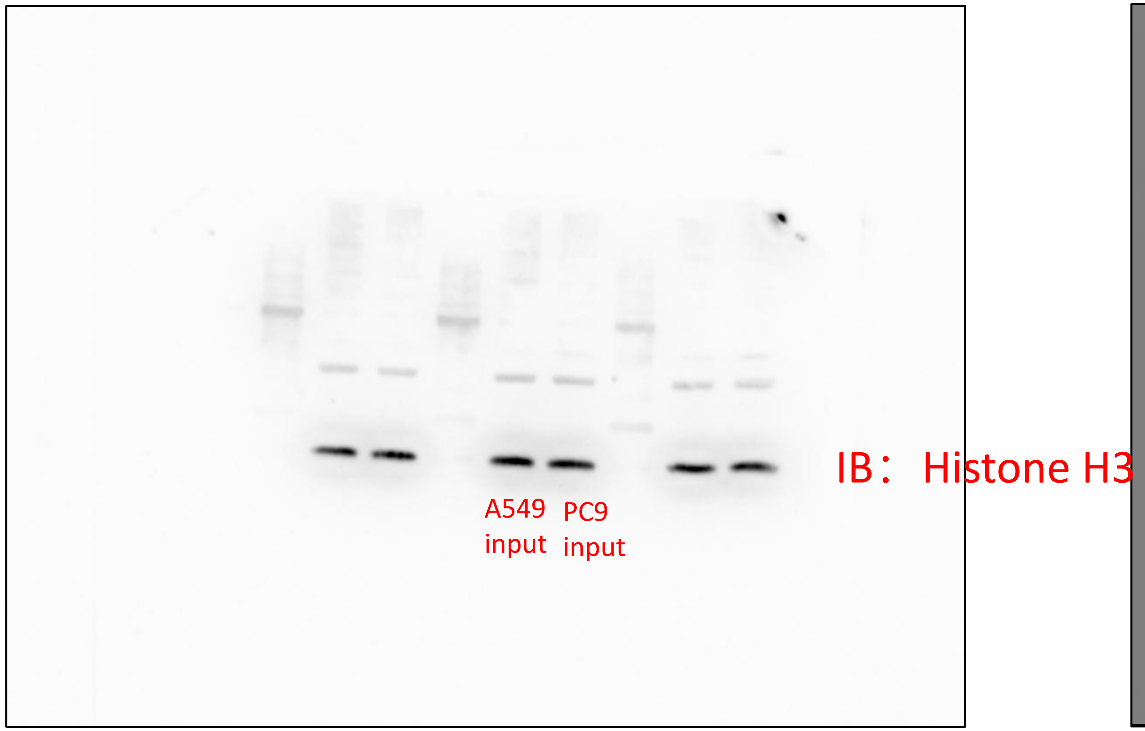

Without marker

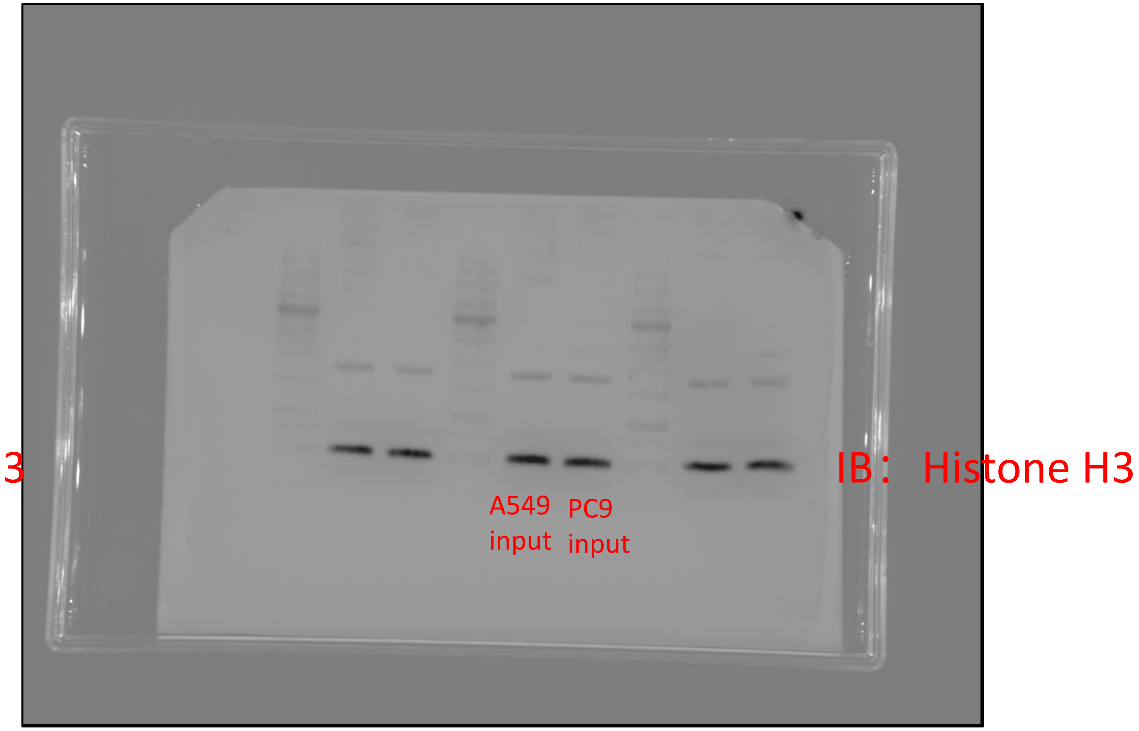

With marker

Fig 6C Probes IB NPM1

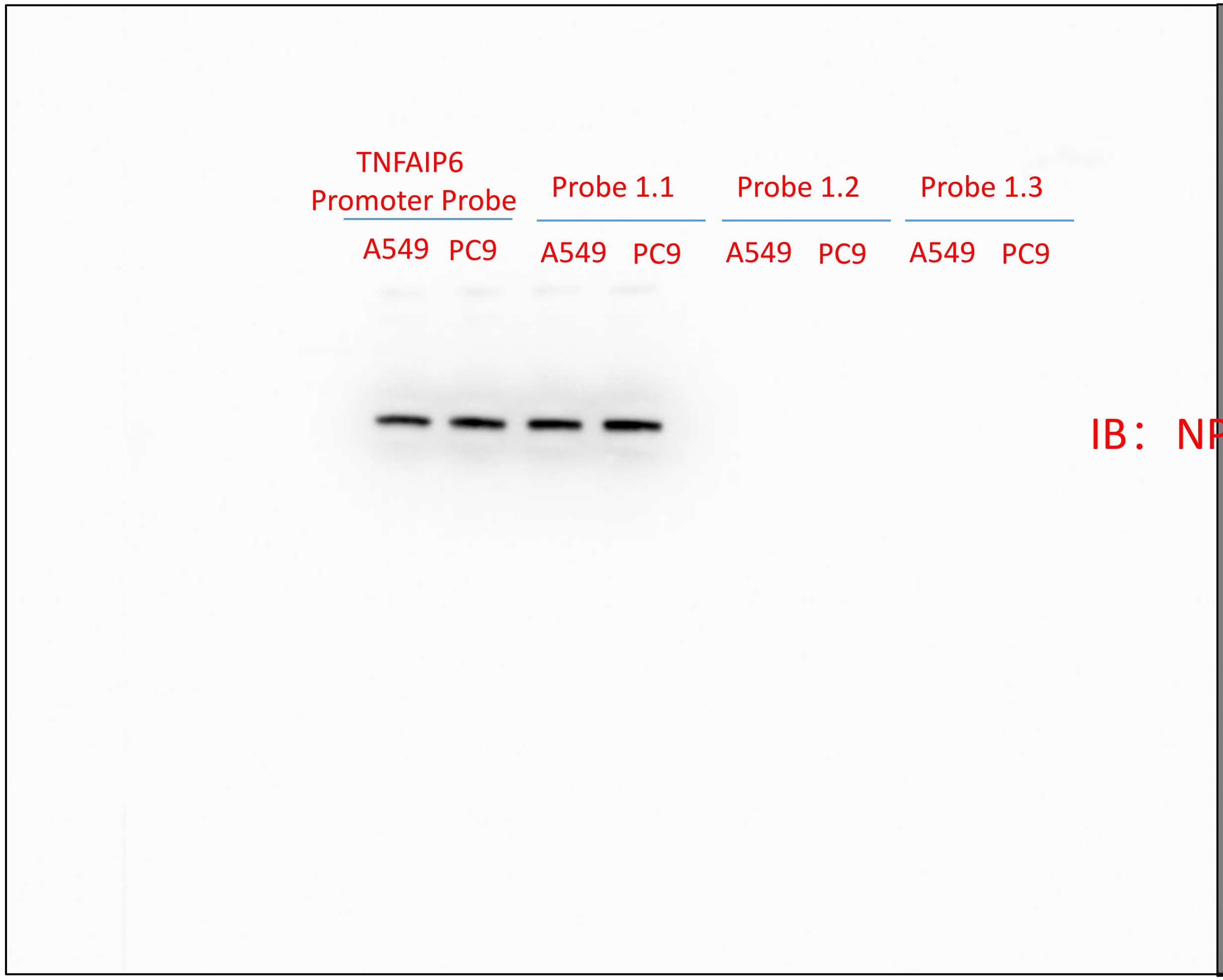

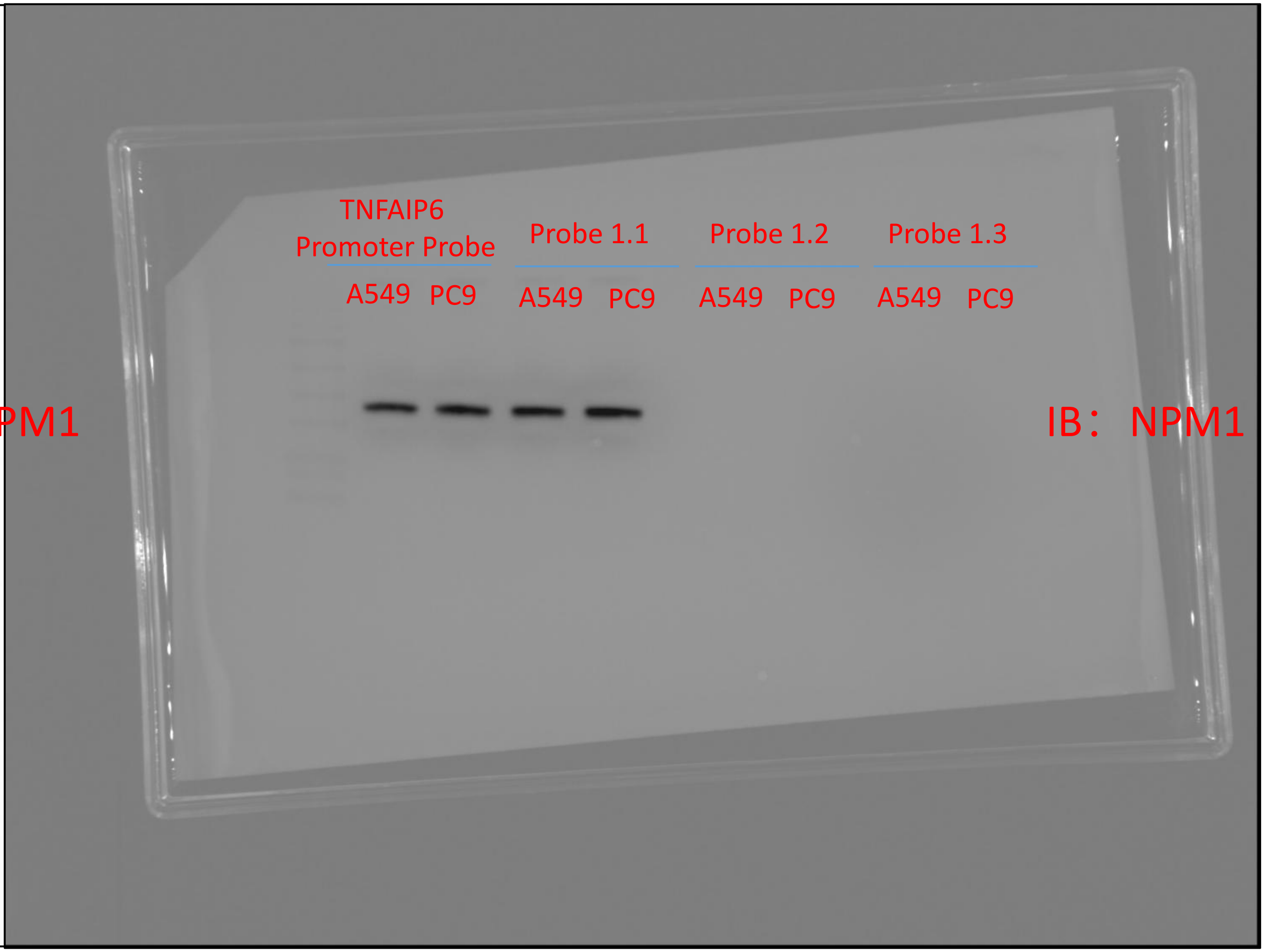

Fig 6C input IB NPM1

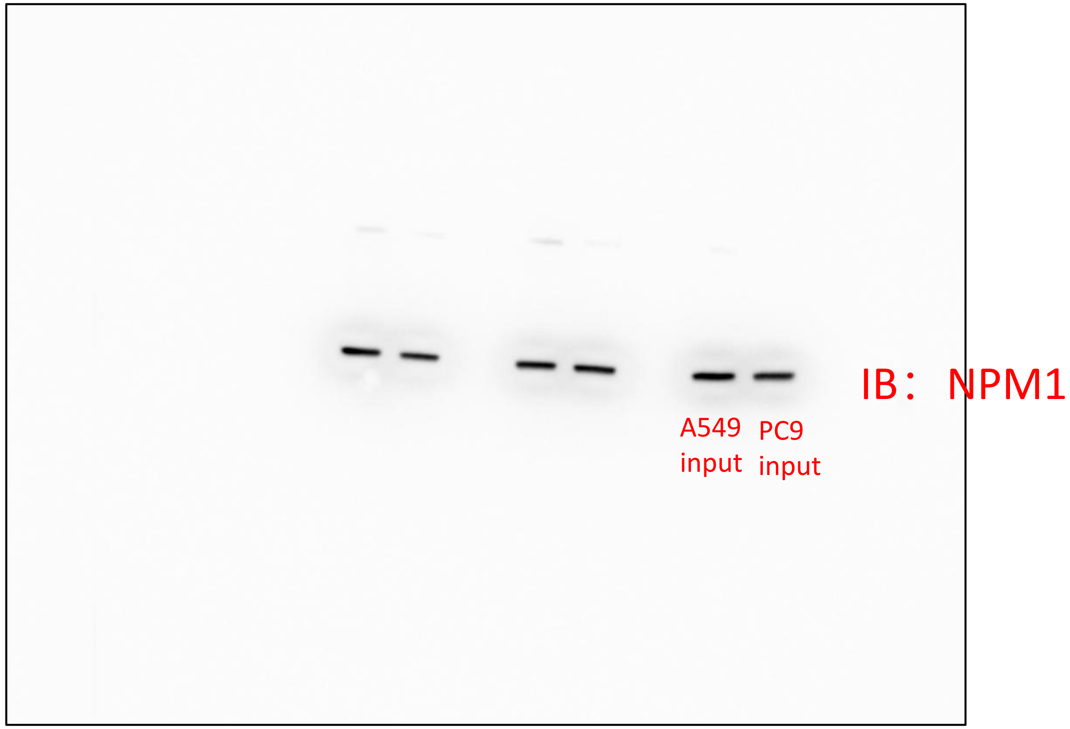

Without marker

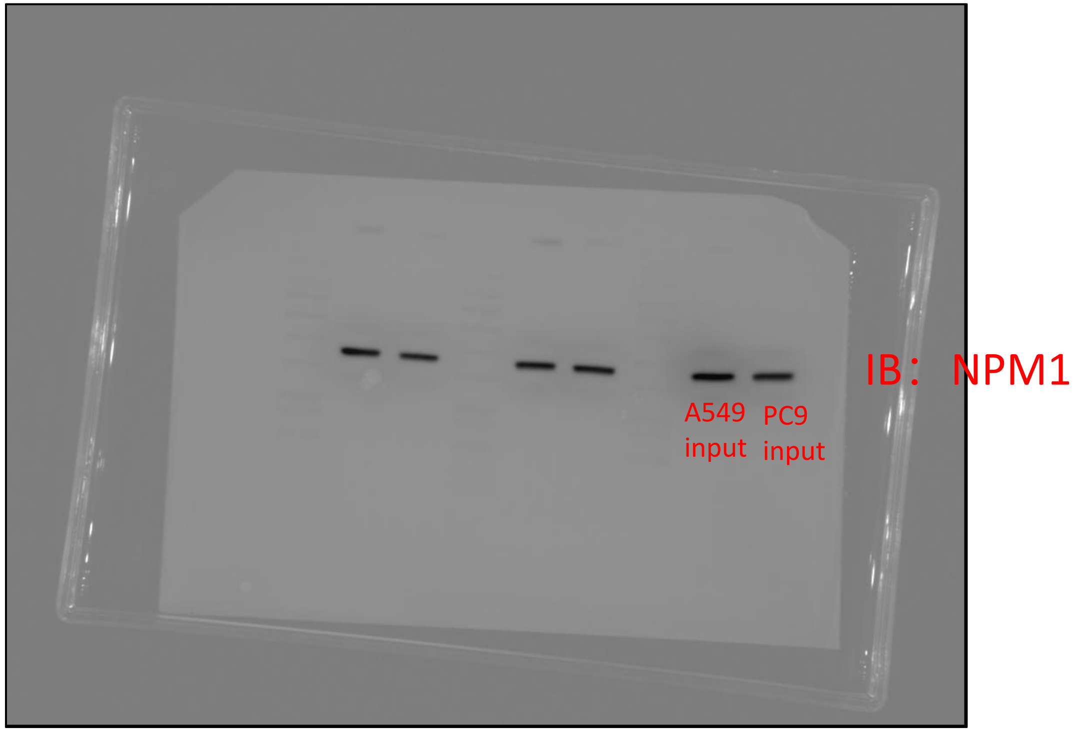

With marker

Fig 6C input IB Histone H3

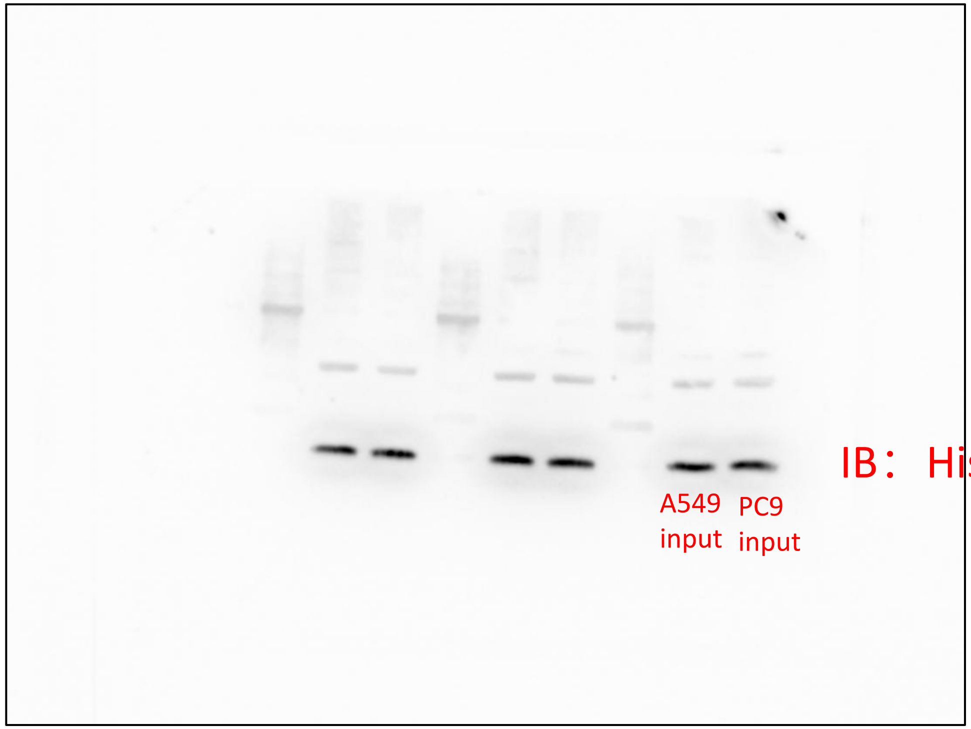

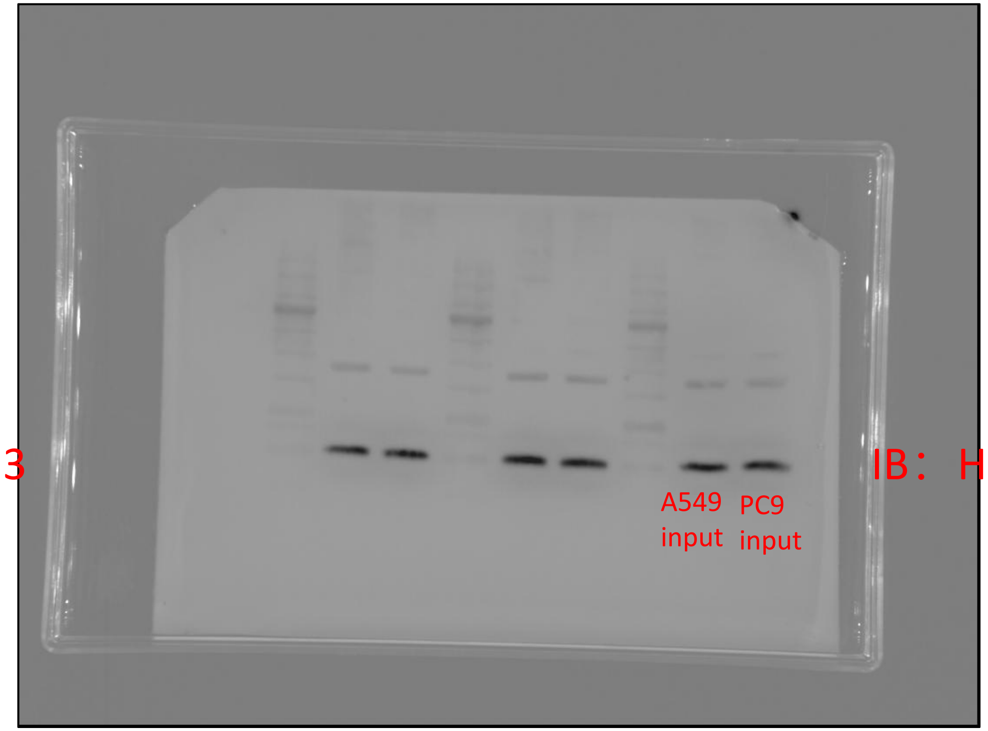

Supplementary Figure 4B
IB TNFAIP6

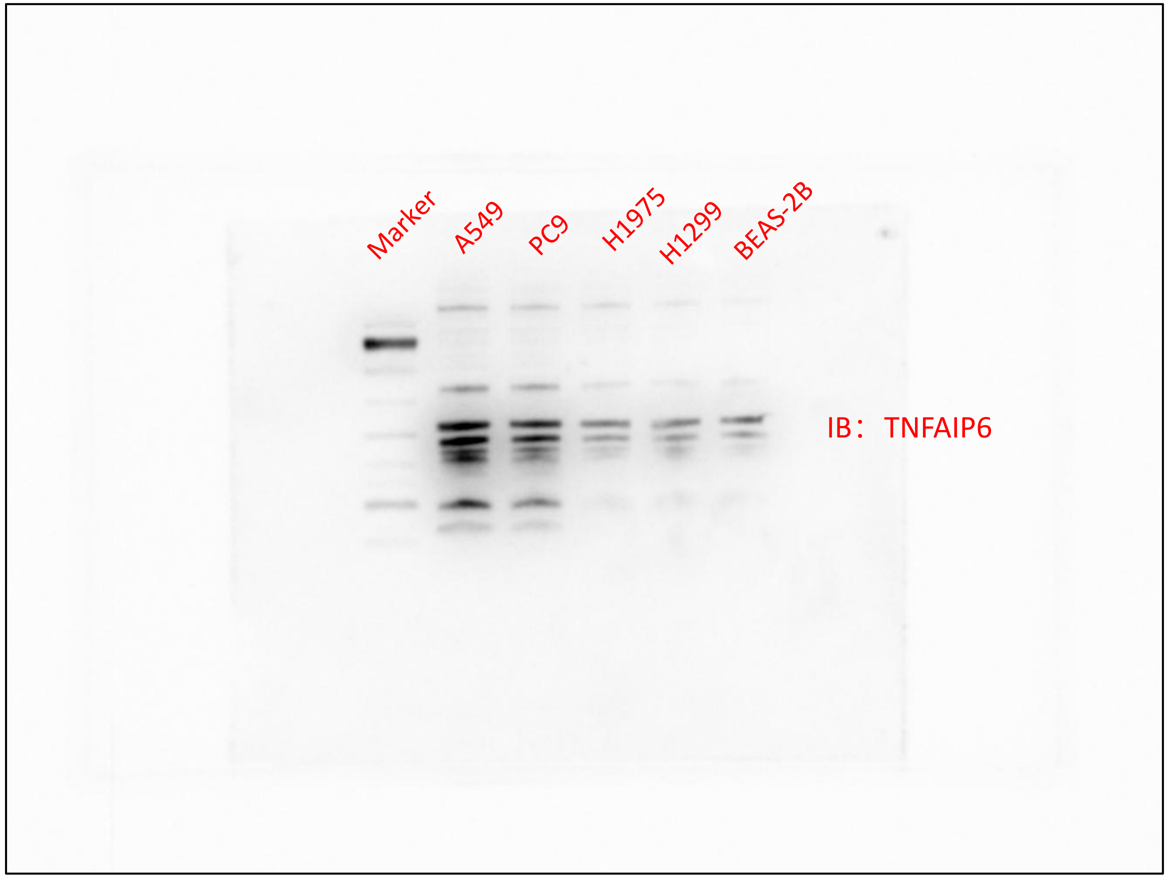

Without marker

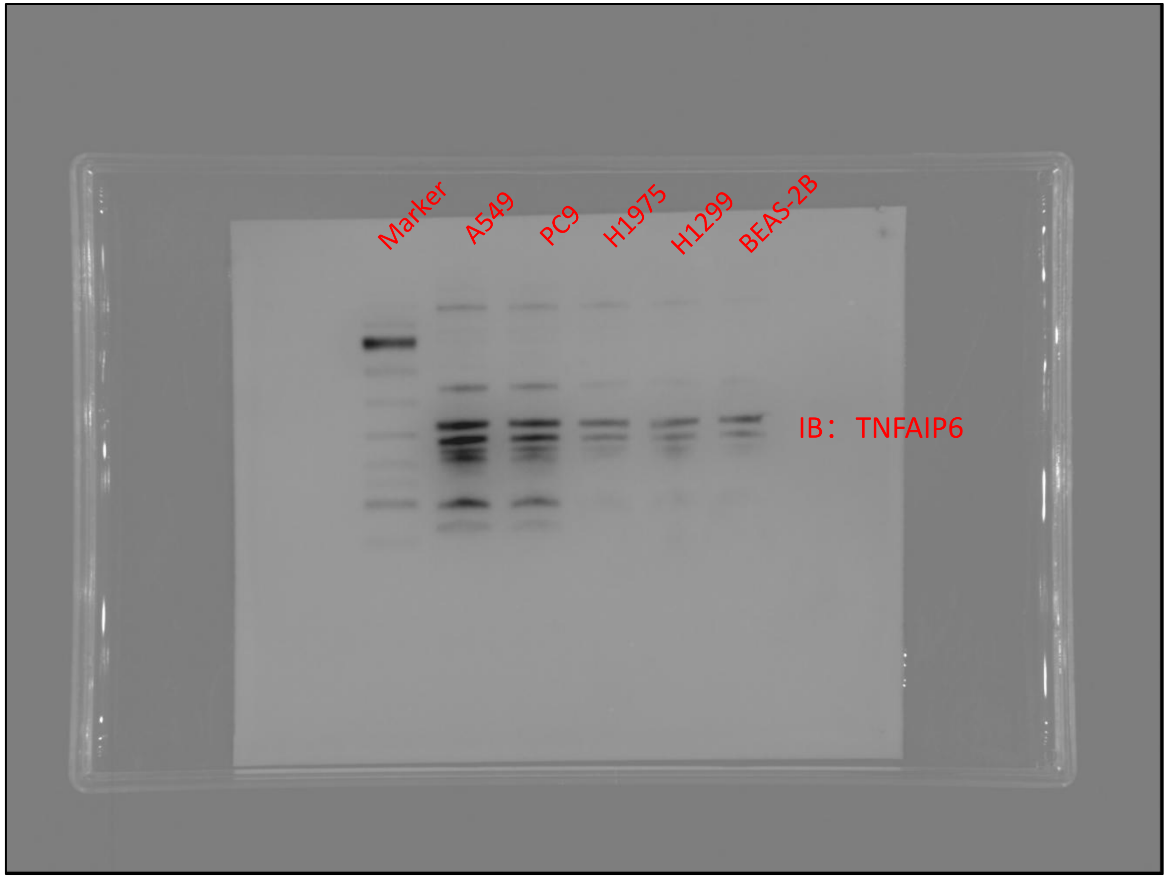

With marker

Supplementary Figure 4B
IB GAPDH

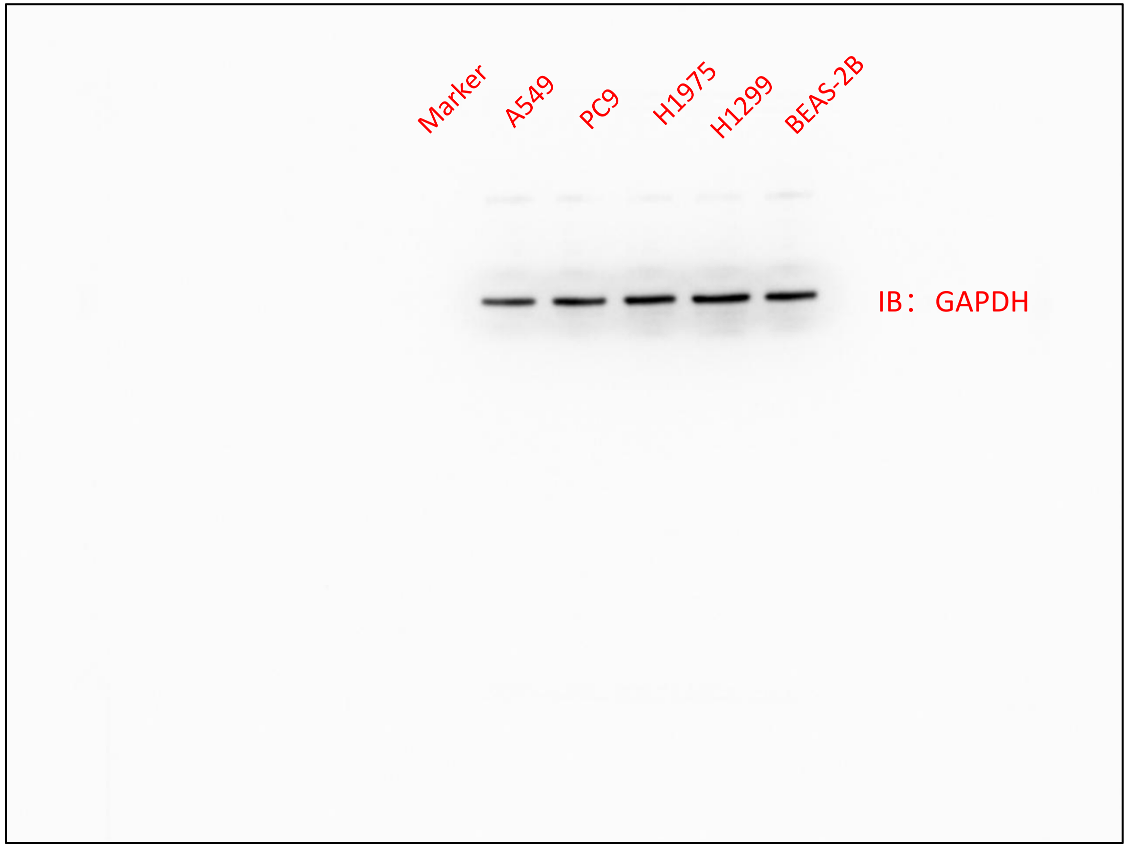

Without marker

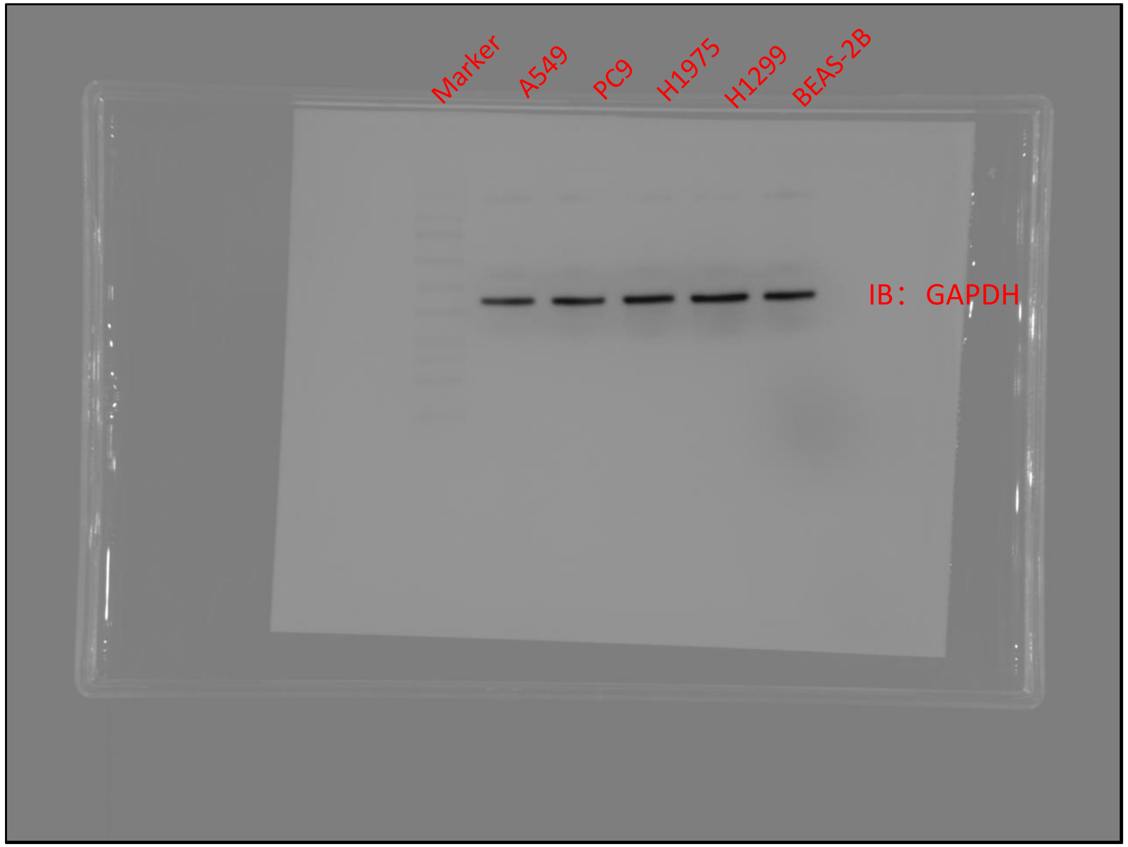

With marker

Supplementary Figure 4D
IB TNFAIP6

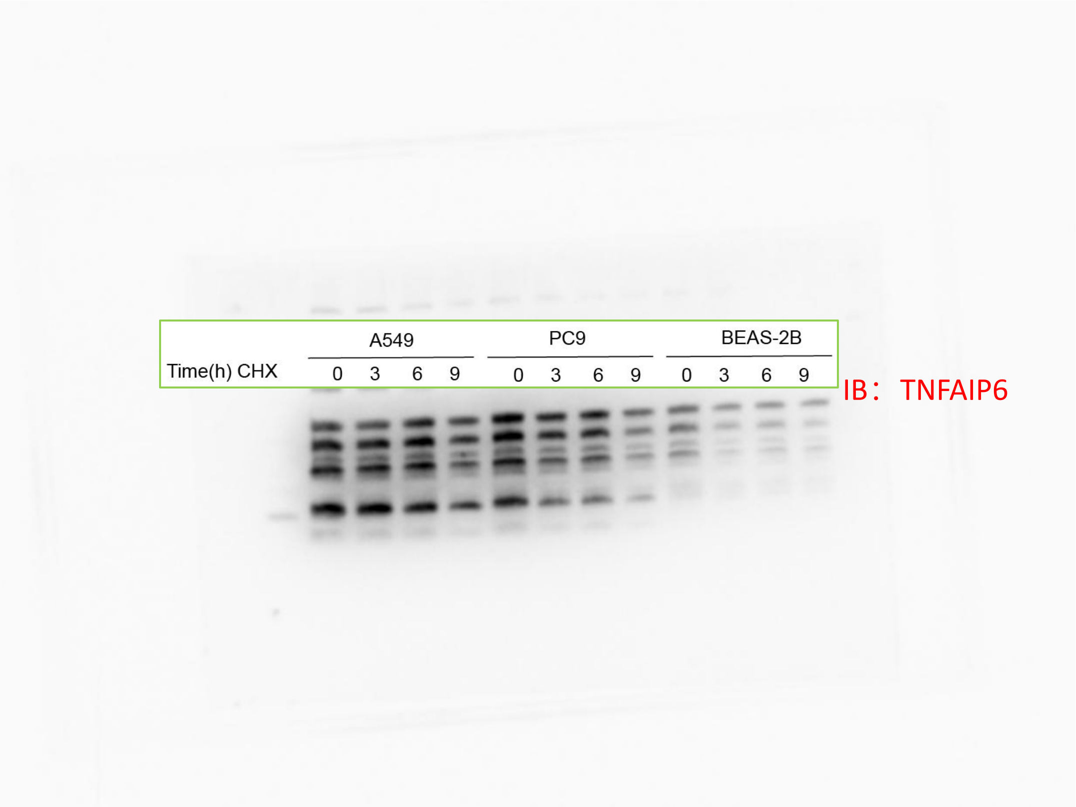

Without marker

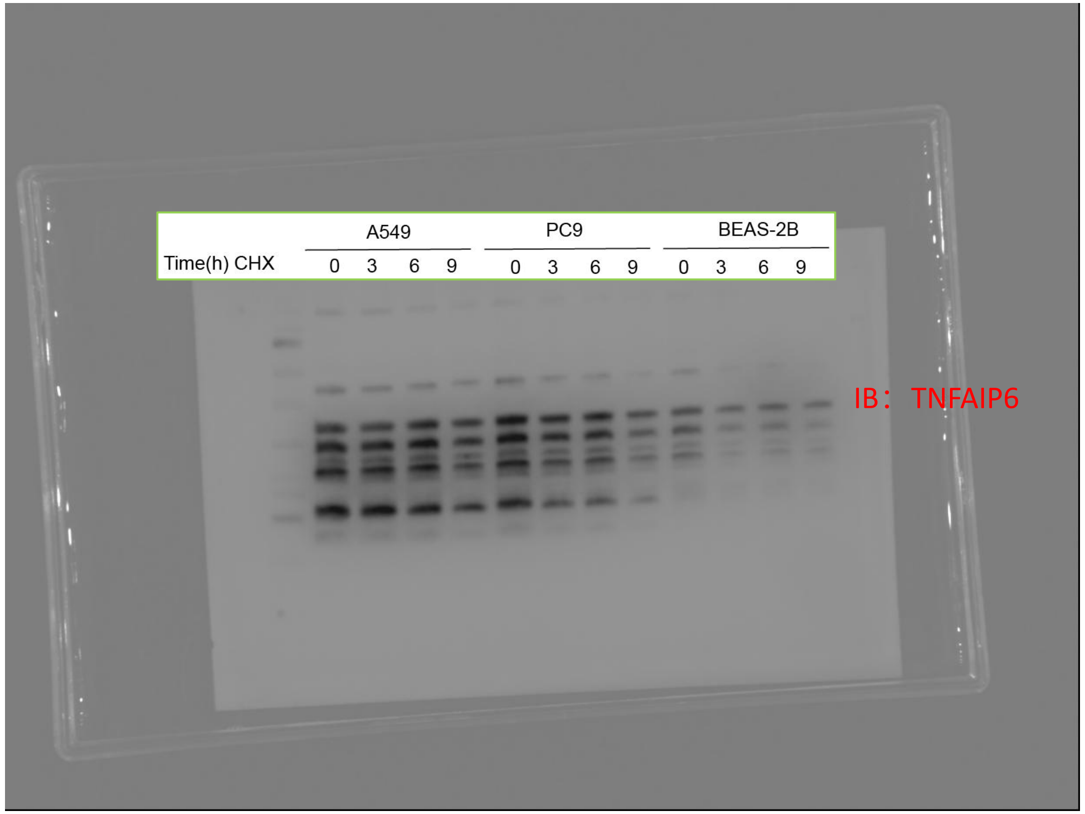

With marker

Supplementary Figure 4D
IB GAPDH

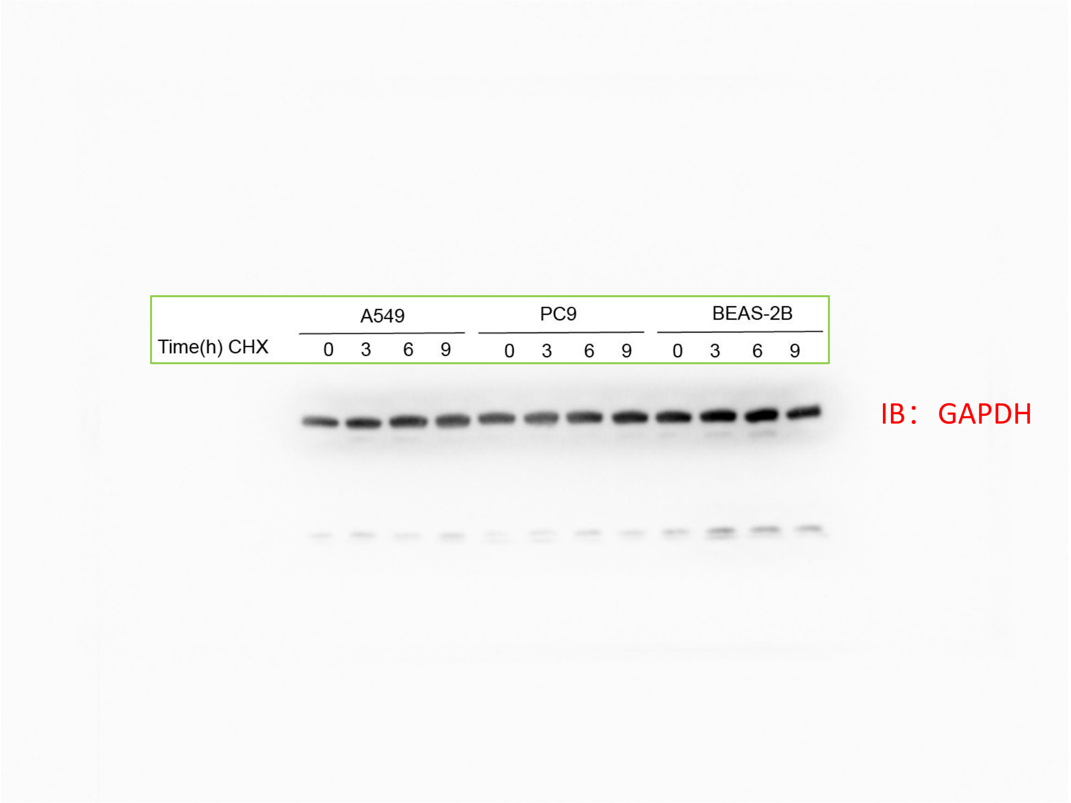

Without marker

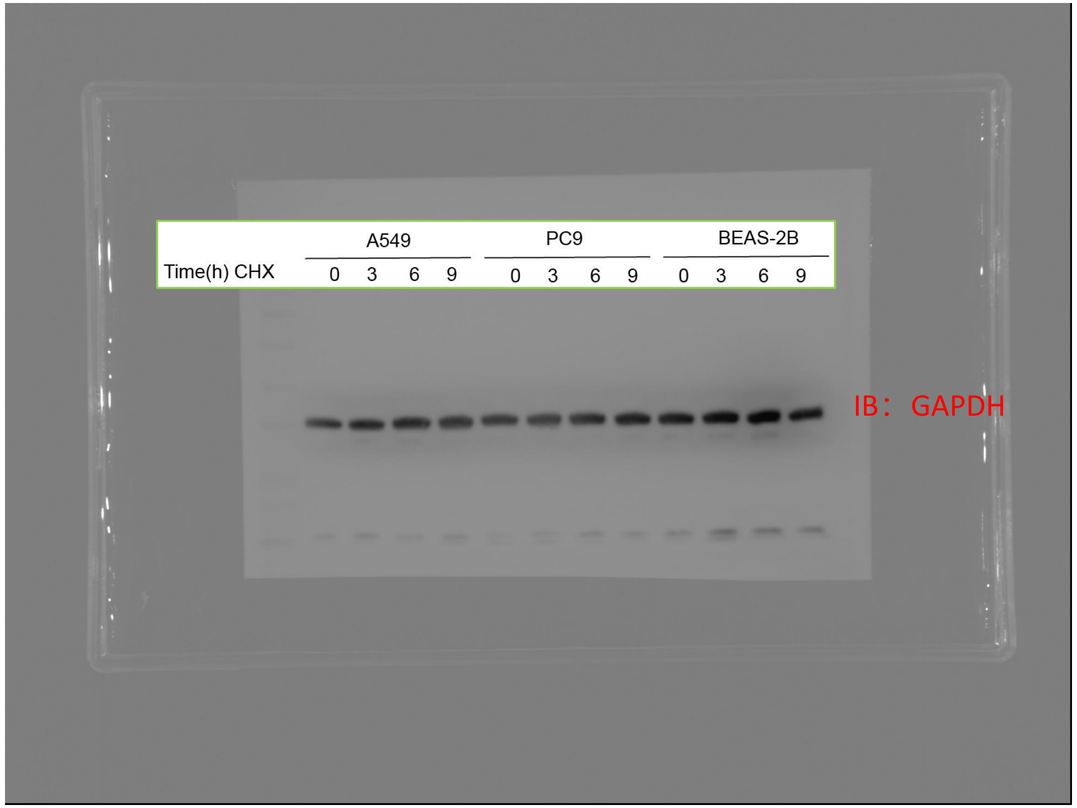

With marker
