# Supplementary material for: Lung Adenocarcinoma Promotes NETosis via the NPM1–TNFAIP6–CD44–SPP1 Axis
Source: Cancers (Basel). 2026 Mar 22;18(6):1023. doi: 10.3390/cancers18061023 (PMC13026014; doi:10.3390/cancers18061023)

Fig 3A

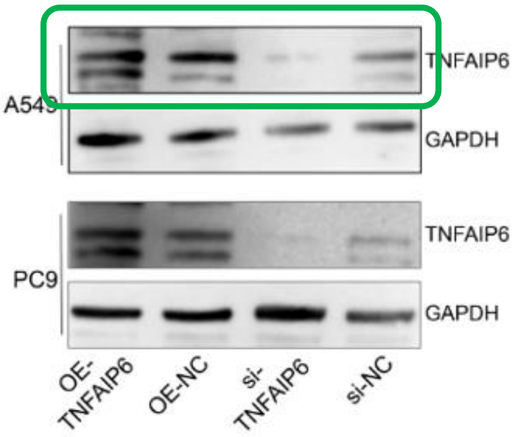
A549
TNFAIP6
GAPDH
PC9
TNFAIP6
GAPDH
OE-TNFAIP6
OE-NC
si-TNFAIP6
si-NC

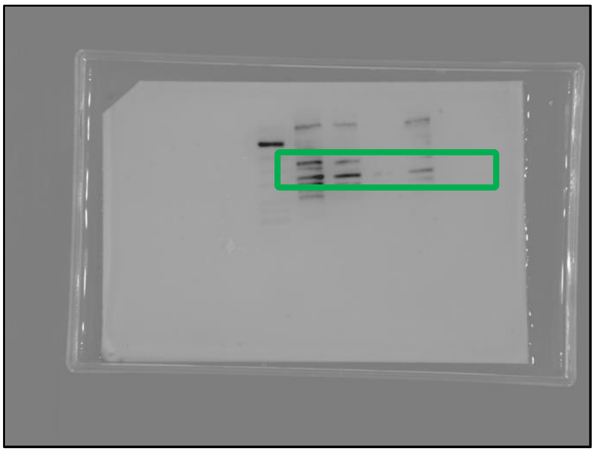

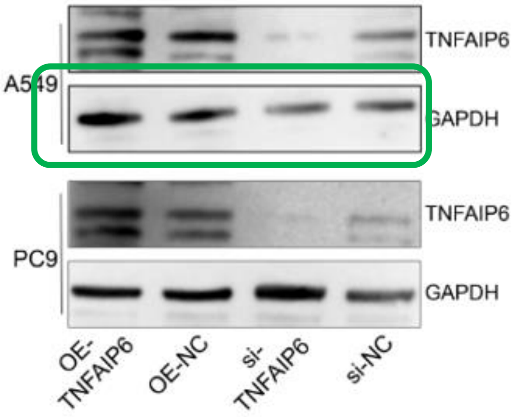
A549
TNFAIP6
GAPDH
PC9
TNFAIP6
GAPDH
OE-TNFAIP6
OE-NC
si-TNFAIP6
si-NC

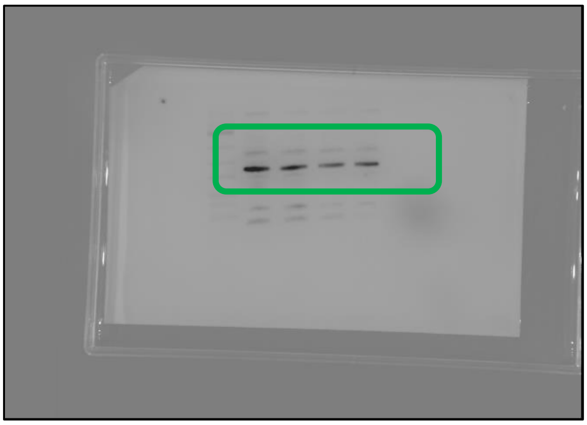

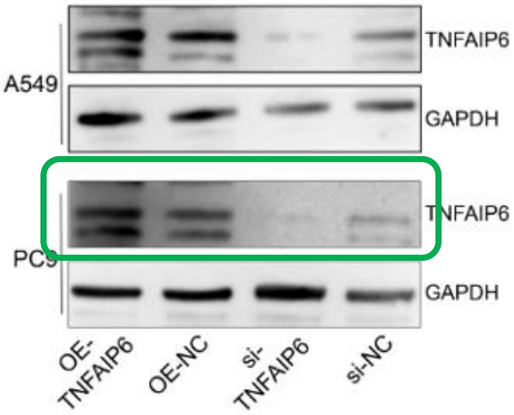
A549
TNFAIP6
GAPDH
PC9
TNFAIP6
GAPDH
OE-TNFAIP6
OE-NC
si-TNFAIP6
si-NC

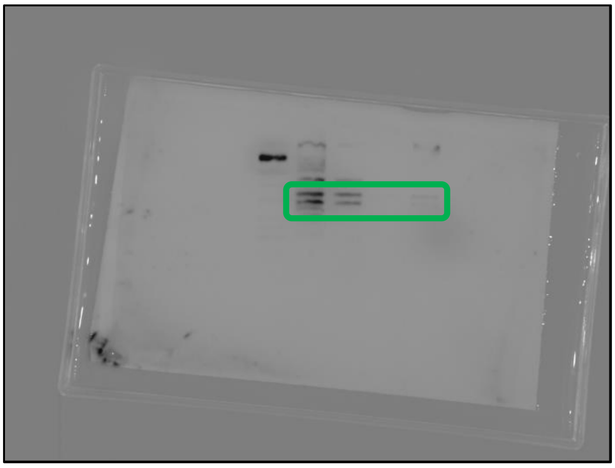

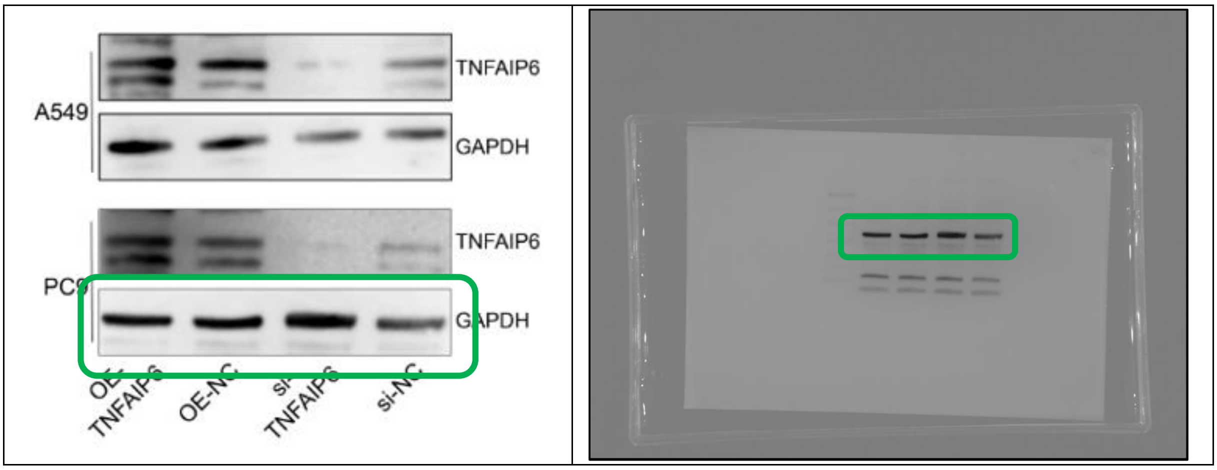
TNFAIP6
A549
GAPDH
TNFAIP6
GAPDH
OE-NC

Fig 3C

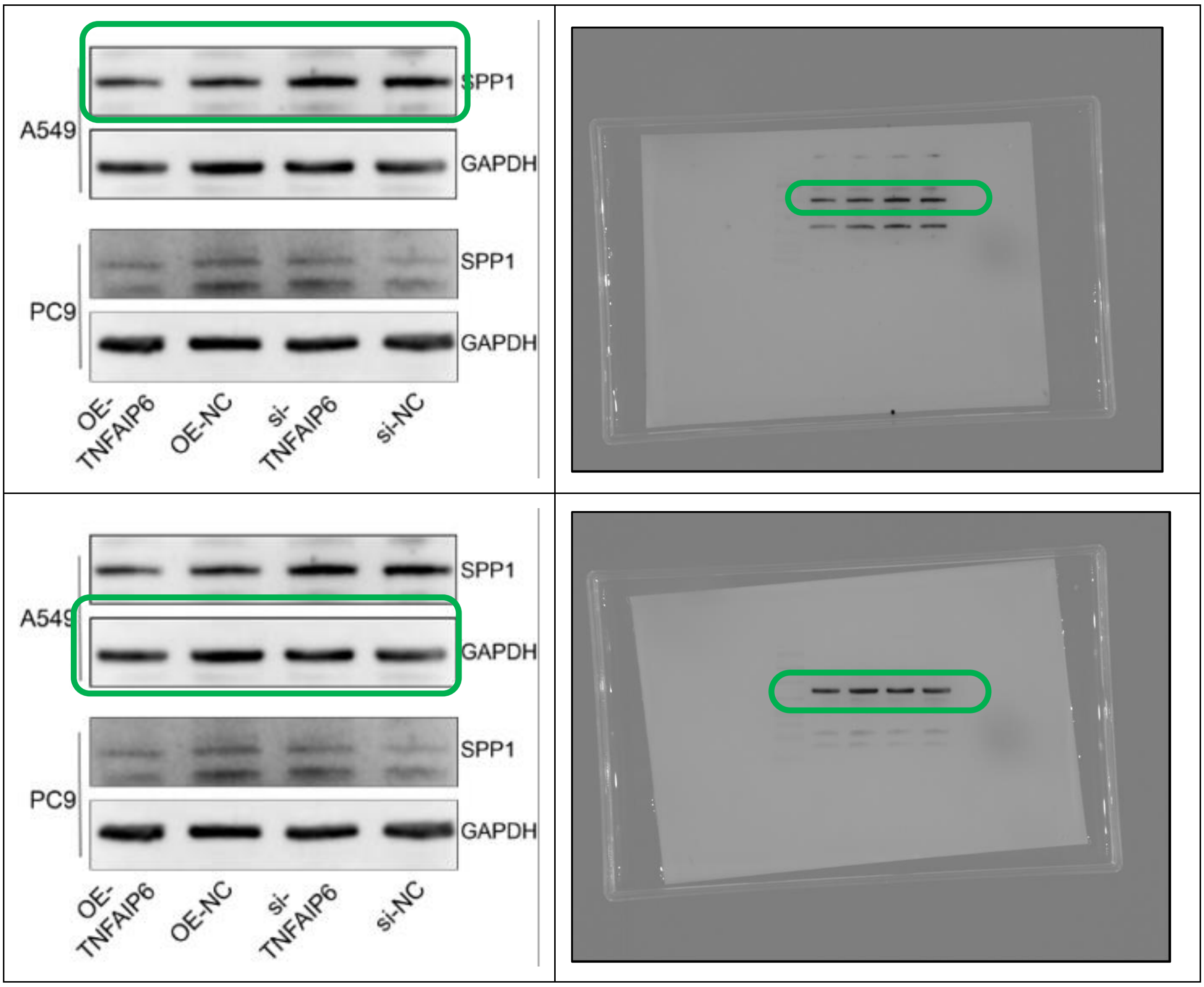
A549
SPP1
GAPDH
PC9
SPP1
GAPDH
OE-TNFAIP6
OE-NC
si-TNFAIP6
si-NC
SPP1
GAPDH
PC9
SPP1
GAPDH
OE-TNFAIP6
OE-NC
si-TNFAIP6
si-NC

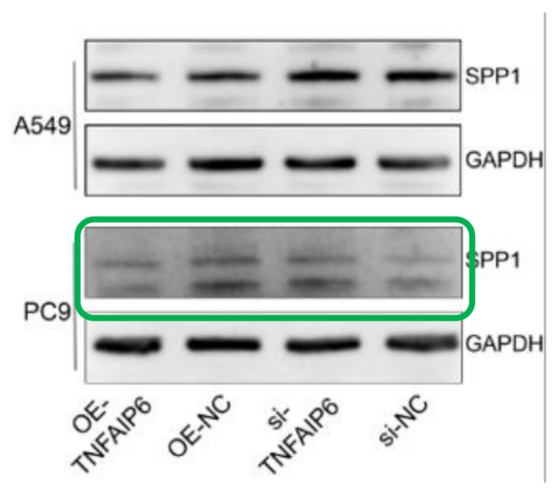
SPP1
A549
GAPDH
SPP1
PC9
GAPDH
OE-TNFAIP6
OE-NC
si-TNFAIP6
si-NC

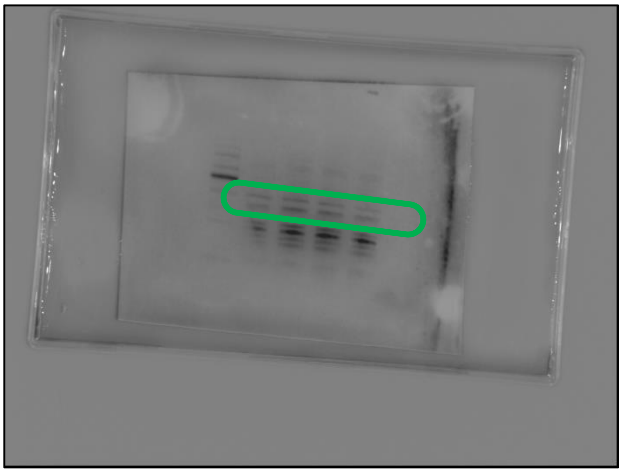

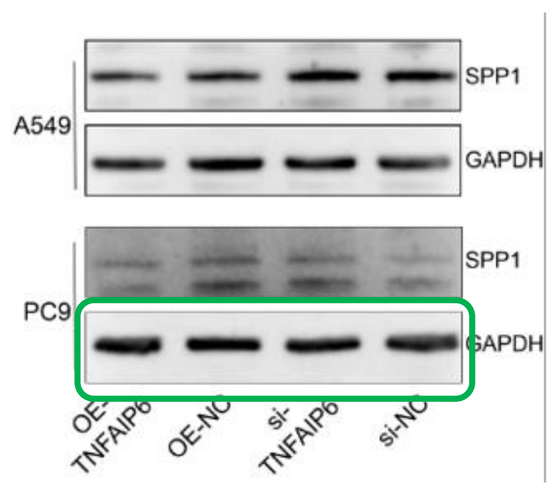
SPP1
A549
GAPDH
SPP1
PC9
GAPDH
OE-TNFAIP6
OE-NC
si-TNFAIP6
si-NC

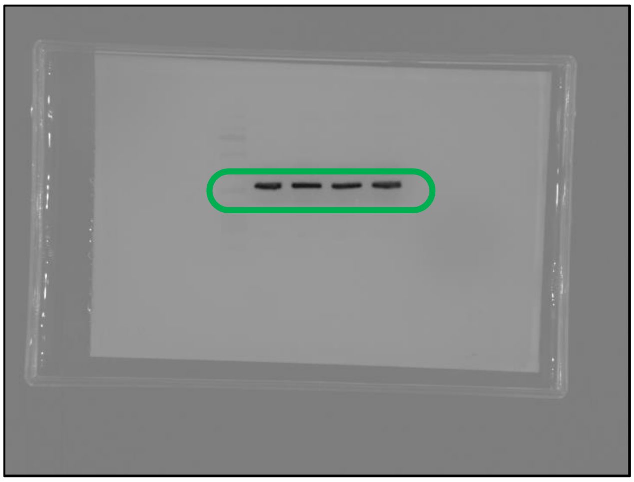

Fig 4C i

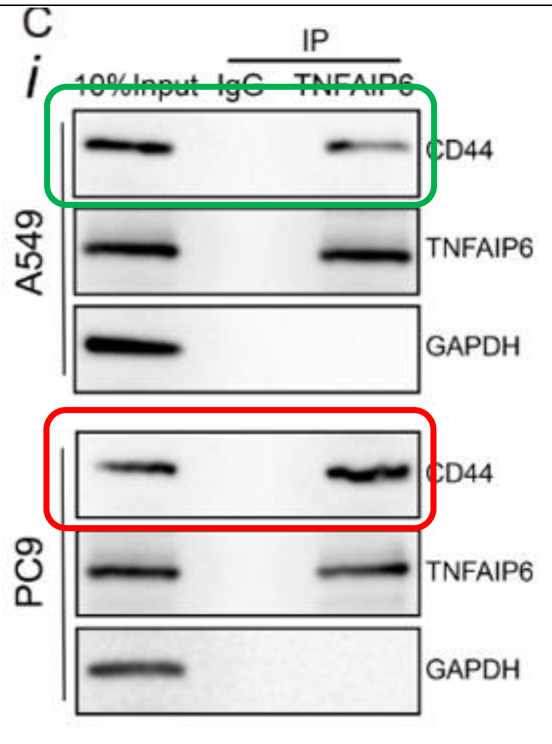
C
i
IP
10%Input
IgG
TNFAIP6
CD44
TNFAIP6
GAPDH
A549
CD44
TNFAIP6
GAPDH
PC9

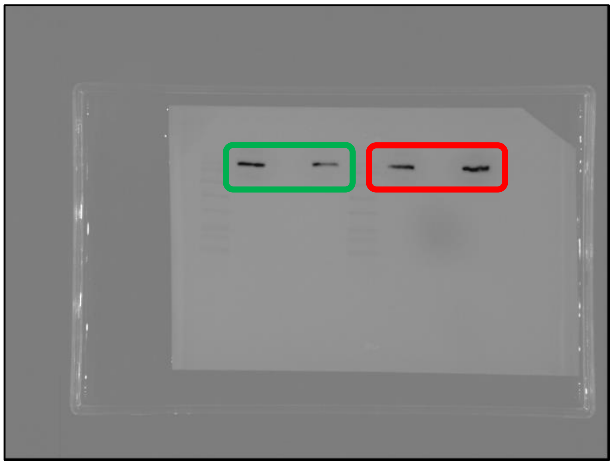

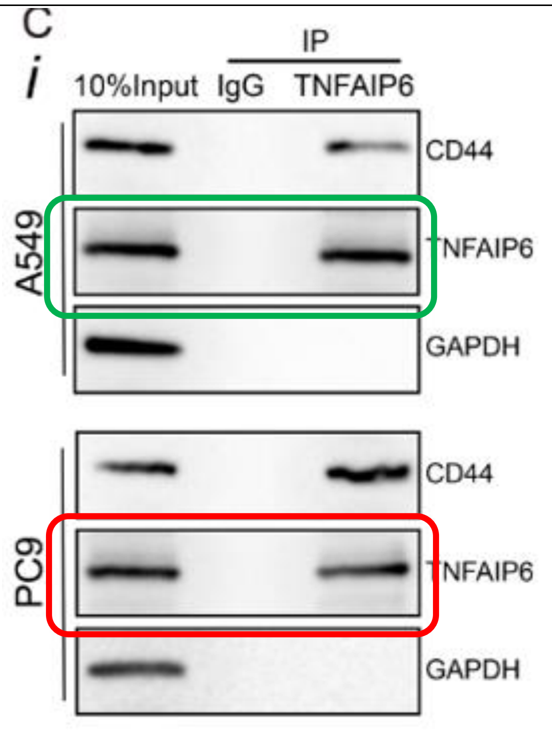
C
i
IP
10%Input
IgG
TNFAIP6
CD44
TNFAIP6
GAPDH
A549
CD44
TNFAIP6
GAPDH
PC9

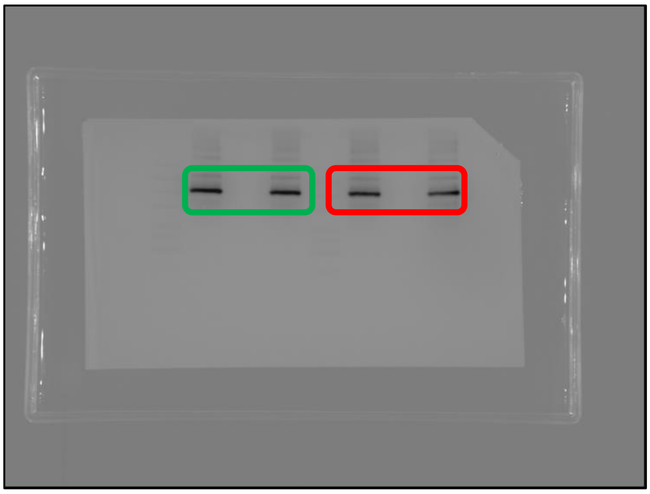

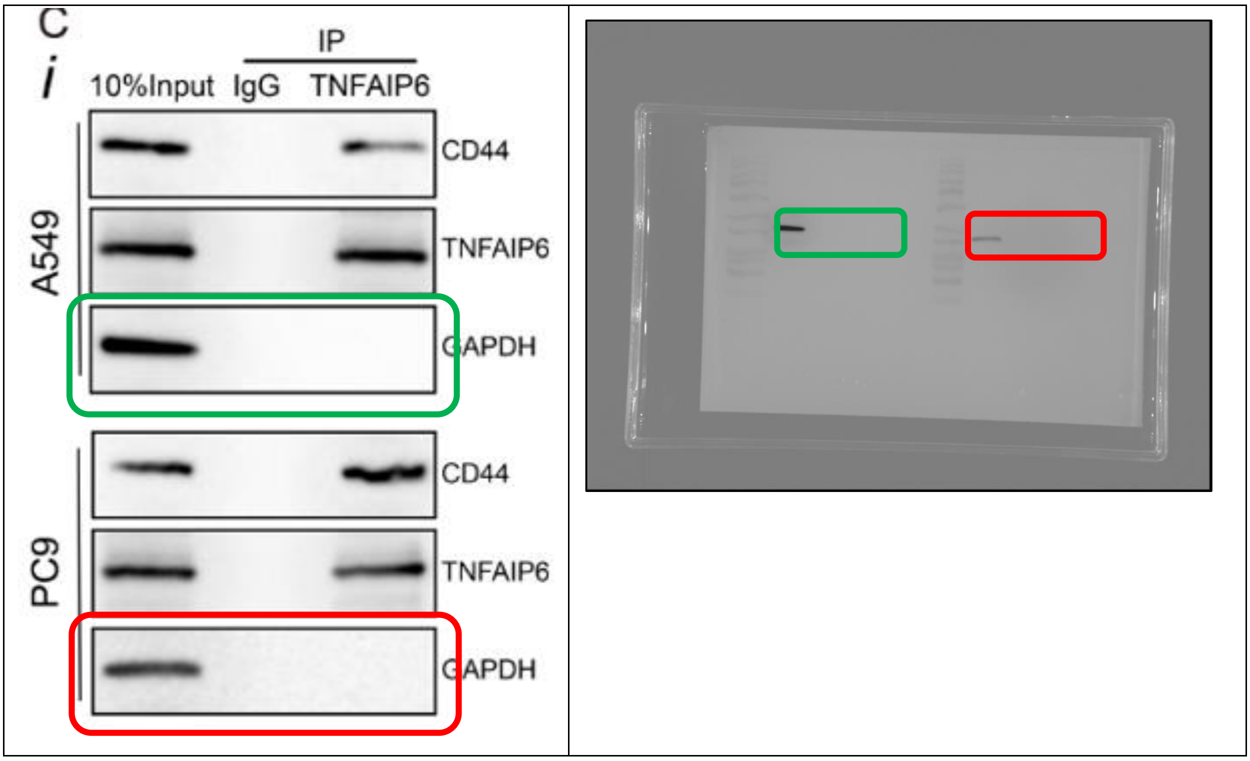
C
i
IP
10%Input
IgG
TNFAIP6
CD44
TNFAIP6
GAPDH
CD44
TNFAIP6
GAPDH
A549
PC9

Fig 4C i i

Fig 4C ii

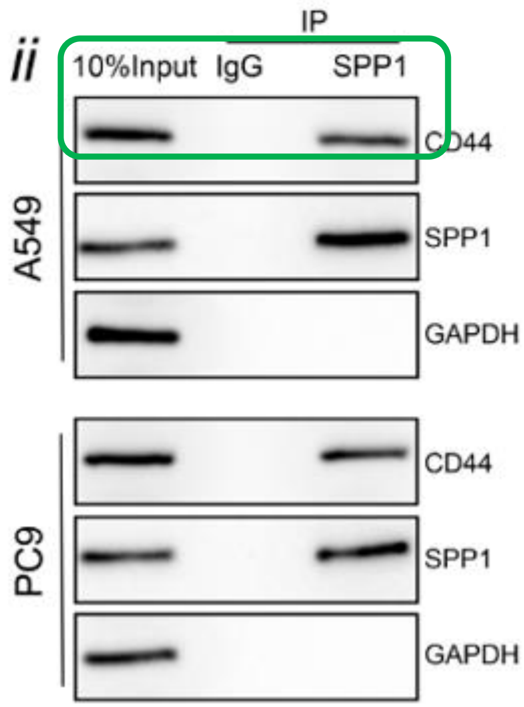

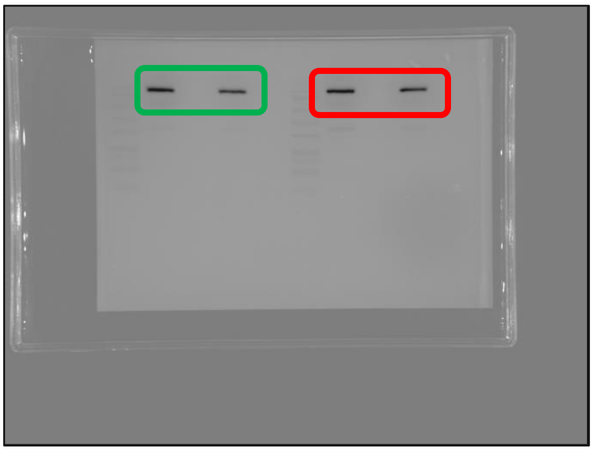

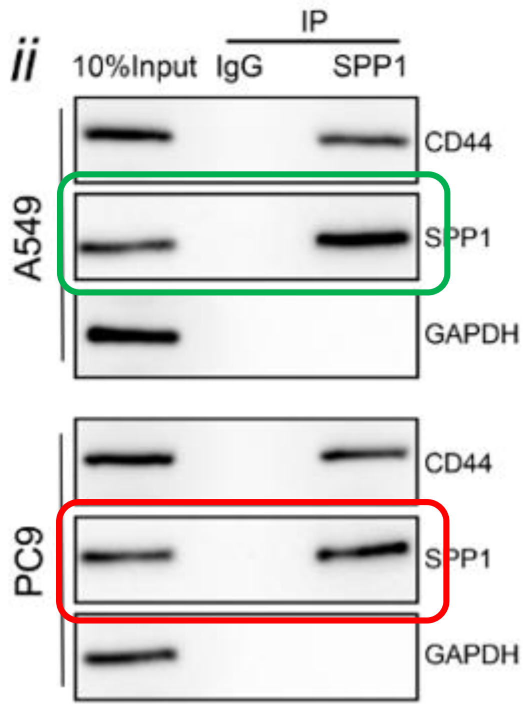

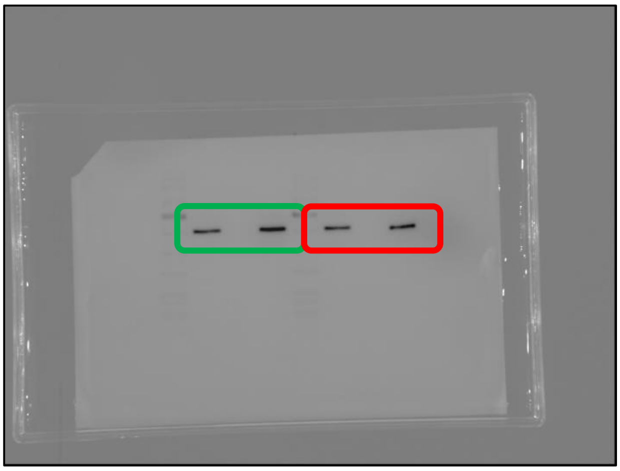

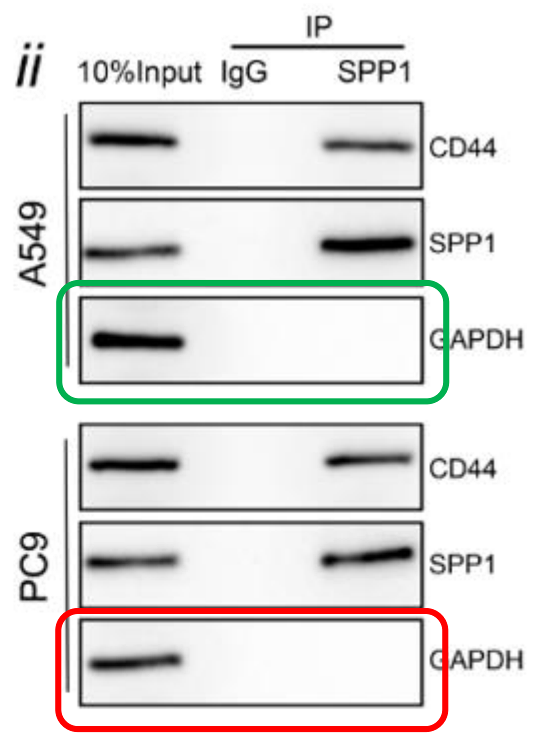
ii
IP
10%Input
IgG
SPP1
A549
CD44
SPP1
GAPDH
PC9
CD44
SPP1
GAPDH

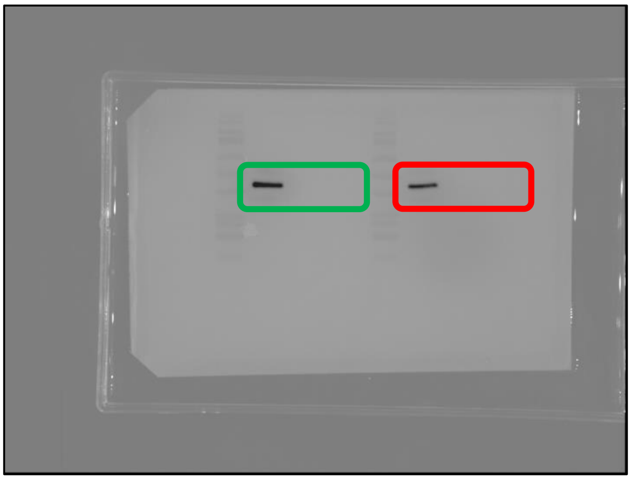

Fig 4D i

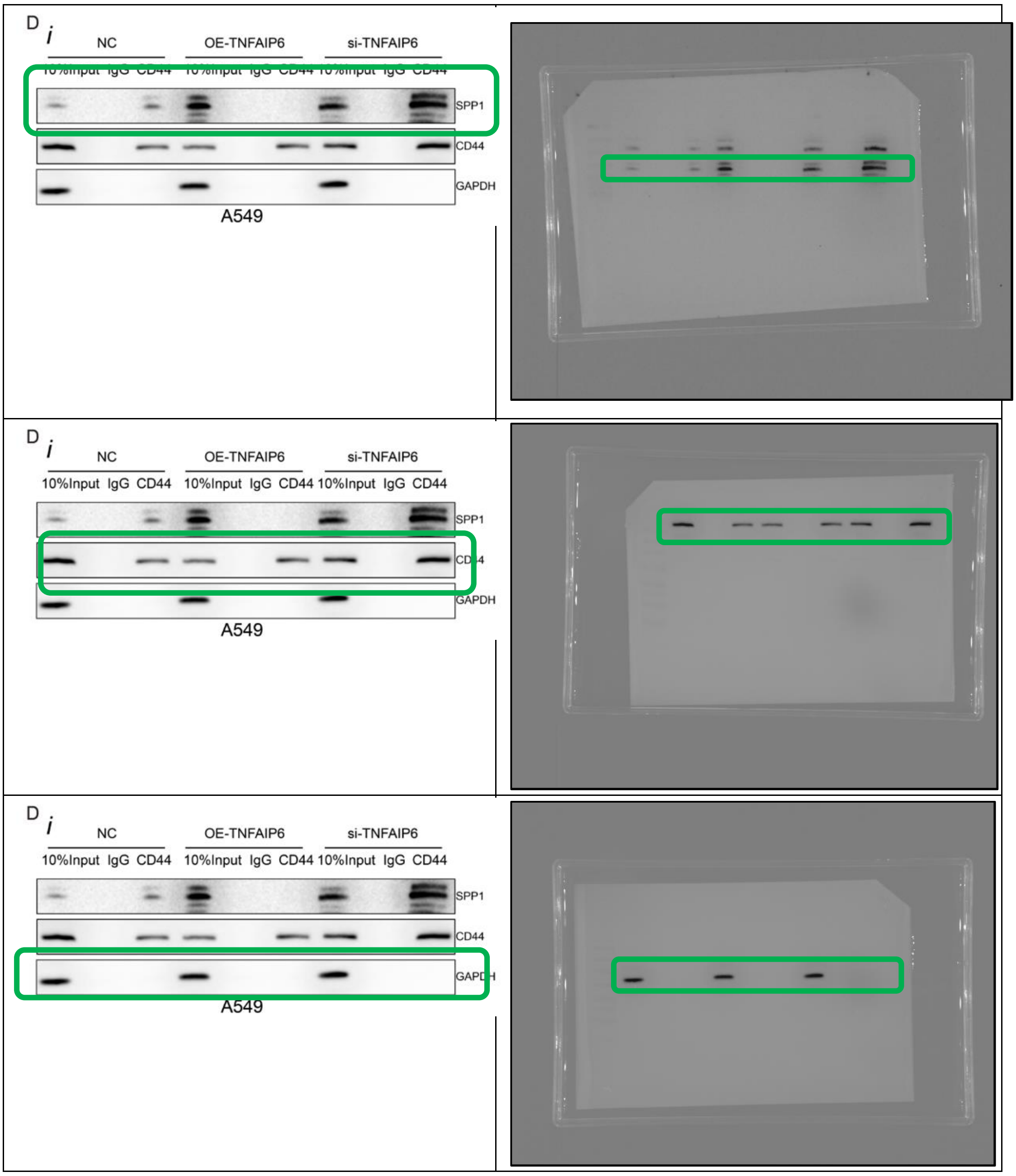
D
i
NC
OE-TNFAIP6
si-TNFAIP6
10%Input IgG CD44 10%Input IgG CD44 10%Input IgG CD44
SPP1
CD44
GAPDH
A549

Fig 4D ii

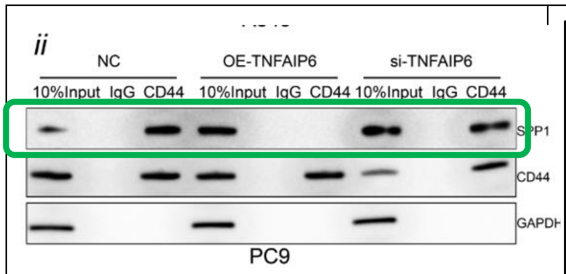
ii
NC
OE-TNFAIP6
si-TNFAIP6
10%Input IgG CD44 10%Input IgG CD44 10%Input IgG CD44
SPP1
CD44
GAPDH
PC9

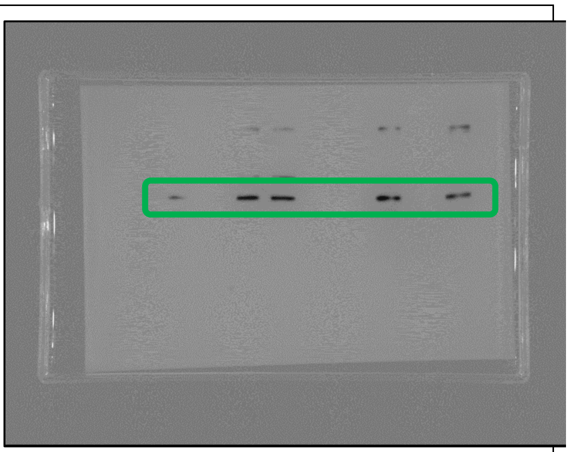

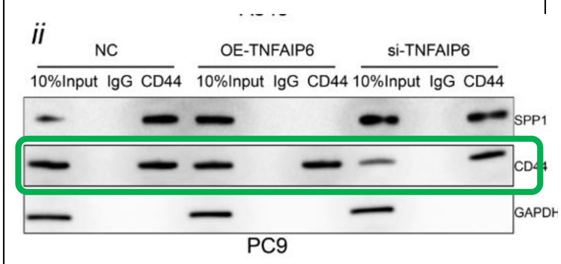
ii
NC
OE-TNFAIP6
si-TNFAIP6
10%Input IgG CD44 10%Input IgG CD44 10%Input IgG CD44
SPP1
CD44
GAPDH
PC9

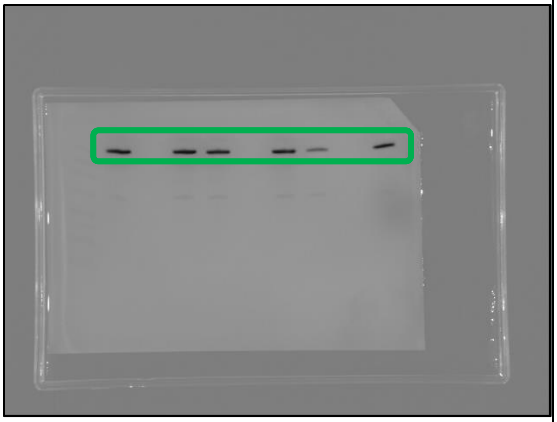

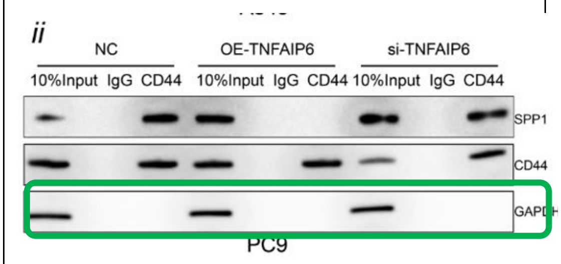
ii
NC
OE-TNFAIP6
si-TNFAIP6
10%Input IgG CD44 10%Input IgG CD44 10%Input IgG CD44
SPP1
CD44
GAPDH
PC9

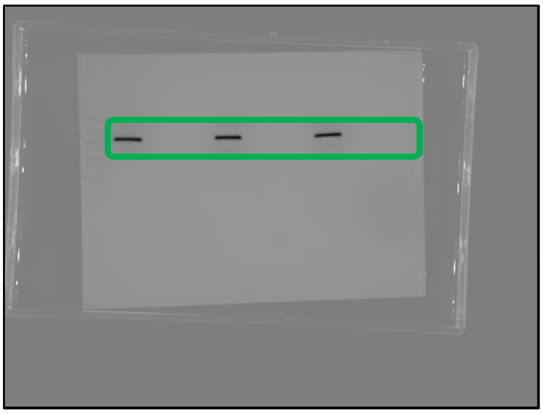

Fig 4E i

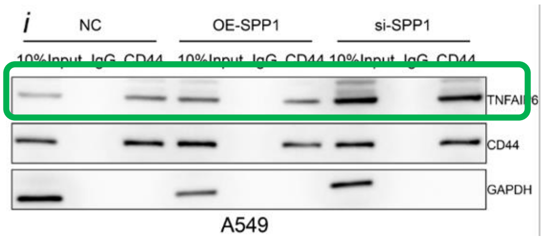

i
NC
OE-SPP1
si-SPP1
10%Input IgG CD44 10%Input IgG CD44 10%Input IgG CD44
TNFAIP6
CD44
GAPDH
A549

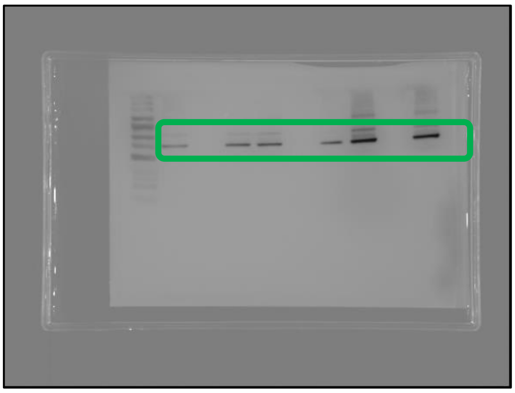

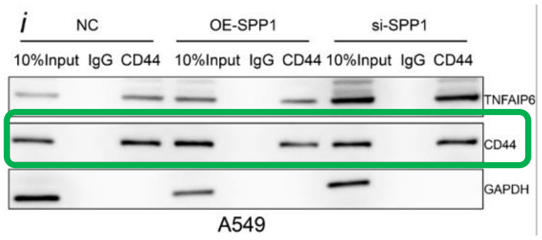

i
NC
OE-SPP1
si-SPP1
10%Input IgG CD44 10%Input IgG CD44 10%Input IgG CD44
TNFAIP6
CD44
GAPDH
A549

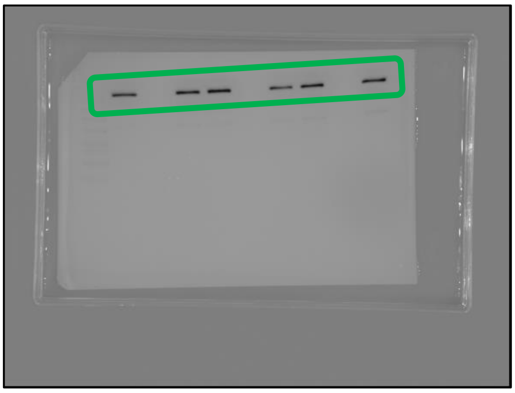

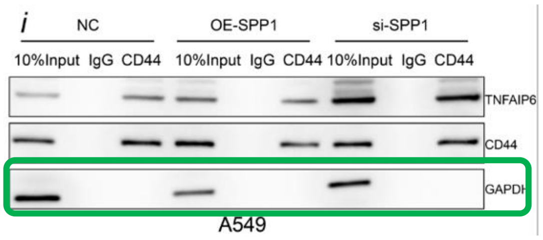

i
NC
OE-SPP1
si-SPP1
10%Input IgG CD44 10%Input IgG CD44 10%Input IgG CD44
TNFAIP6
CD44
GAPDH
A549

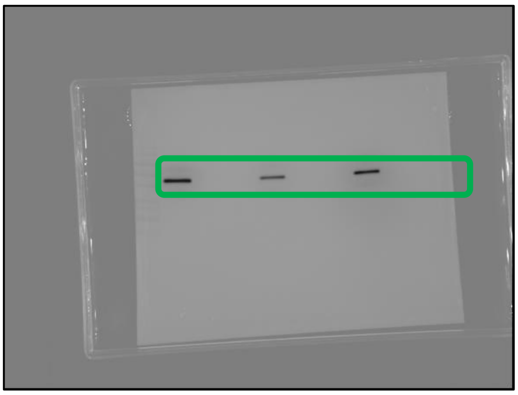

Fig 4E ii

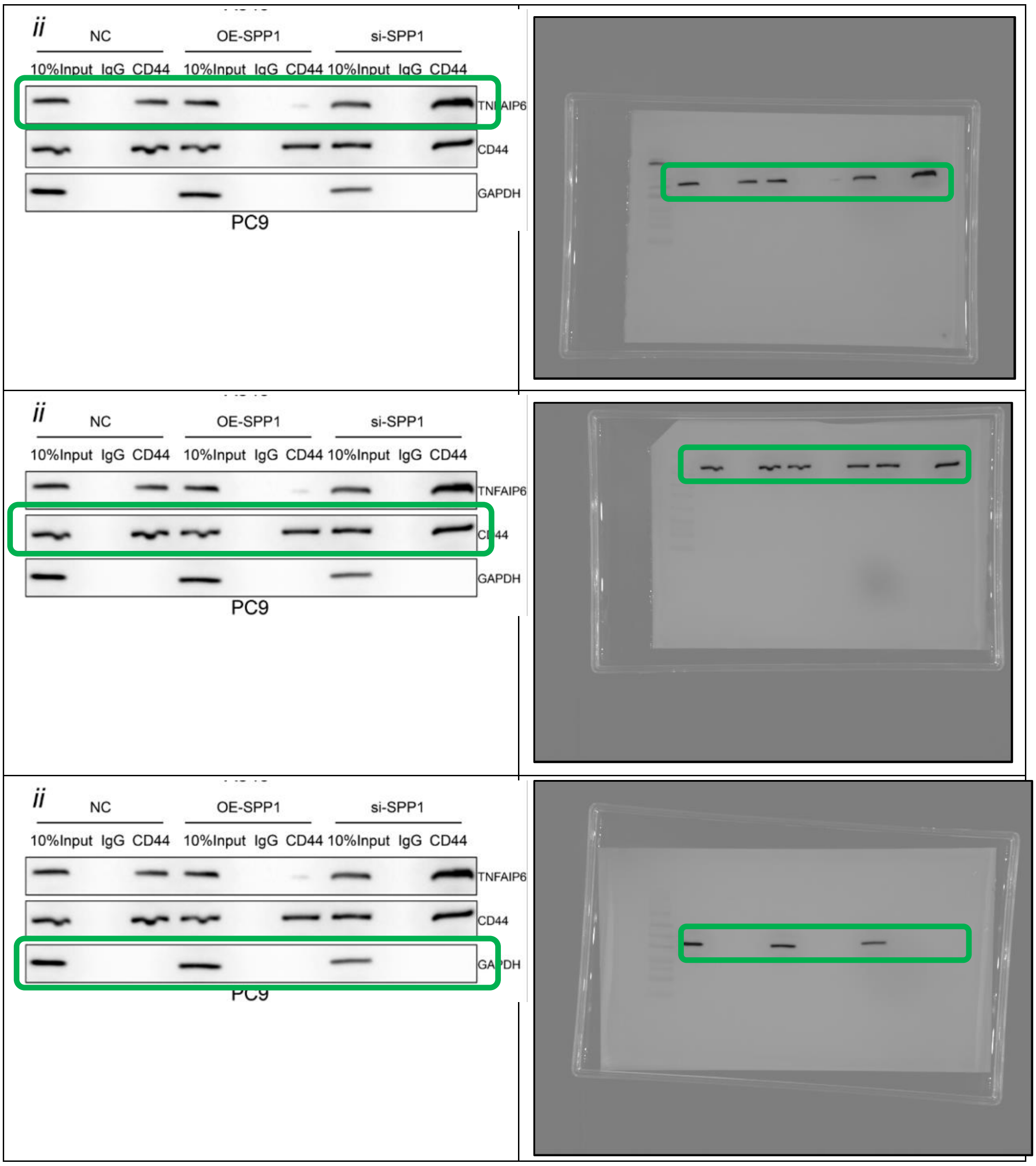
ii
NC
OE-SPP1
si-SPP1
10%Input IgG CD44 10%Input IgG CD44 10%Input IgG CD44
TNFAIP6
CD44
GAPDH
PC9

Fig 5C

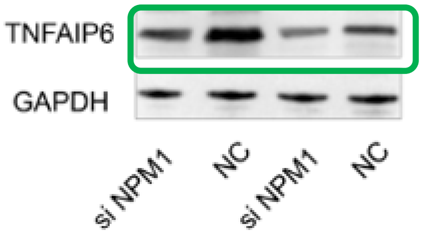
TNFAIP6
GAPDH
si NPM1
NC
si NPM1
NC

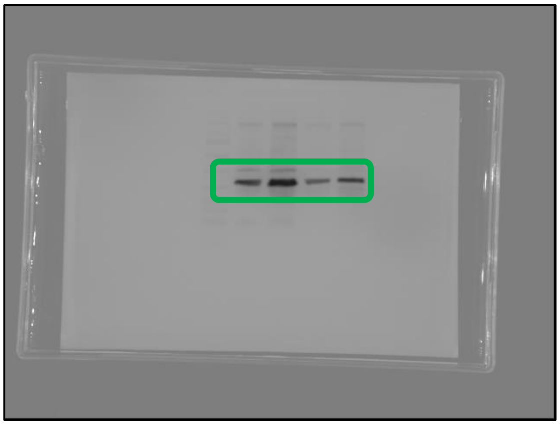

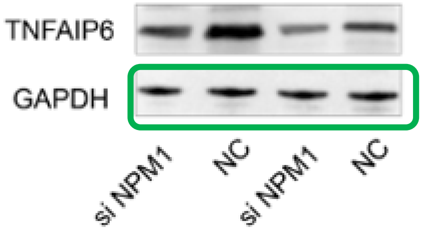
TNFAIP6
GAPDH
si NPM1
NC
si NPM1
NC

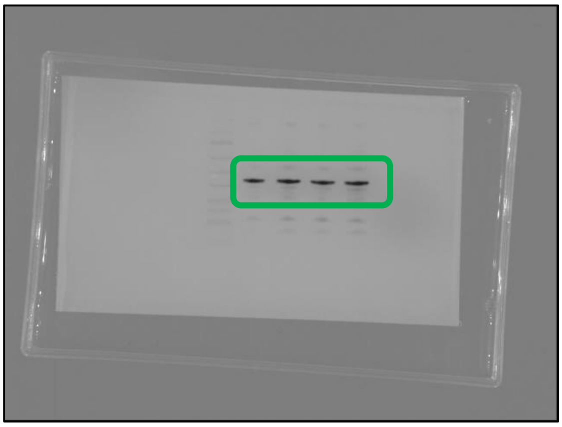

Fig 5D

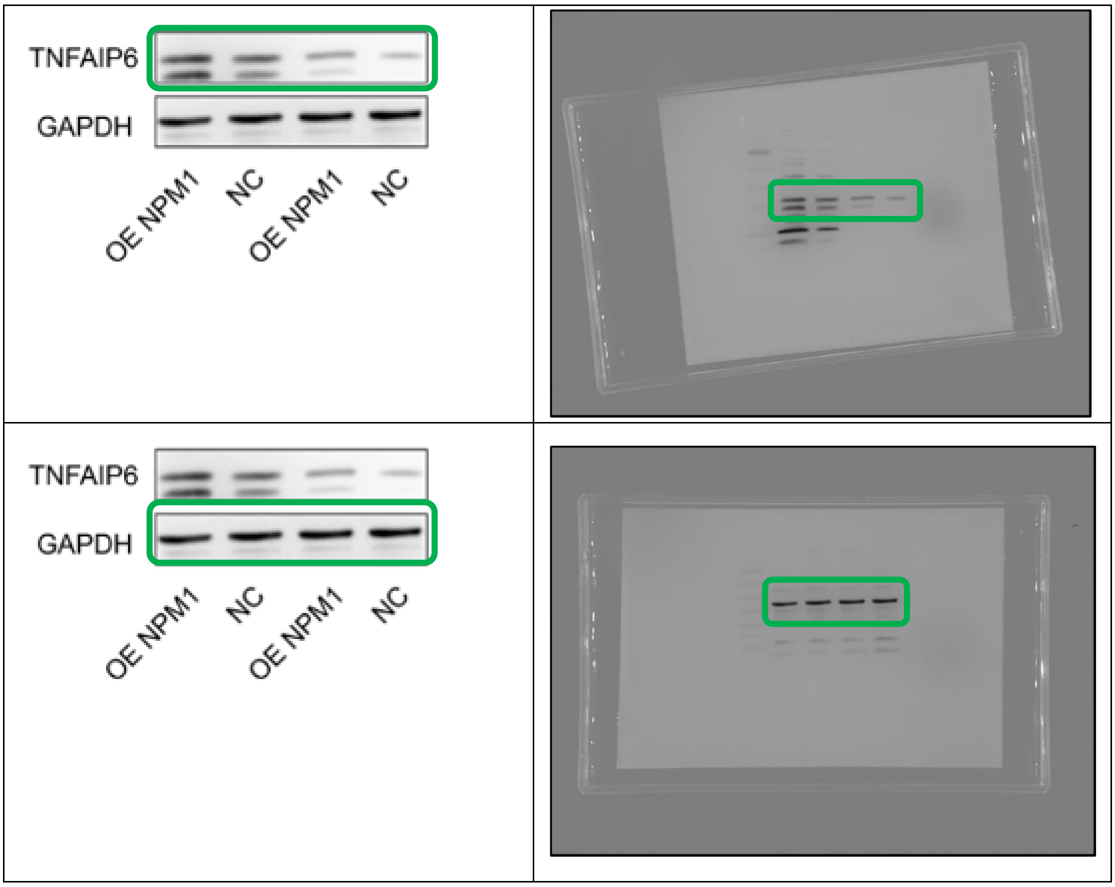

TNFAIP6
GAPDH
OE NPM1
NC
OE NPM1
NC
TNFAIP6
GAPDH
OE NPM1
NC
OE NPM1
NC

Fig 6A

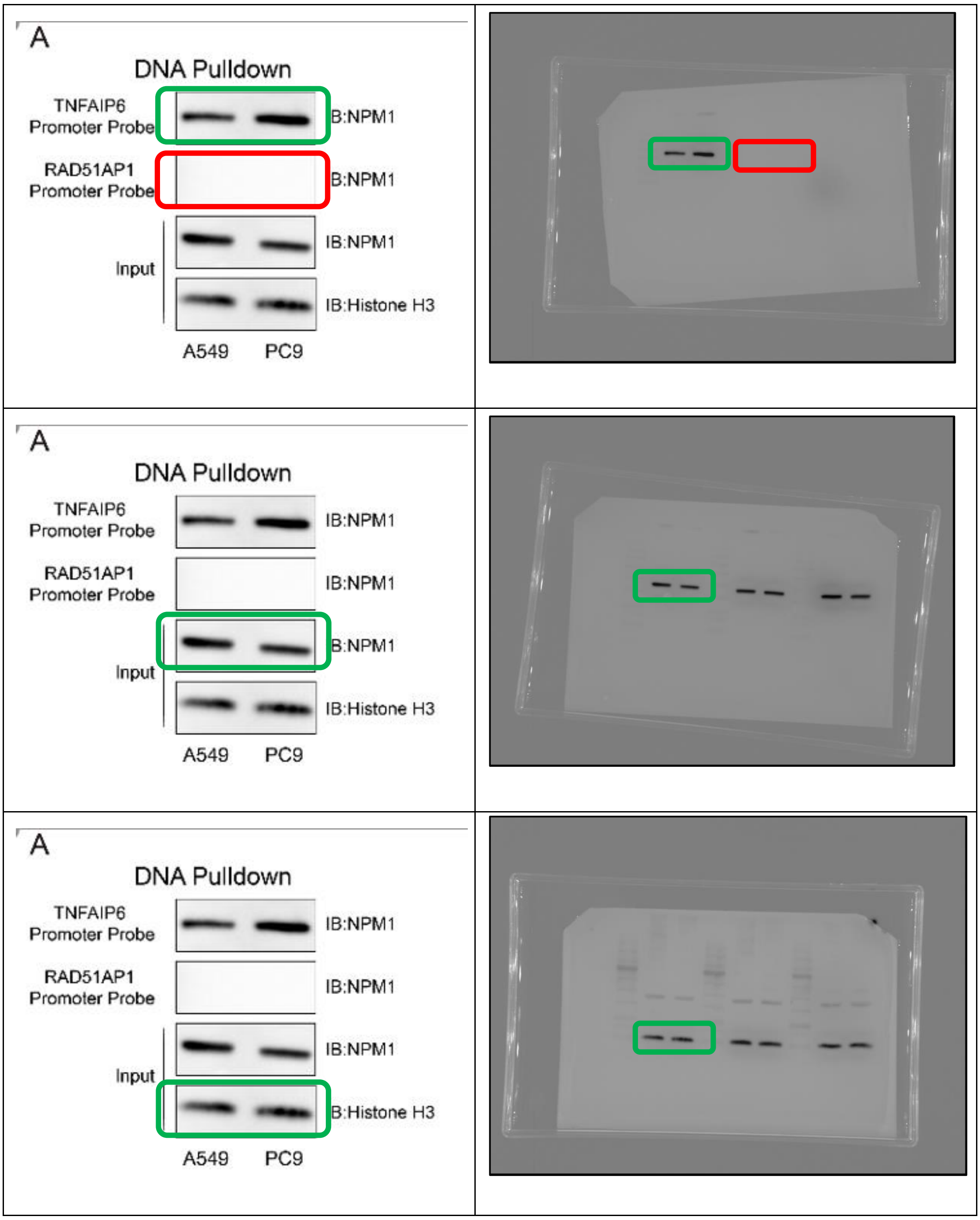
A
DNA Pulldown
TNFAIP6
Promoter Probe
RAD51AP1
Promoter Probe
Input
IB:NPM1
IB:NPM1
IB:NPM1
IB:Histone H3
A549
PC9

Fig 6B

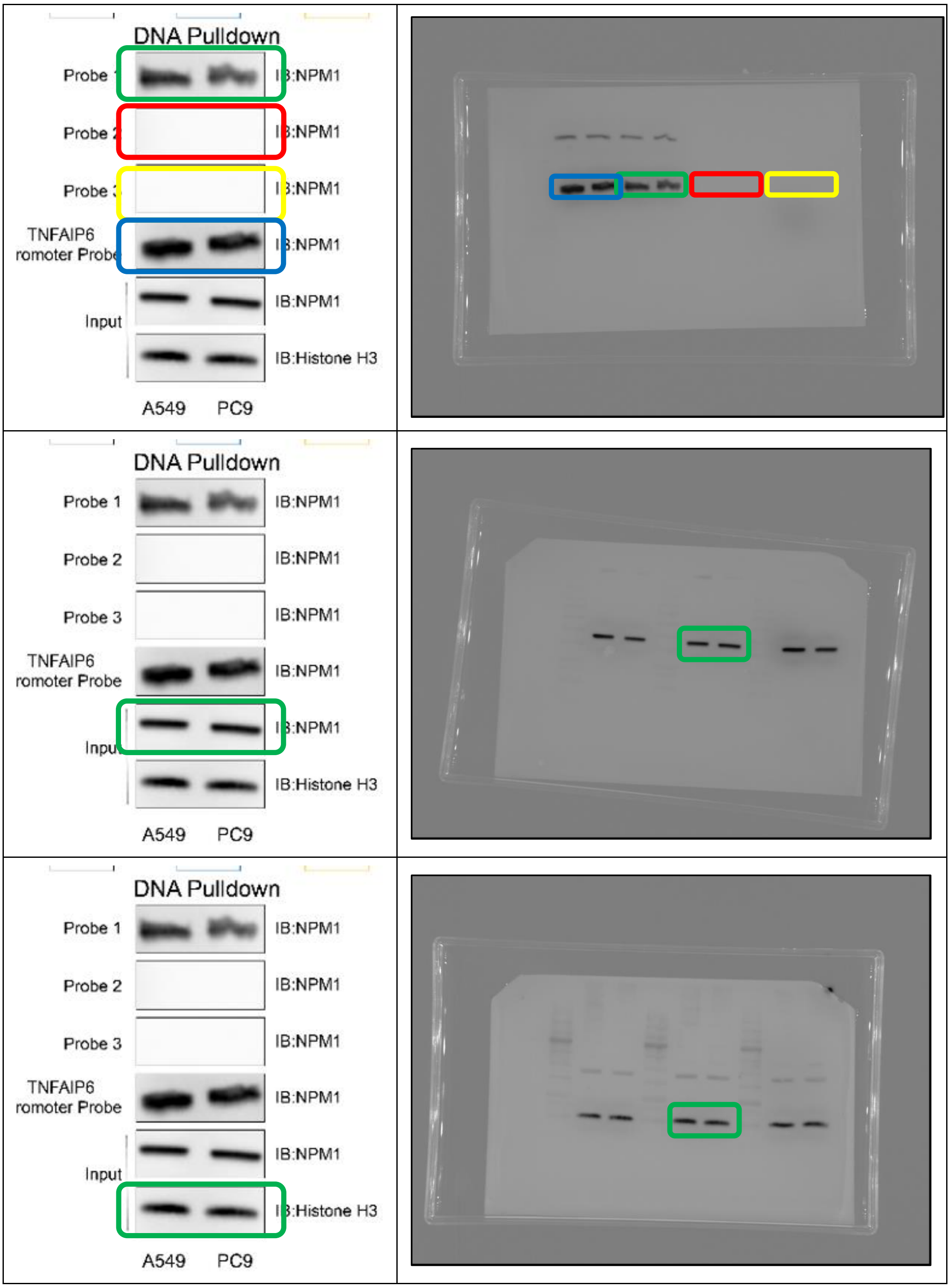
DNA Pulldown
Probe 1
Probe 2
Probe 3
TNFAIP6
romoter Probe
Input
IB:NPM1
IB:Histone H3
A549
PC9

Fig 6C

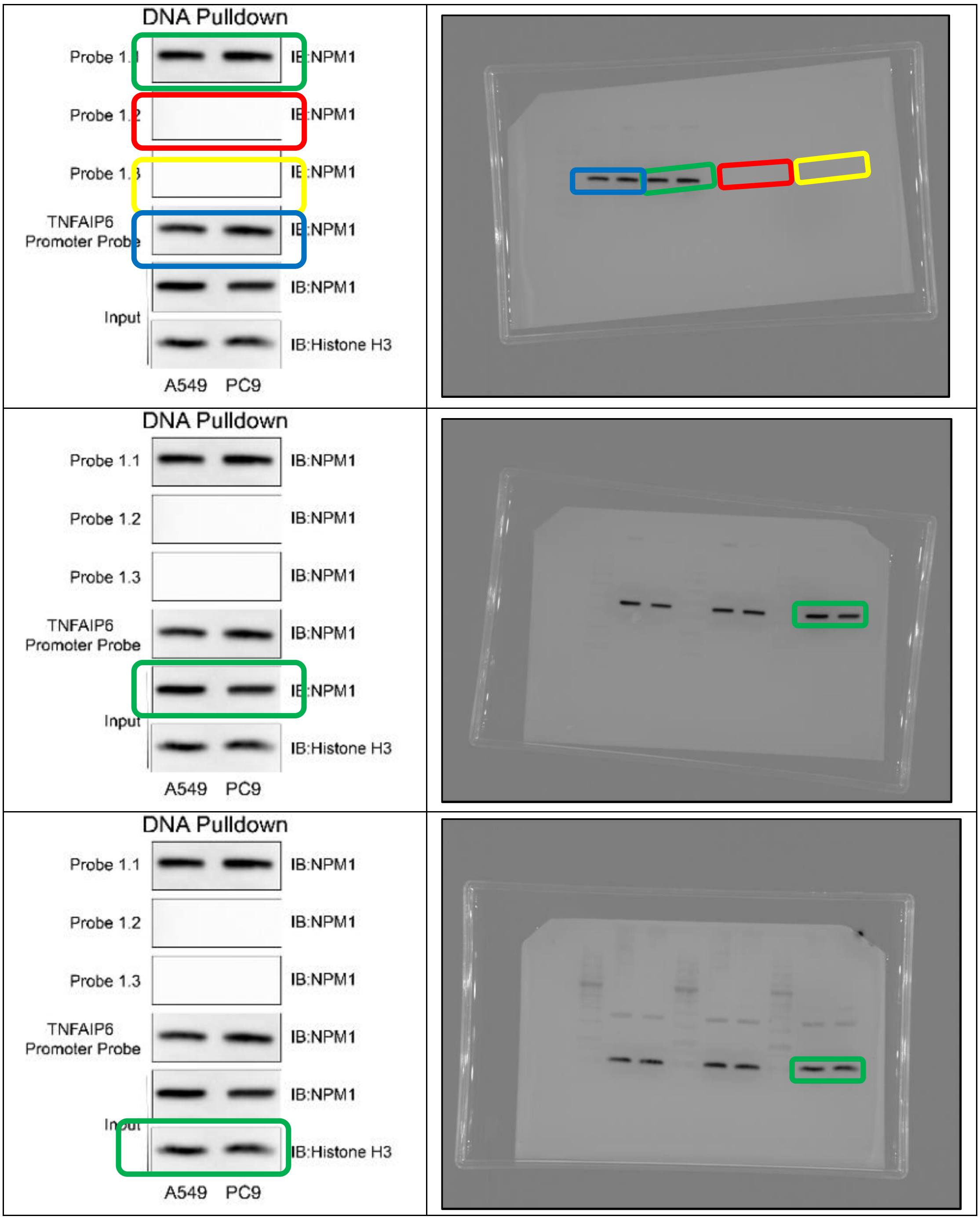
DNA Pulldown
Probe 1.1
IB:NPM1
Probe 1.2
IB:NPM1
Probe 1.3
IB:NPM1
TNFAIP6
Promoter Probe
IB:NPM1
IB:NPM1
Input
IB:Histone H3
A549
PC9
DNA Pulldown
Probe 1.1
IB:NPM1
Probe 1.2
IB:NPM1
Probe 1.3
IB:NPM1
TNFAIP6
Promoter Probe
IB:NPM1
IB:NPM1
Input
IB:Histone H3
A549
PC9
DNA Pulldown
Probe 1.1
IB:NPM1
Probe 1.2
IB:NPM1
Probe 1.3
IB:NPM1
TNFAIP6
Promoter Probe
IB:NPM1
IB:NPM1
Input
IB:Histone H3
A549
PC9

Supplementary Figure 4B

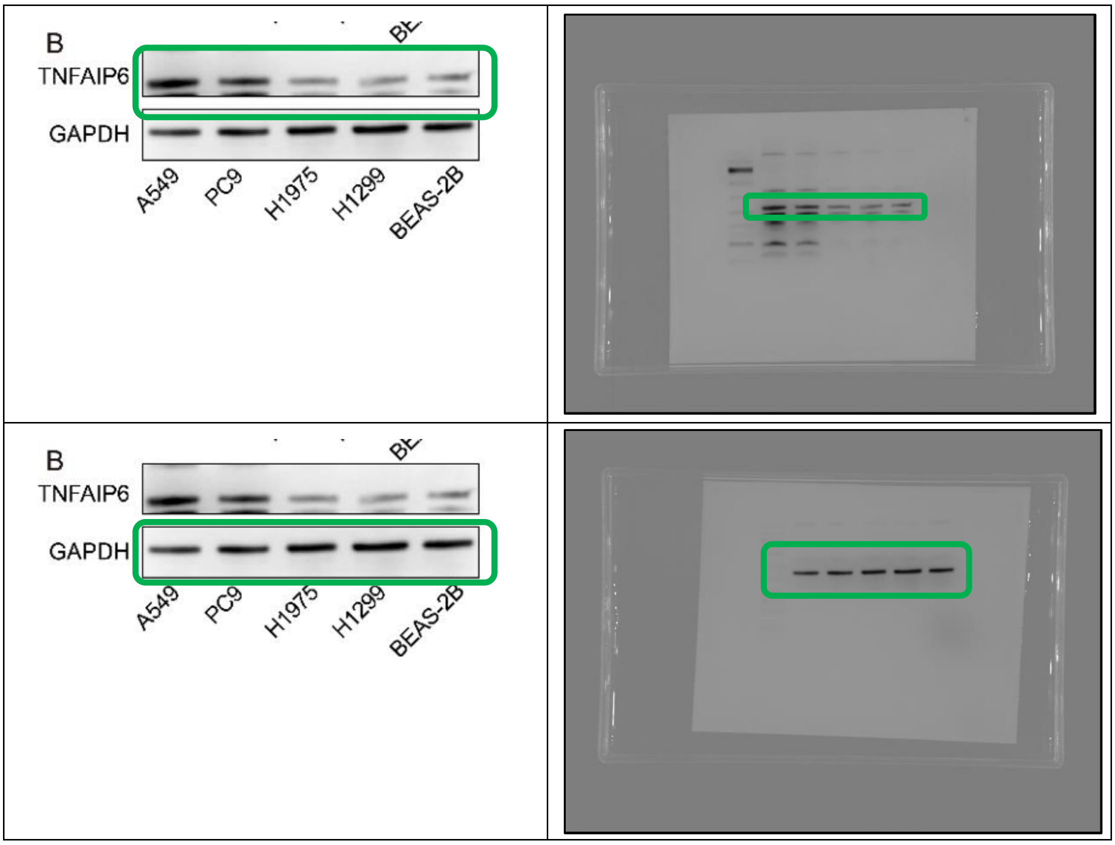

Supplementary Figure 4D

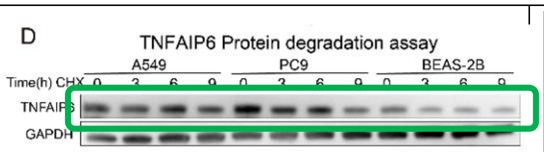

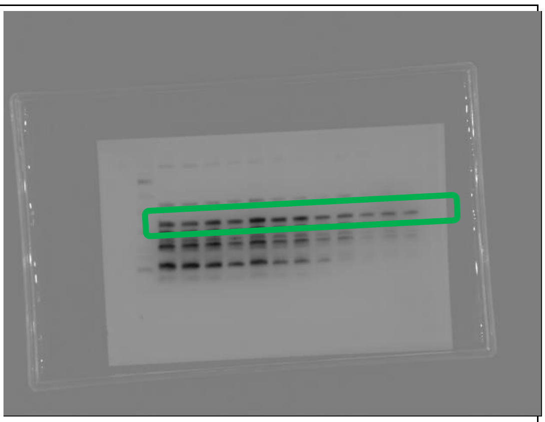

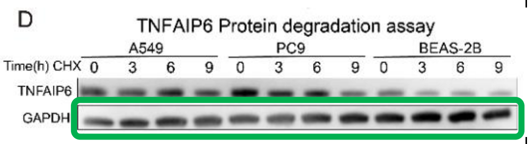

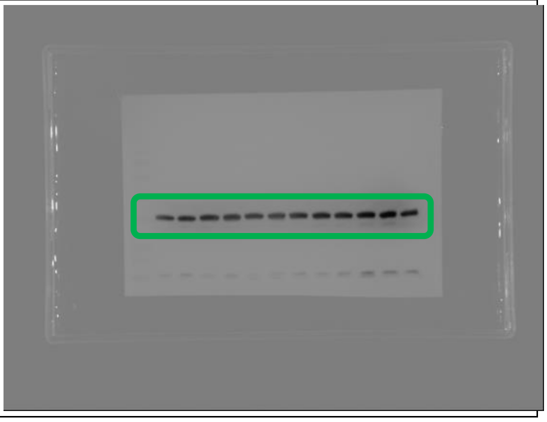

Supplement: Supplementary file 1 [file cancers-18-01023-s001.zip › Supplementary Materials S1/Western Blot Original Data Corresponding to Manuscript Results.pdf]
